# Intermetallic nanoassemblies potentiate systemic STING activation

**DOI:** 10.1126/science.adx1893

**Published:** 2026-05-07

**Authors:** Xingwu Zhou, Xiang Ling, Xiaoqi Sun, Ziye Wan, Tobias Dwyer, Timothy C. Moore, Quguang Li, Hannah E. Dobson, Qi Wu, Xiangbo Kong, Fang Xie, Xinran An, Jingyao Gan, Kaikai Wang, Young Seok Cho, Wang Gong, Katherine Dong, Jie Zhang, Mariko Takahashi, Cheng Xu, Swetha Kodamasimham, Jie Xu, Vilma Yuzbasiyan-Gurkan, Steven B. Chinn, Anna Schwendeman, Sharon C. Glotzer, Yu Leo Lei, James J. Moon

**Affiliations:** 1 Department of Pharmaceutical Sciences, University of Michigan, Ann Arbor, MI, USA.; 2 Biointerfaces Institute, University of Michigan, Ann Arbor, MI, USA.; 3 Department of Chemical Engineering, University of Michigan, Ann Arbor, MI, USA.; 4 Center for Advanced Models for Translational Sciences and Therapeutics, University of Michigan Medical Center, University of Michigan Medical School, Ann Arbor, MI, USA.; 5 Department of Head and Neck Surgery, The University of Texas MD Anderson Cancer Center, Houston, TX, USA.; 6 Department of Microbiology, Genetics, and Immunology, Michigan State University, East Lansing, MI, USA.; 7 Department of Small Animal Clinical Sciences, Michigan State University, East Lansing, MI, USA.; 8 Department of Otolaryngology-Head and Neck Surgery, University of Michigan, Ann Arbor, MI, USA.; 9 Department of Biomedical Engineering, University of Michigan, Ann Arbor, MI, USA.; 10 Rogel Cancer Center, University of Michigan, Ann Arbor, MI, USA.

## Abstract

Natural systems use metal ions to form ordered structures that regulate biological processes, inspiring the rational design of nanotherapeutics. The cyclic guanosine monophosphate–adenosine monophosphate synthase–stimulator of interferon genes (cGAS-STING) pathway drives antitumor immunity but has been difficult to activate systemically owing to poor pharmacology and toxicity. Here, we report CRYSTAL, a structurally ordered intermetallic nanoparticle for potent systemic STING activation. CRYSTAL self-assembles from manganese ions intercalated with cyclic dinucleotides, enabling precise structural control. At an ultralow intravenous dose (0.003 milligrams per kilogram), CRYSTAL activated STING in mice, dogs, and nonhuman primates without cytokine release syndrome. CRYSTAL induced robust tumor regression in advanced murine and rabbit models, remodeled immunosuppressive environments, and promoted host STING–dependent CD8^+^ T cell priming. CRYSTAL activated interferon responses in human head and neck squamous cell carcinoma biopsies, underscoring its translational potential for cancer immunotherapy.

## Abstract (summary)

**INTRODUCTION:**

Metal ions play essential roles in biology by organizing biomolecules into precise structures that enable specialized functions, such as oxygen transport and mineral formation. These examples illustrate how metal-directed assembly can orchestrate biological activity. One immune pathway that could benefit from such design is the cyclic guanosine monophosphate–adenosine monophosphate synthase–stimulator of interferon genes (cGAS-STING) pathway, which detects cytosolic DNA and triggers interferon responses that support antitumor immunity. Clinical translation of STING agonists has been challenging, and safe and effective systemic STING activation remains an unmet need in cancer immunotherapy.

**RATIONALE:**

Systemically delivered small molecules require high doses and often cause widespread immune activation and toxicity, whereas nanoparticle formulations improve delivery but generally fail to elicit strong immune responses in tumors. We hypothesized that structurally ordered, metal-based nanostructures could overcome these limitations.

**RESULTS:**

Inspired by natural metal-biomolecule assemblies, we developed CRYSTAL (crystal-like STING-activating nanoassemblies), in which manganese ions (Mn^2+^) organize cyclic dinucleotide STING agonists into stable nanoscale structures that are subsequently coated with lipids. This design was intended to stabilize the STING agonist, improve systemic exposure, promote immune cell engagement, and enhance STING signaling at substantially lower doses. We evaluated CRYSTAL using computational modeling, in vivo studies across multiple species, and ex vivo experiments in human tumor tissues. Computational modeling predicted spontaneous formation of well-defined manganese–STING agonist nanostructures. In mice, intravenous CRYSTAL induced robust and sustained interferon responses at ultralow doses. CRYSTAL selectively activated myeloid cells, remodeled immunosuppressive tumor environments, and promoted strong CD8^+^ T cell responses in a host STING–dependent manner, resulting in durable tumor regression in advanced mouse models and aggressive rabbit tumors. Across species, CRYSTAL achieved potent immune activation with a favorable safety profile: Dogs and nonhuman primates exhibited strong interferon induction without significant toxicity. CRYSTAL also activated interferon signaling in freshly resected human head and neck cancer samples, demonstrating translational relevance.

**CONCLUSION:**

CRYSTAL introduces a structurally ordered metal-based nanomedicine that enables safe, potent systemic STING activation across species. By combining metal-directed molecular assembly with lipid encapsulation, CRYSTAL overcomes key delivery and toxicity barriers that have limited STING-based cancer immunotherapies. More broadly, this work establishes metal-organized nanostructures as a powerful design principle for immune modulation and supports CRYSTAL’s potential for clinical translation. □

## Editor’s summary

Molecules that activate innate immune signaling through the stimulator of interferon genes (STING) pathway are of interest for cancer therapies. However, the use of agonists developed for this purpose has been hampered because they generally require high doses and can be toxic. Zhou et al. generated an intermetallic nanoparticle called CRYSTAL that self-assembles when manganese ions intercalate with cyclic dinucleotides (see the Perspective by Liao and Irvine). CRYSTAL was able to activate STING, could control tumors at low doses in mice and rabbits, and had a promisingly low toxicity profile. Rather than acting on cancer cells, CRYSTAL activated STING in myeloid cells preferentially, promoting antitumor T cell responses. —Sarah H. Ross

## Graphical Abstract

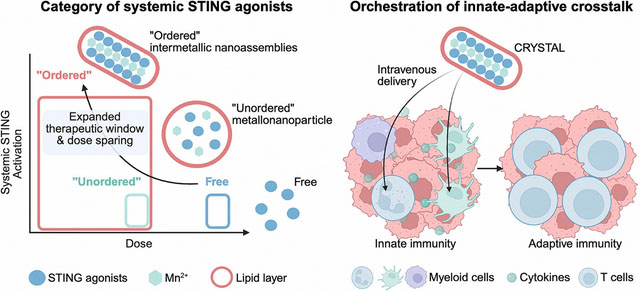

**Metal-organized nanoassemblies amplify systemic STING activation.** Biomimetic use of metal ions leads to the development of a new category of STING-activating metallonanoparticle, called CRYSTAL, with pronounced dose sparing and exceptional antitumor efficacy in multiple preclinical models. After systemic administration, CRYSTAL selectively activates myeloid cells and expands antitumor CD8^+^ T cells, leading to robust STING-driven tumor regression. [Figure created with BioRender.com]

Natural systems have developed various strategies to harness metal ions in essential biological processes. For example, iron (Fe^2+^) ions coordinate structurally with hemoglobin subunits for oxygen transportation and storage (1). Magnesium (Mg^2+^) ions regulate crystallization of hydroxyapatite nanowires in dental enamel to form the hardest tissue in the human body (2, 3), whereas zinc (Zn^2+^) ions facilitate the in vivo storage of self-assembled insulin protein in the crystal form (4). Beyond biological applications, structurally ordered metal ion doping has driven advancements in fuel cells (5), catalysts (6), and photonic devices (7). Inspired by these intricate natural and engineered designs, we sought to develop a class of structurally ordered intermetallic nanoparticles for biological applications.

In particular, cyclic guanosine monophosphate (GMP)–adenosine monophosphate (AMP) synthase (cGAS) recognizes cytoplasmic DNA to produce 2,3-cGAMP, which binds to the stimulator of interferon genes (STING) protein and activate its downstream signaling pathways (8, 9). Activation of STING initiates interferon (IFN) regulatory factor 3 (IRF3) and nuclear factor κB (NF-κB) pathways to generate type I IFN (IFN-I) and proinflammatory cytokines and chemokines that are essential for establishing innate and adaptive antitumor immune responses (10). However, translating STING activation into effective cancer immunotherapy has proven challenging. Cyclic dinucleotides (CDNs), STING activators, exhibit poor pharmacological properties, restricting their use as localized therapeutics (11) and limiting their efficacy against advanced cancers. In addition, clinical trials of intratumoral STING agonists have shown only modest responses in patients (12). Limited responses from intratumoral treatment have driven the development of small-molecule STING agonists for systemic cancer immunotherapy (13–15); however, these molecules require high doses, pose toxicity risks, and suffer from a narrow therapeutic window. To overcome these issues, nanoparticle systems have been developed for targeted STING activation (16), improved pharmacodynamic (PD) profiles through polymeric nanoparticles (17, 18), enhanced tumor penetration through lipid nanodiscs (19), and increased STING activation through manganese metal ions (Mn^2+^) (20) or polyvalent STING agonists (21). Despite these advances, nanoparticle systems provide only limited dose-sparing effects and exhibit dominant liver accumulation upon systemic administration, thus failing to achieve potent immune activation in tumor tissues and ultimately reducing therapeutic efficacy. To address these issues, we examined how metal-organized nanostructures can overcome pharmacological and immunological barriers.

## Results

### Self-assembled CRYSTAL induces systemic STING activation in mice

**Fig. 1.**
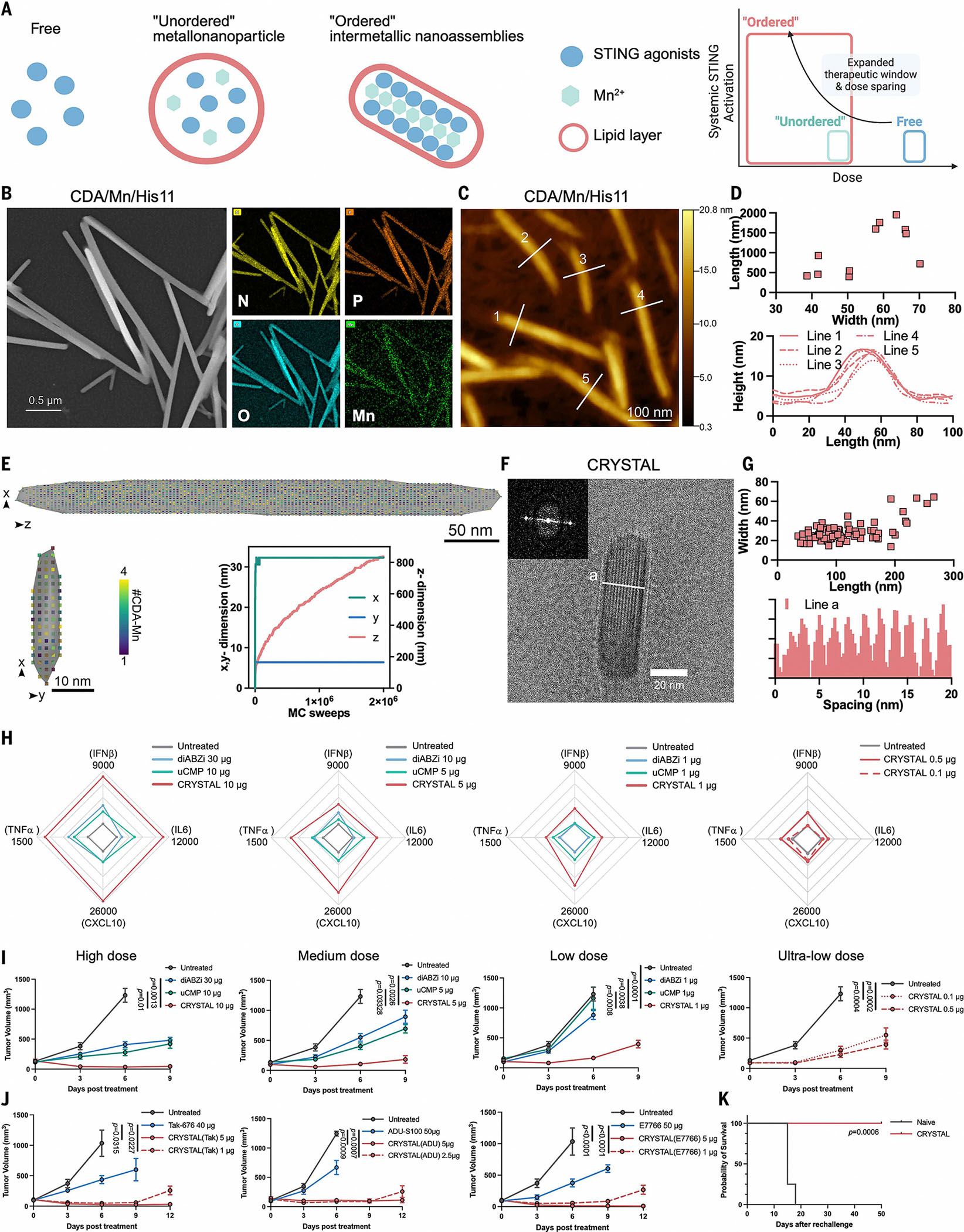
Self-assembled CRYSTAL induces systemic STING activation in mice. (**A**) Schematic comparison of three categories of STING agonists currently under clinical or preclinical development, including free drugs, lipid nanoparticles, or particles with structurally ordered nanoassemblies. (**B**) STEM-EDX analysis of CDA/Mn/His11 nanoassemblies. N (yellow), P (orange), O (cyan), and Mn (green) are representative elements in CDA/Mn/His11 nanoassemblies (scale bar: 0.5 μm). (**C**) AFM images of CDA/Mn/His11 nanoassemblies (scale bar: 100 nm). (**D**) Width and length analysis of CDA/Mn/His11 based on TEM images. Height analysis of CDA/Mn/His11 based on AFM images. (**E**) Snapshots from MC simulations of the CDA/Mn/His11 nanoassembly showing front (top) and cross-sectional (bottom left) views at 500,000 MC sweeps. The growth dimensions of the CDA/Mn/His11 nanoassembly (bottom right). (**F**) Cryo-EM images of CRYSTAL, with an inset showing the FFT image (scale bar: 20 nm). (**G**) Statistical analysis of CRYSTAL dimensions based on cryo-EM images (top) and analysis of nanoassembly spacings along the line marked “a” in (F) (bottom). (**H**) Radar plots of systemic STING activation profiles of diABZi (30, 10, and 1 μg), uCMP (10, 5, and 1 μg), and CRYSTAL (10, 5, 1, 0.5, and 0.1 μg) in B16F10 tumor–bearing C57BL/6 mice. Levels of IFN-β, TNF-α, CXCL10, and IL-6 were measured by ELISA 4 hours after intravenous administration. (**I**) Investigation of antitumor efficacy of diABZi, uCMP, and CRYSTAL in B16F10 tumor–bearing C57BL/6 mice. Treatment with the indicated dosage was performed on D0, D4, and D8 via intravenous administration. (**J**) Investigation of antitumor efficacy of CRYSTALs loaded with various STING agonists, including Tak-676, ADU-S100, and E7766 in B16F10 tumor–bearing C57BL/6 mice. Treatment with the indicated dosage was performed on D0, D4, and D8 via intravenous administration. (**K**) Surviving mice from the CRYSTAL (10 μg CDA) treatment group and naïve mice were inoculated with 2.5 × 10^5^ B16F10 cells on D60, and their survival was monitored. The data represent the mean with *n* = 3 to 5 (H) or the mean ± SEM with *n* = 3 to 5 (I), *n* = 4 or 5 (J), and *n* = 4 to 7 (K) biologically independent samples. Shown is a representative experiment from two independent experiments; the independent repeats of (I) and (J) are shown in fig. S37. The data were analyzed by two-way ANOVA [(I) and (J)] with Tukey’s multiple comparison post hoc test for tumor growth curve, or by log-rank (Mantel-Cox) test for survival curve (K).

Cyclic-di-AMP (CDA), a bacterial CDN, interacted with Mn^2+^ (Mn) and an 11-mer of histidine (His11, used as a metal coordinating ligand) to form structurally ordered CDA/Mn/His11 ribbonlike nanoassemblies (Fig. 1A). STEM-EDX (scanning transmission electron microscopy–energy dispersive x-ray analysis) confirmed the copresence of CDA (element P) and Mn^2+^ within the nanoribbons (Fig. 1B). To elucidate the assembly mechanism, we examined the physical properties of CDA/His11 and CDA/Mn/His11 nanocomplexes. Whereas CDA/His11 self-assembled into thin fibers, the addition of Mn^2+^ followed by brief sonication resulted in the formation of nanoribbons (fig. S1, A to C). Strong interactions between CDA and Mn were overcome by the addition of positively charged His11 (fig. S1D), leading to electrostatic repulsion and improved colloidal stability (22). Fourier transform infrared (FTIR) spectroscopy revealed intermolecular interactions stabilizing the CDA/Mn/His11 nanoassemblies, as evidenced by a red shift and peak broadening in the 1200 to 1300 cm^−1^ and 1600 to 1700 cm^−1^ regions (fig. S1E). Atomic force microscopy (AFM) further confirmed the ribbonlike morphology of the CDA/Mn/His11 nanoassemblies, measuring ~11 nm in thickness (Fig. 1, C and D, and fig. S2). These findings demonstrate that specific self-assembly conditions drive the formation of distinct nanoassemblies, contrasting with the amorphous clustering observed under other conditions (7, 20, 23).

To investigate the forces governing the assembly of these anisotropic nanostructures, we simulated the self-assembly process of CDA/Mn/His11 using a Monte Carlo (MC) growth algorithm. The molecular constituents of the nanoribbons were coarse-grained into building blocks, with each block representing a His11 peptide complexed with a variable number of CDA-Mn units along the x direction (fig. S3). Growth along the x direction was determined by the number of CDA-Mn complexes that bind to His11, whereas growth in the y direction was constrained by interaction energies on the order of a few $k_{B}T$ (thermal energy scale) per building block (parameters in table S1), limiting expansion to only a few layers, consistent with experimental observations. In contrast, growth in the z direction was modeled as occurring under strong, irreversible binding conditions, as the experimentally observed long, uniform ribbons suggested a preferential binding direction along the z axis. Beyond these short-range attractive interactions, we assumed that His11 was fully protonated, resulting in long-range electrostatic repulsion between building blocks.

These conditions led to the formation of anisotropic ribbonlike structures in the simulation (Fig. 1E, fig. S4, and movie S1). As observed in the experiments (Fig. 1B and figs. S1 and S2), nanoribbon growth was self-limiting in the x and y directions owing to the balance of competing forces at different length scales, leading to the robust formation of uniform structures of comparable size. Additionally, modifying the length of (His)*n* in the computational model allowed for the prediction of nanoribbon morphologies. Simulations predicted thicker nanoribbons with a 6-mer of histidine (His6) and thinner structures with a 33-mer of histidine (His33), compared with His11 (table S2, fig. S5, and movies S2 and S3). These were consistent with transmission electron microscopy (TEM) images of CDA/Mn/His6 and CDA/Mn/His33, indicating that (His)*n* plays a key role in regulating nanoassembly formation through electrostatic repulsion. Overall, the computational model accurately predicted the self-assembly behavior of CDA/Mn/(His)*n*, highlighting the potential for the rational design of tunable nanostructures.

To enable systemic delivery, we encapsulated CDA/Mn/His11 within lipid layers using His11 conjugated with 1,2-dioleoyl-*sn*-glyce ro-3-phosphoethanolamine (DOPE), forming lipid-coated nanoparticles called CRYSTAL (crystal-like STING-activating nanoassemblies). Since lipid composition influences the in vivo performance of lipid-based nanoparticles (24, 25), we varied the lipid compositions of CRYSTAL (CRYSTAL-1 to CRYSTAL-6) and evaluated antitumor efficacy in vivo (fig. S6A). Each formulation exhibited distinct surface charges depending on its lipid composition (fig. S6B). Compared with other formulations, CRYSTAL-1 (DOPC:Chol:DP-PEG5K:14PA = 1:1:0.07:0.3) given intravenously showed superior antitumor efficacy with favorable liver function profiles in B16F10 tumor–bearing mice (fig. S6C).

We compared CRYSTAL-1 and CRYSTAL-5 (DOPC:Chol:DP-PEG5K = 1:1:0.07) in more detail (fig. S7). While CRYSTAL-1 and CRYSTAL-5 exerted comparable antitumor efficacy, CRYSTAL-1 induced much less elevation of aspartate aminotransferase (AST) and alanine aminotransferase (ALT), enzymes associated with liver inflammation, than did CRYSTAL-5 (fig. S7B). Supporting this, bulk RNA sequencing analysis revealed that CRYSTAL-5–treated livers were enriched for inflammatory and cellular stress–response pathways, including tumor necrosis factor (TNF) signaling, p53 signaling, and the nonalcoholic fatty liver disease pathway (fig. S7C). CRYSTAL-1–treated livers were enriched for canonical hepatic programs—including metabolism of vitamin B6, linoleic acid, retinol, and nitrogen as well as steroid biosynthesis—consistent with normal hepatic functions. These transcriptional signatures correlated with the better liver safety profile observed in CRYSTAL-1. Thus, we selected CRYSTAL-1 (hereafter referred to as CRYSTAL) for further investigation. Cryo–electron microscopy (cryo-EM) and fast Fourier transform (FFT) analysis revealed that CRYSTAL contained a tightly packed, highly ordered nanoassembly core (Fig. 1F). CRYSTAL retained an elongated morphology with a median aspect ratio of 3.76 and exhibited an ordered nanoscale spacing of ~1.8 nm within its core assembly (Fig. 1G and fig. S8A), consistent with the predicted structure of the CDA/Mn/His11 nanoassembly (figs. S3 and S4).

CRYSTAL enhanced cellular uptake of STING agonists via the endocytic pathway, as evidenced by colocalization of the STING agonist signal and LysoTracker, and also induced immunostimulatory activity in a STING-dependent but gasdermin D–independent manner (fig. S9). To investigate whether nanoribbon formation influenced in vivo performance, we synthesized an unordered CDA-Mn^2+^ metallonanoparticle (uCMP) without preassembling CDA, Mn^2+^, and DOPE-His11. The resulting uCMP was ~44 nm in size with a morphology typical of traditional lipid nanoparticles (fig. S8B). Both CRYSTAL and uCMP had a similar CDA-to-Mn^2+^ ratio, as determined by inductively coupled plasma mass spectrometry (ICP-MS) (fig. S8C). Notably, CRYSTAL exhibited a slower release of CDA compared with uCMP, likely because of the structural stability of the nanoassembly core (fig. S8D). This improved the in vivo pharmacokinetic profile of CRYSTAL in B16F10 tumor–bearing mice, with a more than fivefold increase in distribution half-life in comparison to uCMP (fig. S10) and a marked increase in circulation in comparison to other polymeric nanoparticle–based STING agonists (18).

We examined systemic STING activation efficiency across different dosage levels (Fig. 1, H and I) and included diamidobenzimidazole (diABZi), a widely used systemic STING agonist (13), as our benchmark. At all dose levels, CRYSTAL administered intravenously elicited a more potent STING activation and exhibited superior antitumor efficacy in B16F10 tumor–bearing mice compared with both uCMP and even a threefold higher dose of diABZi. Even at ultralow doses (0.1 or 0.5 μg), CRYSTAL induced systemic STING activation and effectively suppressed tumor growth in both the B16F10 melanoma model (Fig. 1, H and I) and the NOOC1 head and neck cancer model (fig. S11). In contrast, uCMP and diABZi induced only limited systemic STING activation with a narrow therapeutic window.

We compared the activity of CRYSTAL and diABZi across immune cell types. CRYSTAL selectively activated STING (as assessed by CXCL10 and IFN-I) in bone marrow–derived dendritic cells (BMDCs) in vitro, whereas diABZi triggered STING activation in BMDCs, CD4^+^, and CD8^+^ T cells (fig. S12A). Moreover, free soluble diABZi, CDA, and Adu-S100 induced prominent CD8^+^ T cell death across a broad concentration range in vitro, whereas CRYSTAL did not (fig. S12, B and C), likely owing to the nonphagocytic nature of CD8^+^ T cells (26). CRYSTAL intravenous treatment resulted in strong innate immune activation in the spleen, enhancing activation markers (CD80 and/or CD86) across cDC1, cDC2, macrophages, monocytes, and neutrophils (fig. S13), compared with threefold higher diABZi. In tumor tissues, CRYSTAL increased cDC1 abundance and activation, enhanced monocyte activation [major histocompatibility complex class II (MHC-II), CD86], and reduced M2-like macrophages (CD206) (fig. S13).

Three doses of CRYSTAL were well tolerated in mice, with only transient body weight changes and no differences in key blood chemistry parameters (fig. S14). Given that the colon is a primary site for immunotherapy-related autoimmune side effects (27), we examined colon histopathology in CRYSTAL-treated mice with complete tumor regression. No differences in colon length or structure were observed in comparison to healthy controls (fig. S15). The CRYSTAL platform also demonstrated broad applicability, effectively delivering various small-molecule STING agonists currently in preclinical or clinical development, including Tak-676 (14), E7766 (28), and ADU-S100 (29). Even at 2 to 4% doses of these free STING agonists, CRYSTAL achieved superior antitumor efficacy (Fig. 1J). When systemic cytokine levels were comparable between CRYSTAL and free STING agonists (fig. S16), CRYSTAL demonstrated stronger antitumor responses, suggesting enhanced on-target STING activation. In addition, mice that achieved complete tumor regression after CRYSTAL treatment were 100% resistant to tumor rechallenge, indicating the development of immune memory (Fig. 1K). Thus, CRYSTAL achieved robust antitumor efficacy with a dose-sparing effect.

### CRYSTAL regresses large tumors in mice and rabbits through targeted STING activation

**Fig. 2.**
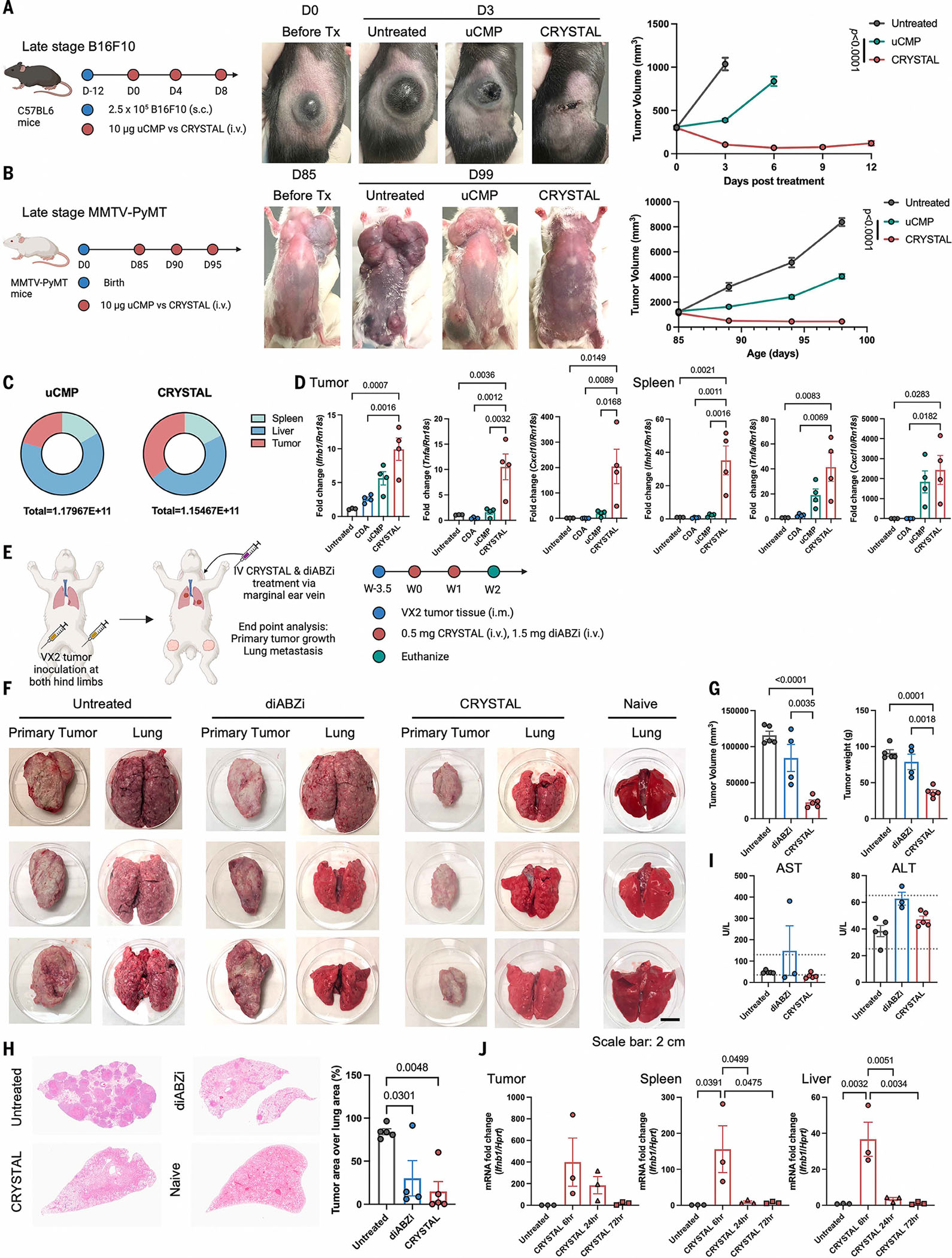
CRYSTAL regresses large tumors in mice and rabbits through targeted STING activation. (**A**) Antitumor efficacy of uCMP and CRYSTAL in late-stage B16F10 tumor–bearing C57BL/6 mice. Left to right: treatment regimen, representative tumor images before (D0) and after treatment (Tx) (D3), and tumor growth curve. (**B**) Antitumor efficacy of uCMP and CRYSTAL in late-stage spontaneous MMTV-PyMT breast cancer model. Left to right: treatment regimen, representative tumor images on D85 (before treatment) and D99, and tumor growth curve. (**C**) Biodistribution of uCMP and CRYSTAL 6 hours after intravenous administration in MMTV-PyMT mice. uCMP and CRYSTAL were labeled with Cyanine7 (Cy7) dye by mixing CDG-Cy7 with CDA during particle synthesis. (**D**) RT-PCR analysis of *Ifnb1*, *Tnfa*, and *Cxcl10* expression 6 hours after intravenous injection of 10 μg CDA, uCMP and CRYSTAL in tumor and spleen. (**E**) New Zealand white rabbits were inoculated with minced VX2 tumor tissues intramuscularly in both hindlimbs. After 3.5 weeks, rabbits received two cycles of intravenous injections of 0.5 mg CRYSTAL and 1.5 mg diABZi at 1-week intervals. One week after the second dose, rabbits were euthanized for analysis. (**F** to **J**) The therapeutic efficacy of CRYSTAL in the VX2 rabbit model. (F) Representative images of primary tumors and lungs from each treatment group (scale bar: 2 cm). Lungs from naïve rabbits were included for comparison. (G) Measurement of volume and weight of primary tumors (sum of two primary tumors in both hind limbs). (H) Representative H&E-stained lung tissues and quantification of tumor burden (% tumor area per lung section). (I) Measurement of liver enzymes (AST and ALT) at the end point. The dotted gray line indicates the normal range of each enzyme. (J) Pharmacodynamics of STING activation in rabbits. RT-PCR analysis of *Ifnb1* expression in tumor, spleen, and liver at 6, 24, and 72 hours after intravenous injection of 0.5 mg CRYSTAL in VX2 tumor–bearing rabbits. The data represent the mean ± SEM with *n* = 10 (A) and *n* = 9 (B) biologically independent samples pooled from two independent experiments. The data represent the mean with *n* = 3 (C) or the mean ± SEM with *n* = 3 or 4 (D), *n* = 4 or 5 [(G) and (H)], *n* = 3 to 5 (I), and *n* = 3 (J) biologically independent samples, with each dot representing an individual animal. The data were analyzed by one-way ANOVA [(D), (G), (H), and (J)], or two-way ANOVA [(A) and (B)] with Tukey’s multiple comparison post hoc test.

Late-stage tumors capture the complexity of the tumor microenvironment (TME) (30), and treatments capable of regressing late-stage tumors hold translational potential. In the late-stage B16F10 melanoma model (~1 cm in diameter), three cycles of uCMP intravenous therapy led to minor tumor suppression, followed by tumor relapse (Fig. 2A). Three cycles of CRYSTAL intravenous therapy resulted in rapid tumor regression and sustained tumor control (Fig. 2A). We used an even more aggressive MMTV-PyMT model of spontaneous triple-negative breast cancer and waited until multiple large tumors were present throughout the body with an average total tumor volume of 1110 mm^3^ per mouse. CRYSTAL intravenous therapy regressed large MMTV-PyMT tumors (Fig. 2B), whereas uCMP exhibited only modest tumor growth inhibition.

Upon examining their biodistribution, we found uCMP predominantly localized in the liver, whereas CRYSTAL showed tumor accumulation (Fig. 2C and fig. S17, A and B). In addition, CRYSTAL accumulated in spleen and tumor, leading to robust STING activation, as evidenced by strong up-regulation of *Ifnb1*, *Tnfa*, and *Cxcl10* in spleen and tumor (Fig. 2D). In contrast, free CDA or uCMP induced only limited STING activation. Correspondingly, CRYSTAL elevated levels of IFN-β, TNF-α, CXCL10, and interleukin-6 (IL-6) in the TME (fig. S17C), whereas free CDA and uCMP did not. At the cellular level, CRYSTAL exhibited efficient uptake among various myeloid cells, including dendritic cells, macrophages, and monocytes, both in the spleen and tumor (fig. S18).

We evaluated the therapeutic efficacy of CRYSTAL in the VX2 syngeneic tumor model in New Zealand white rabbits (31). Minced VX2 tumor tissue was injected intramuscularly into both hind limbs of each rabbit, providing a rich blood supply to support primary tumor growth and lung metastasis (Fig. 2E). This model allowed us to assess systemic CRYSTAL intravenous therapy on primary and metastatic tumor burdens. After 5.5 weeks of tumor inoculation, rabbits in the control group had massive primary tumor burden and severe lung metastases (Fig. 2, F and G). Two cycles of weekly 1.5 mg diABZi intravenous therapy showed minimal antitumor effect (Fig. 2, F and G). Two cycles of weekly 0.5 mg CRYSTAL intravenous treatment suppressed primary tumor growth (Fig. 2, F and G) and lung metastasis (Fig. 2F). Hematoxylin and eosin (H&E) staining of lung sections from untreated rabbits revealed >90% tumor burden, whereas systemic CRYSTAL treatment markedly reduced lung metastases (Fig. 2H). Additionally, CRYSTAL-treated rabbits maintained normal liver function, with AST and ALT levels remaining within the normal range (Fig. 2I).

We investigated the kinetics of STING activation in the tumor, spleen, and liver in VX2 tumor–bearing rabbits. After CRYSTAL intravenous therapy, we observed strong elevation of *Ifnb1* and *Cxcl10* transcripts in both tumor and spleen at 6 hours after treatment and a gradual decline at 24 and 72 hours (Fig. 2J and fig. S19A). While the liver displayed a similar temporal profile, the magnitude of *Ifnb1* induction at 6 hours was considerably higher in tumor (~400-fold) and spleen (~156-fold), compared with liver (~37-fold) (Fig. 2J), perhaps because of the lower expression level of STING in the liver (32, 33). H&E analysis revealed only minor inflammatory changes in the liver within 3 days of CRYSTAL treatment (fig. S19B). We concluded that the antitumor efficacy of CRYSTAL was achieved by efficient, targeted STING activation at both the organ and cellular levels, with only transient elevation of liver enzymes in late-stage tumor models in mice and rabbits.

### Host and tumor factors critical for CRYSTAL therapy

**Fig. 3.**
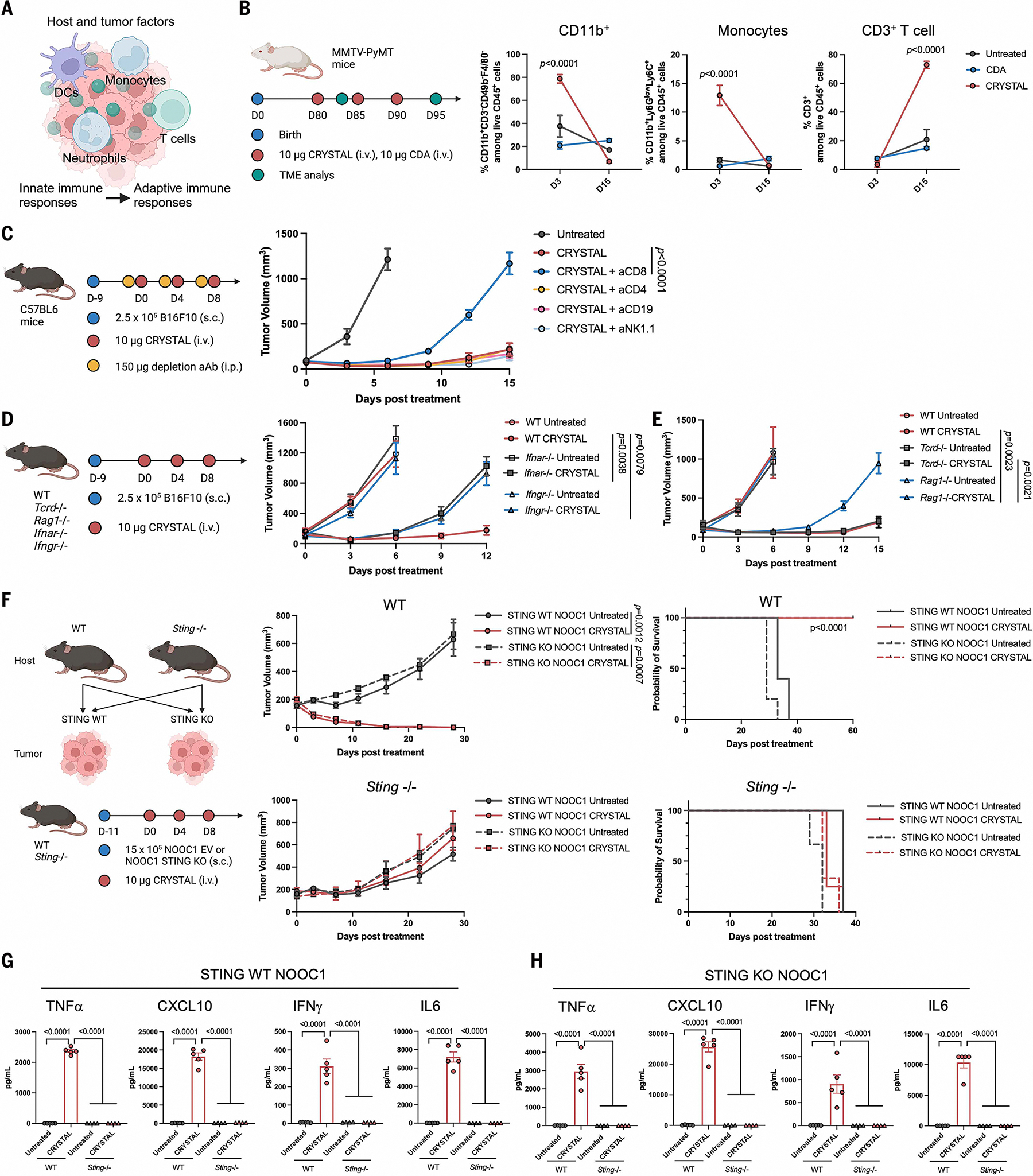
Host and tumor factors critical for CRYSTAL therapy. (**A**) Schematic shows host and tumor factors orchestrate both innate and adaptive immune responses. (**B**) MMTV-PyMT mice (D80 after birth) received intravenous injections of 10 μg CDA or CRYSTAL on D0, D5, and D10. Tumors were collected for immune profiling on D3 and D15. Changes in myeloid cells (CD11b^+^CD3^−^CD49b^−^F4/80^−^), monocytes (CD11b^+^Ly6C^+^Ly6G^low^), and T cells (CD3^+^) among live CD45^+^ cells from D3 to D15 are shown from left to right. (**C**) Antitumor efficacy of CRYSTAL after antibody-mediated depletion of various immune cells (anti-CD8, anti-CD4, anti-CD19, and anti-NK1.1) in B16F10 tumor–bearing mice. (**D** and **E**) Antitumor efficacy of CRYSTAL in B16F10 tumor–bearing WT, *Ifnar*^−/−^, *Ifngr*^−/−^ C57BL/6 mice (D) or WT, *Tcrd*^−/−^, *Rag1*^−/−^ C57BL/6 mice (E). (**F**) Antitumor efficacy of CRYSTAL in STING WT NOOC1 and STING knockout (KO) NOOC1 tumor–bearing WT C57BL/6 mice [top: tumor growth curve (left), survival curve (right)] and *Sting*^−/−^ C57BL/6 mice [bottom: tumor growth curve (left), survival curve (right)]. (**G** and **H**) Serum cytokine analysis at 6 hours after intravenous injection of CRYSTAL in STING WT NOOC1 tumor–bearing WT or *Sting*^−/−^ C57BL/6 mice (G) and in STING KO NOOC1 tumor–bearing WT or *Sting*^−/−^ C57BL/6 mice (H). The data represent the mean ± SEM with *n* = 5 (B), *n* = 3 to 5 (C), *n* = 5 (D), *n* = 4 or 5 (E), and *n* = 3 to 5 (F) biologically independent samples or the mean ± SEM with *n* = 4 or 5 [(G) and (H)] biologically independent samples, with each dot representing an individual mouse. Shown is a representative experiment from two independent experiments; the independent repeats of (F) are shown in fig. S37 and those of (C) to (E) are shown in Dryad (55). The data were analyzed by one-way ANOVA [(G) and (H)] or two-way ANOVA [(B) to (F)] with Tukey’s multiple comparison post hoc test for tumor growth curve, or log-rank (Mantel-Cox) test for survival curve.

To elucidate mechanisms driving the therapeutic efficacy of CRYSTAL, we systematically analyzed its effects on the TME and immune response (Fig. 3A). Late-stage MMTV-PyMT mice were treated intravenously with CRYSTAL or free CDA on days 80, 85, and 90, and we performed immune profiling in the TME on day 83 (D83) and D95 (3 days and 15 days after the first CRYSTAL dose, respectively) (Fig. 3B). By D3, CD11b^+^ myeloid cells, particularly monocytes (Ly6G^low^Ly6C^+^), were recruited to the TME, suggesting rapid activation of innate immune responses after systemic CRYSTAL treatment (Fig. 3B). By D15, CD3^+^ T cells became the dominant population in the TME, indicating a transition from innate to adaptive immune responses (Fig. 3B). These findings suggested that CRYSTAL orchestrated an early innate immune response, driving adaptive immunity for sustained antitumor efficacy.

To identify the immune cell populations required for CRYSTAL-mediated tumor control, we depleted specific immune subsets using antibodies during CRYSTAL therapy in the B16F10 tumor model. Depletion of CD4^+^ T cells, CD19^+^ B cells, and natural killer (NK) cells did not affect CRYSTAL-mediated tumor regression (Fig. 3C). However, depletion of CD8^+^ T cells impaired antitumor efficacy (Fig. 3C). In addition, the efficacy of CRYSTAL was reduced in *Ifnar*^−/−^ and *Ifngr*^−/−^ mice (Fig. 3D), highlighting the indispensable roles of both type I and type II IFNs in sustaining antitumor immunity (Fig. 3D). CRYSTAL exhibited reduced antitumor activity in *Rag1*^−/−^ mice, which lack mature T and B cells, reinforcing the role of adaptive immunity (Fig. 3E). CRYSTAL’s efficacy remained intact in *Tcrd*^−/−^ mice, indicating that depletion of γδ T cells alone was insufficient to abolish CRYSTAL-mediated protection (Fig. 3E). The initial efficacy (until D9) of CRYSTAL was unaffected by depletion of macrophages, monocytes, or neutrophils or in cDC1-deficient (*Batf3*^−/−^) mice (fig. S20, A and B). However, by D12, CRYSTAL’s efficacy declined after depletion of macrophages or neutrophils or in *Batf3*^−/−^ hosts, highlighting the role of myeloid cells in sustaining adaptive immunity (fig. S20, A and B). This differs from the sole dependence of cDC1s after intratumoral administration of STING agonists in the form of viruslike particles or synthetic micelles (34, 35). Blocking STING-induced cytokines altogether (i.e., TNF-α, IFN-γ, IFN-I, IL-6) abolished the efficacy of CRYSTAL (fig. S20C) (19). Prior studies have reported that STING activation in endothelial cells can contribute to the therapeutic activity of certain STING agonists (16), and we observed that CRYSTAL could engage endothelial cells in vitro (fig. S12A); nevertheless, CRYSTAL remained highly effective in a poorly vascularized pancreatic ductal adenocarcinoma model (fig. S21). We concluded that although endothelial cell involvement may occur, CRYSTAL’s efficacy lies in robust activation of STING-responsive myeloid cells that promote durable CD8^+^ T cell immunity.

We explored how activating STING within tumor cells contributed to the efficacy of CRYSTAL. In *Sting*^−/−^ mice, CRYSTAL completely lost its efficacy in the NOOC1 tumor model (Fig. 3F, bottom), regardless of STING expression in tumor cells (Fig. 3F, bottom). In contrast, complete tumor regression was observed in *Sting* wild-type (WT) hosts, even when NOOC1 tumors lacked STING expression (Fig. 3F, top). CRYSTAL triggered robust inflammatory cytokine production in WT hosts, but not in *Sting*^−/−^ hosts, regardless of tumor STING expression (Fig. 3, G and H). CRYSTAL resulted in relatively modest IFN-γ levels compared with those elicited by immunocytokines, such as IL-12 (36) and IL-18 (37), suggesting limited IFN-γ–mediated side effects from CRYSTAL. These results demonstrate that host STING expression, rather than tumor-intrinsic STING, is essential for the efficacy of CRYSTAL.

### CRYSTAL remodels the tumor and spleen microenvironment

**Fig. 4.**
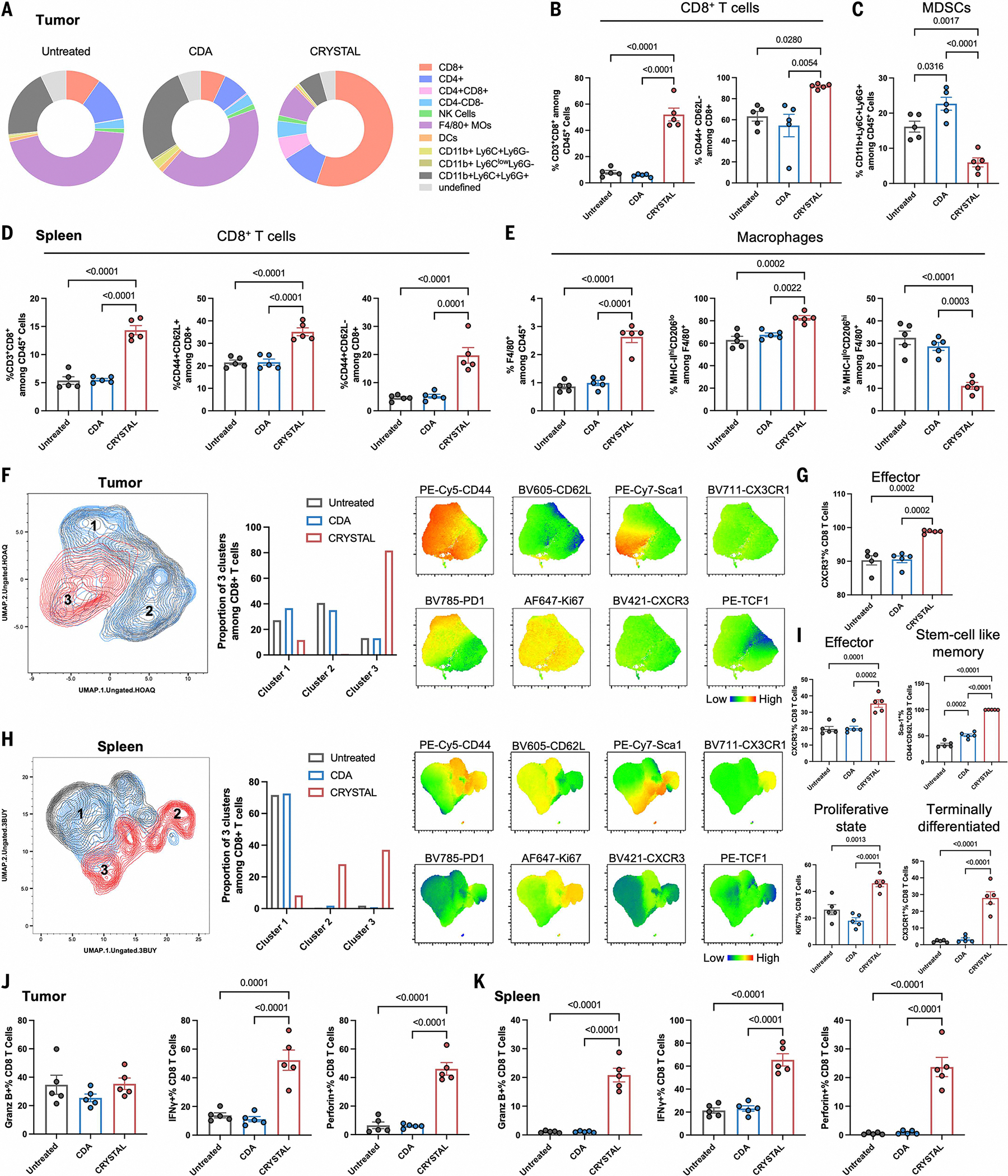
CRYSTAL remodels the tumor and spleen microenvironment. MMTV-PyMT mice (D80 after birth) were enrolled and received intravenous injections of 10 μg CDA or CRYSTAL on D0, D5, and D10. Tumors and spleens were collected for immune profiling on D15 via flow cytometry (see figs. S22, S24, and S25 for the gating strategy). (**A** to **C**) Frequency of major immune cell types among CD45^+^ live cells in the TME (A), frequency of CD3^+^CD8^+^ T cells among CD45^+^ live cells and effector memory (CD44^+^CD62L^−^) CD8 T cells in tumor (B), frequency of CD11b^+^Ly6C^+^Ly6G^+^ MDSCs among CD45^+^ live cells (C). (**D** and **E**) Frequency of CD3^+^CD8^+^ T cells among CD45^+^ live cells, effector memory (CD44^+^CD62L^−^) CD8^+^ T cells, and central memory (CD44^+^CD62L^+^) CD8^+^ T cells in spleen (D), frequency of F4/80^+^ macrophages among CD45^+^ live cells, and frequency of M1-like (MHC-II^hi^CD206^lo^) and M2-like (MHC-II^lo^CD206^hi^) macrophages in spleen (E). (**F** and **G**) Characterization of CD8^+^ T cell phenotypes in the TME. UMAP (uniform manifold approximation and projection) plots of concatenated CD8^+^ T cells from untreated, CDA, and CRYSTAL groups with proportion of each cluster and expression levels of different phenotypic markers (F). Frequency of effector-like (CXCR3^+^) CD8^+^ T cells (G). (**H** and **I**) Characterization of CD8^+^ T cells phenotypes in the spleen. UMAP plots of concatenated CD8^+^ T cells from untreated, CDA, and CRYSTAL groups with proportion of each cluster and expression levels of different phenotypic markers (H). Frequency of effector-like (CXCR3^+^) CD8^+^ T cells, stem cell–like memory Sca1^+^ T cells among CD44^−^CD62L^+^ CD8^+^ T cells, proliferative (Ki67^+^) CD8^+^ T cells, and terminally differentiated (CX3CR1^+^) CD8^+^ T cells (I). (**J** and **K**) Intracellular cytokine staining assay of CD8^+^ T cells upon PMA (phorbol 12-myristate 13-acetate) and ionomycin stimulation. Frequency of granzyme B (Granz B^+^)–, IFN-γ (IFN-γ^+^)–, and perforin (Perforin^+^)–expressing CD8^+^ T cells in tumor (J) and spleen (K). The data represent the mean (A) or the mean ± SEM with *n* = 5 [(B) to (E), (G), and (I) to (K)] biologically independent samples, with each dot representing an individual mouse. Shown is a representative experiment from two independent experiments; the independent repeats are shown in Dryad (55). The data were analyzed by one-way ANOVA, followed by Tukey’s multiple comparison post hoc test.

Within the TME, CRYSTAL promoted a ninefold increase in the frequency of CD8^+^ T cells compared with the CDA-treated control group (Fig. 4, A and B, and figs. S22 and S23). CRYSTAL also increased the frequency of effector memory CD8^+^ T cells (T_EM_ cells: CD44^+^CD62L^−^) and reduced myeloid-derived suppressor cell (MDSC) population (CD11b^+^Ly6C^+^Ly6G^+^), compared with the CDA control group (Fig. 4, A to C, and fig. S23). Similarly, in the spleen, CRYSTAL treatment resulted in a threefold increase in CD8^+^ T cell populations compared with CDA control, with notable enrichment of both central memory CD8^+^ T cells (T_CM_ cells) (CD44^+^CD62L^+^) and CD8^+^ T_EM_ cells (Fig. 4D). Additionally, CRYSTAL led to an enrichment of macrophages in the spleen, with higher frequency of M1-like macrophages (MHCII^hi^CD206^lo^) over M2-like macrophages (MHCII^lo^CD206^hi^) (Fig. 4E).

Given the substantial CD8^+^ T cell infiltration driven by CRYSTAL, we characterized their phenotypes. In tumor tissues, CRYSTAL induced distinct clusters of CD8^+^ T cells with high Sca1 expression (Fig. 4F and fig. S24) and increased CXCR3^+^ effector CD8^+^ T cells (Fig. 4G), a hallmark of IFN-I–driven immune activation (38, 39). Distinct clusters of CD8^+^ T cells were also observed in the spleen after CRYSTAL treatment (Fig. 4H). Specifically, cluster 2, which was exclusively induced by systemic CRYSTAL treatment, displayed high Ki67 expression and elevated CX3CR1 levels, characteristics of terminally differentiated CD8^+^ T cells with enhanced cytotoxicity and effector functions (40) (Fig. 4, H and I, and fig. S25). Conversely, cluster 3 exhibited high CD62L and Sca1 expression but low CD44 levels (Fig. 4, H and I), a subset of T cells recently reported as multipotent, stem cell–like memory T cells (41). Furthermore, effector CXCR3^+^CD8^+^ T cells were increased in the spleen (Fig. 4I). CRYSTAL treatment increased IFN-γ^+^CD8^+^ T cells and perforin^+^CD8^+^ T cells in tumors (Fig. 4J and fig. S26) and increased granzyme B^+^CD8^+^ T cells, IFN-γ^+^CD8^+^ T cells, and perforin^+^CD8^+^ T cells in the spleen (Fig. 4K and fig. S26). Additionally, systemic CRYSTAL treatment induced cytotoxic granzyme B^+^, IFN-γ^+^, and perforin^+^ CD4^+^ T cells and activated NK cells (fig. S27). CRYSTAL treatment slightly increased regulatory T (T_reg_) cell frequency in the spleen, but not in tumor, possibly as a counterbalance to strong CD8^+^ T cell responses. Despite this, the CD8-to-T_reg_ cell ratio increased in both spleen and tumors after CRYSTAL treatment (fig. S28). To assess the tumor specificity of CRYSTAL-induced CD8^+^ T cells, we adoptively transferred OT-I cells into mice bearing either B16F10-OVA or B16F10 tumors. CRYSTAL expanded OT-I cells only in the presence of cognate antigen (B16F10-OVA), but not in antigen-negative tumors (fig. S29, A and B), indicating CRYSTAL-induced expansion of tumor antigen–specific CD8^+^ T cells, potentially through in situ antigen release and cross-priming. We concluded that CRYSTAL treatment remodeled the immunosuppressive microenvironment of both tumor and spleen, driving the development of distinct and highly functional immune populations.

### CRYSTAL safely elicits STING activation in healthy dogs and nonhuman primates

**Fig. 5.**
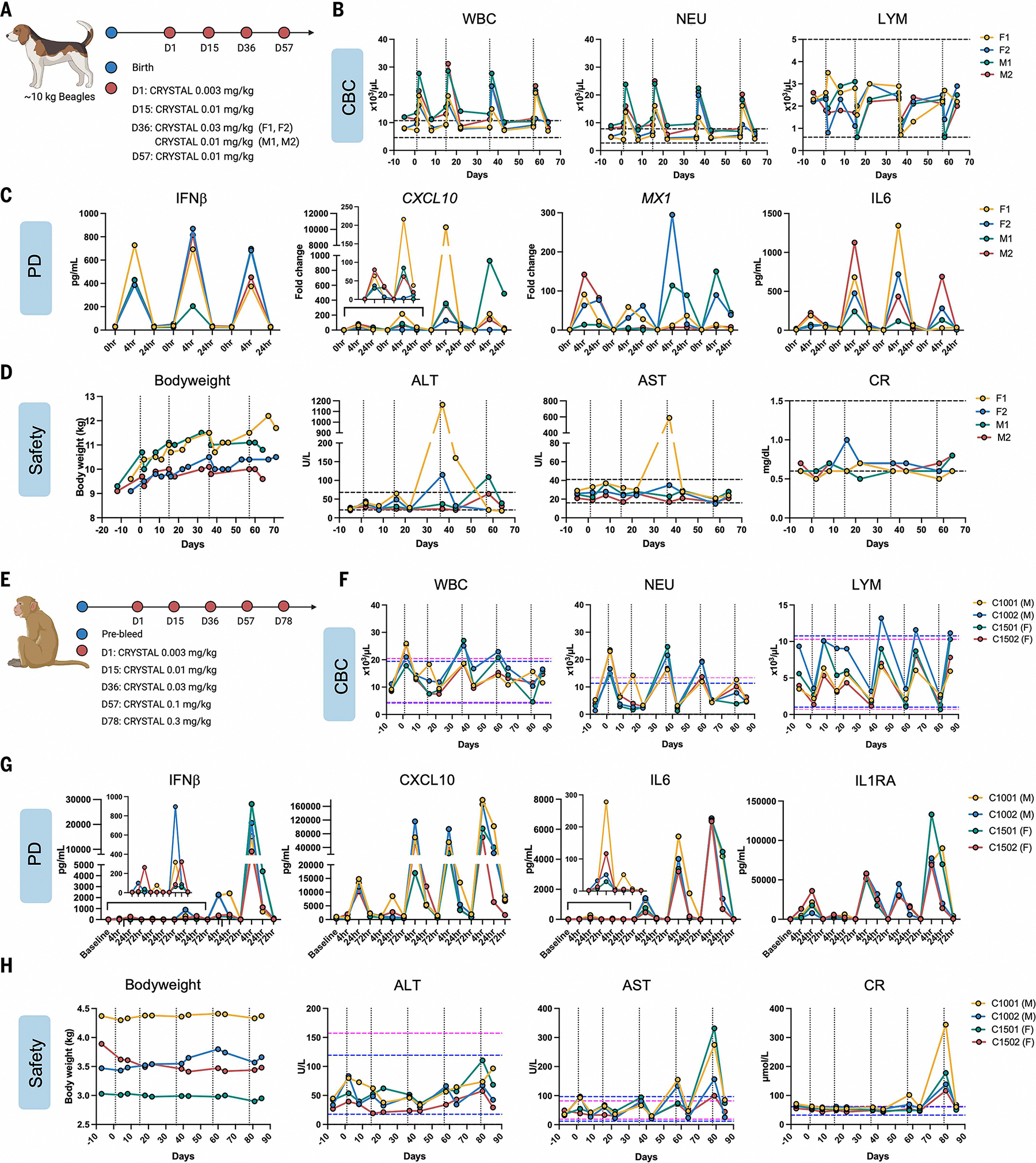
CRYSTAL safely elicits STING activation in healthy dogs and nonhuman primates. (**A** to **D**) Dose escalation trial of systemic CRYSTAL treatment in two female (F1, F2) and two male (M1, M2) naïve beagles. Dosing regimen is shown in (A). The dynamic changes of WBC, NEU, and lymphocyte (LYM) counts were measured before and after each dose (the dashed horizontal lines indicate normal ranges, dotted vertical lines indicate the day of treatment) (B). Investigation of systemic STING activation responses at 4 and 24 hours after each dose with ELISA (IFN-β and IL-6) and RT-PCR (*CXCL10* and *MX1*) [values before each dose (0 hours) set as the baseline] (C). Safety measurements of individual dogs included body weight (BW), liver enzyme ALT and AST levels, and CR level (dashed horizontal lines indicate normal ranges, and dotted vertical lines indicate the day of treatment) (D). (**E** to **H**) Dose escalation study of systemic CRYSTAL treatment in two female (C1501, C1502) and two male (C1001, C1002) cynomolgus monkeys. Dosing regimen is shown in (E). The dynamic changes in WBC, NEU, and LYM counts were measured before and after each dose (dashed blue and pink horizontal lines indicate normal ranges for male and female NHPs, respectively, and dotted vertical lines indicate the day of treatment) (F). Investigation of systemic STING activation responses at 4, 24, and 72 hours after each dose with ELISA (IFN-β) and Meso Scale Discovery (MSD) assays (CXCL10, IL-6, and IL-1RA) [values from prebleeding (D-7) set as the baseline] (G). Safety measurement of individual NHPs included BW, liver enzyme ALT and AST levels, and CR level (dashed blue and pink horizontal lines indicate normal ranges for male and female NHPs, respectively, and dotted vertical lines indicate the day of treatment) (H). Each line corresponds to an individual animal, and each dot represents a measurement obtained at a specific time point.

To evaluate the translational potential of CRYSTAL, we performed a dose escalation study in healthy beagles and nonhuman primates (NHPs), assessing dose-response relationships, STING activation, and potential dose-limiting toxicities. In healthy beagles (~10 kg), four escalating doses of CRYSTAL were administered by intravenous infusion over 30 min (Fig. 5A). At each dose, CRYSTAL transiently increased white blood cells (WBCs), particularly neutrophils (NEUs), peaking at 24 hours after each dose and returning to baseline by D7, indicating systemic innate immune activation (Fig. 5B). Even at an ultralow dose of 0.003 mg/kg, CRYSTAL induced potent STING activation, as demonstrated by elevation in serum IFN-β, IL-6, TNF-α, and IFN-γ levels, along with increased *CXCL10* and *MX1* transcript expression (Fig. 5C and fig. S30, A and B). These serum biomarkers peaked at 4 hours postinjection and returned to baseline within 24 hours. However, in two female dogs dosed at 0.03 mg/kg on D36, elevated ALT and AST levels were observed but gradually resolved. As a precaution, we adjusted the final dose to 0.01 mg/kg on D57, which resulted in only slightly elevated ALT and AST levels. The remaining two male dogs, who received 0.01 mg/kg on both D36 and D57, showed no signs of liver toxicity. By the end of the study, all dogs gained weight and exhibited normal liver and kidney function, as confirmed by comprehensive chemistry panels (Fig. 5D and fig. S30C). Overall, CRYSTAL achieved strong STING activation in healthy dogs with a well-tolerated safety profile.

We conducted a dose escalation study with CRYSTAL (0.003 mg/kg to 0.3 mg/kg) in cynomolgus monkeys (Fig. 5E). CRYSTAL increased WBCs and NEUs within 24 hours after intravenous infusion, returning to baseline by D7 (Fig. 5F). After the second dose (administered at a 2-week interval), we did not observe elevation of WBC and neutrophil counts in blood, indicating refractory response to CRYSTAL treatment (Fig. 5F). The refractory response might result from the ~3-week turnover rate of dendritic cells in NHPs (42) and/or STING degradation after activation (43, 44). Thus, we extended the subsequent dosing interval to 3 weeks. The extension of the dosing interval led to restoration of systemic STING activation, as evidenced by transient elevations of WBCs, NEUs, and neutrophil percentage (Fig. 5F and fig. S31). Consistent with our findings in dogs, ultralow doses of CRYSTAL elicited strong STING activation in NHPs. Even at 0.003 mg/kg dose, CRYSTAL increased IFN-β, IFN-α, IL-6, and interleukin-1 receptor antagonist (IL-1RA) levels in serum, along with robust CXCL10 (~12,600 pg/ml) secretion (Fig. 5G and fig. S32, A and B). We only observed inflammatory IL-1β at the highest CRYSTAL dose of 0.3 mg/kg (fig. S32B). The serum levels of IFN-β and CXCL10 exhibited a dose-dependent increase with CRYSTAL administration, whereas their muted levels after the second dose corresponded with the minimal changes in WBCs and NEUs. We concluded that, in this setting, WBC mobilization was a pharmacodynamic biomarker of systemic STING activation rather than an indicator of hematologic toxicity. This is in line with prior work reporting IFN-β–mediated transient elevation of WBCs and NEUs after viral infection (45).

Although cytokines are essential for therapeutic efficacy, cytokine release syndrome (CRS) is a critical safety concern in many forms of cancer immunotherapies. For example, IL-12 therapies are limited by IFN-γ toxicity, and CAR-T therapies are often complicated by IL-6–driven CRS (46). Peak levels of IL-6 and IFN-γ remained within grade 0 to 3 CRS thresholds (fig. S33) (47). CRYSTAL induced higher levels of IFN-α and CXCL10, key biomarkers of STING activation and potency (fig. S33). This separation of efficacy and toxicity biomarkers further supports a favorable safety profile.

Across all doses of CRYSTAL tested (0.003 mg/kg to 0.3 mg/kg), NHPs exhibited normal body weight, complete blood cell (CBC) counts, comprehensive chemistry panels, and serum C3a (complement activation) and no CRS-like symptoms (Fig. 5H and figs. S31 and 32C). At the highest dose (0.3 mg/kg), CRYSTAL caused acute (24 hours after injection) elevation of AST, creatinine (CR), creatine kinase, urea, and triglycerides, but these were reversible, returning to baseline within 7 days (Fig. 5H and fig. S34). We directly compared the peak CRYSTAL-induced lab values (AST, ALT, lactate dehydrogenase, CR) with those reported in human CRS grading scales with the CAR-T treatment (fig. S33) (47), and they fell within or below the grade 0 to 3 CRS range, indicating a favorable laboratory safety profile.

We summarized all mouse doses and their cross-species equivalents using standard body surface area allometric scaling (fig. S36A) and visualized the corresponding dose ranges between mice and NHPs, showing that the tumor-eliminating and tumor-regression doses in mice fall within or below the dose range evaluated in NHPs (fig. S36B). We also compiled all independent therapeutic efficacy studies stratified by dose, highlighting the robustness of CRYSTAL’s antitumor activity across multiple tumor models and a broad dose range (figs. S36C and S37). We propose that CRYSTAL demonstrates safe, potent, and repeatable STING activation in NHPs across a broad dose range, underscoring its strong translational potential.

### CRYSTAL induces potent STING activation in human samples

**Fig. 6.**
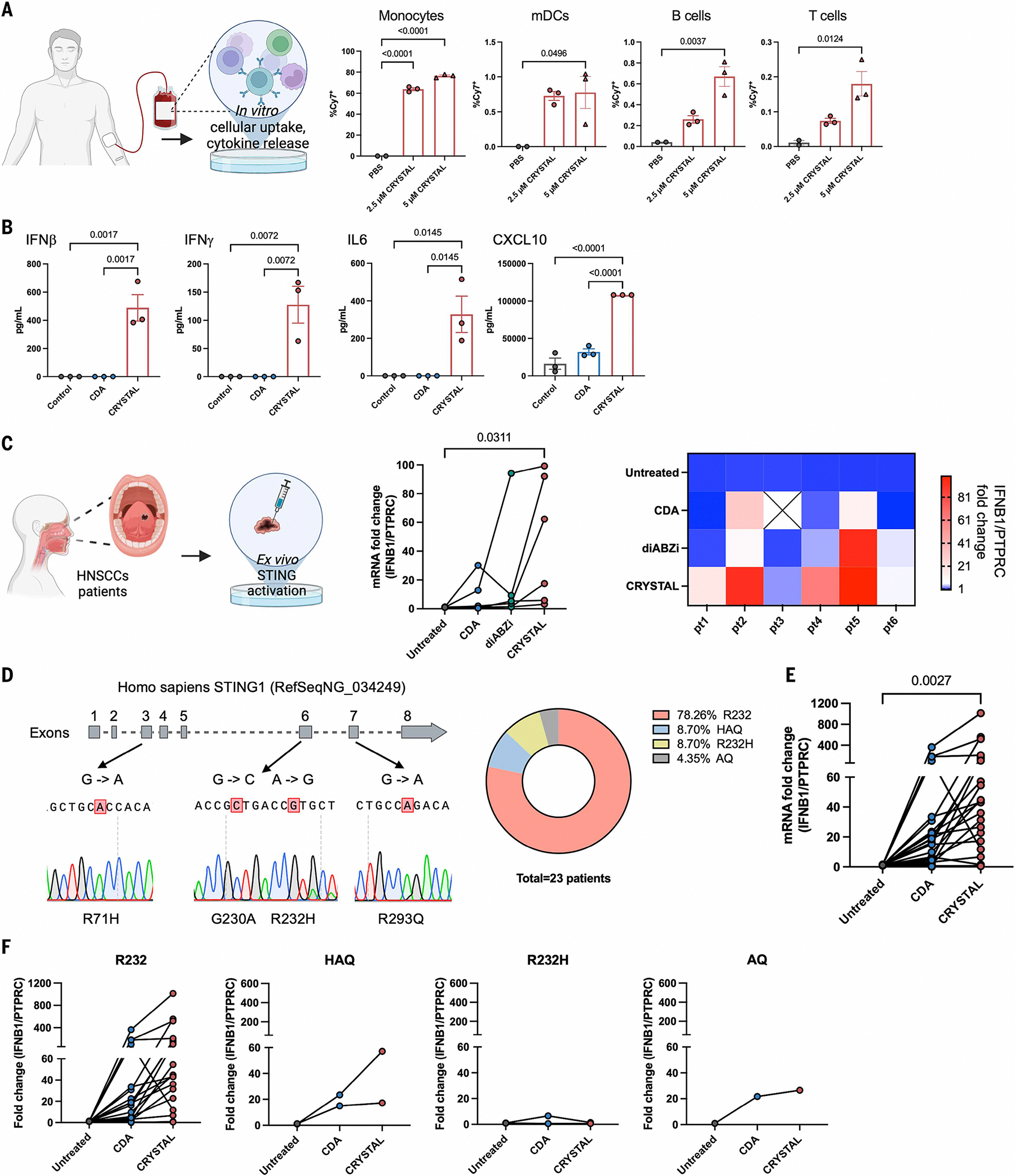
CRYSTAL induces potent STING activation in human samples. (**A** and **B**) Cellular uptake and STING activation efficiency of CRYSTAL among human PBMCs. Human PBMCs were seeded and rested overnight before treatment. PBMCs were incubated with CRYSTAL-Cy7 at 2.5 or 5 μM for 1 hour and then analyzed with flow cytometry for the frequency of CRYSTAL-Cy7^+^ cells among each cell type (A). mDCs, myeloid dendritic cells. PBMCs were cultured with 10 μg/ml CRYSTAL or free CDA overnight, followed by ELISA analysis of IFN-β, IFN-γ, IL-6, and CXCL10 (B). (**C** to **F**) STING activation by CRYSTAL in fresh human HNSCC biopsies. (C) Fresh human tumor tissues derived from six HNSCC patients were incubated with various STING agonists, and *IFNB1* expression [normalized by *PTPRC* gene (CD45)] was determined by RT-PCR (5 μg CDA and diABZi or 1 μg CRYSTAL). (D) Investigation of STING haplotypes of 23 HNSCC patient tumor tissues and percentage of each STING haplotype, including R232, HAQ, R232H, and AQ. STING haplotypes were determined by sequencing of exons 3, 6, and 7 to identify R71H (exon 3), G230A, R232H (exon 6), and R293Q (exon 7) mutations. Fresh human tumor tissues derived from these 23 HNSCC patients were incubated with 5 μg CDA or CRYSTAL, and *IFNB1* expression [normalized by *PTPRC* gene (CD45)] was determined by RT-PCR (E). *IFNB1* expression levels were plotted for various STING haplotypes (F). The data represent the mean ± SEM, with individual points showing *n* = 2 or 3 technical replicates [(A) and (B)] or biologically independent samples *n* = 6 for (C) and *n* = 23 for (E). Each line corresponds to an individual patient’s sample, and each dot represents a measurement. The data were analyzed by one-way ANOVA, followed by Tukey’s multiple comparison post hoc test.

We also investigated how CRYSTAL interacts with human immune cells. We first incubated CRYSTAL with peripheral blood mononuclear cells (PBMCs) from healthy human subjects. CRYSTAL was taken up by monocytes and to some degree by myeloid DCs, B cells, and T cells (Fig. 6A and fig. S35, A and B). CRYSTAL treatment of human PBMCs led to a 488-, 128-, 328-, and 3-fold enhanced secretion of IFN-β, IFN-γ, IL-6, and CXCL10, respectively, compared with free CDA (Fig. 6B). We also examined STING activation using fresh tumor biopsies derived from head and neck squamous cell carcinoma (HNSCC) patients undergoing tumor resection surgery. Ex vivo treatment with CRYSTAL increased IFN-I production from human tumor tissues in comparison to free CDA or diABZi treatment (Fig. 6C). Certain tissue samples (e.g., from patient 3) did not respond to CRYSTAL or other STING agonists (Fig. 6C), suggesting that other factors, such as STING haplotypes, could underlie nonresponsiveness and heterogeneity in IFN-I responses (48). We performed STING haplotype sequencing, identifying 78.3% R232 (WT), 8.7% HAQ, 8.7% R232H, and 4.3% AQ STING variants among 23 patient samples (Fig. 6D and fig. S35C), consistent with the known STING haplotype frequencies in the human population (48). Compared with free CDA, CRYSTAL treatment enhanced *IFNB1* activation, resulting in *IFNB1* elevation in 19 out of 23 patient samples examined (Fig. 6E). Among the 18 patient samples with the WT (R232) STING haplotype, all but two responded to CRYSTAL treatment (Fig. 6F). Additionally, two patients with the HAQ STING haplotype and one with the AQ STING haplotype exhibited *IFNB1* elevation after CRYSTAL treatment. However, both patients carrying the R232H STING variant failed to respond to CRYSTAL treatment (Fig. 6F).

## Discussion

Therapeutic targeting of the cGAS-STING pathway has been challenging, in part because uncontrolled STING activation can compromise efficacy while increasing the risk of systemic toxicity (12). This challenge is further compounded by the fact that STING signaling is highly cell type dependent: Activation in myeloid cells can induce innate activation and prime adaptive immunity, whereas activation in other compartments (such as CD8^+^ T cells) can be detrimental by promoting apoptosis. Within this framework, our results show that CRYSTAL preferentially elicits robust innate activation while sparing CD8^+^ T cells from apoptosis, thereby offering a mechanistic basis for its improved therapeutic index.

CRYSTAL may also address a major translational bottleneck for STING agonists: tumor-intrinsic STING dysfunction. Because STING signaling is frequently dysregulated within tumor cells across many human cancers (49), an approach that relies on host STING activation rather than tumor cell–intrinsic STING signaling may broaden the range of patients and tumor types that can benefit from treatment. This host-dependent mechanism further motivates biomarker identification that could inform patient selection to maximize clinical outcomes. A clinically consequential variable is human STING haplotype, as genetic variants can shape STING responsiveness and thereby influence both efficacy and safety. Although the molecular basis for the reduced CRYSTAL responsiveness observed in samples harboring the R232H haplotype is beyond the scope of this study, our analyses of human tumor tissues underscore the translational importance of incorporating haplotype-informed patient stratification into future clinical development strategies.

Safety remains a central concern for many forms of cancer immunotherapy. CRYSTAL’s transient and reversible cytokine response may underlie its favorable safety profile, in contrast to therapies such as CAR-T cells or immunocytokines, which can induce sustained cytokine elevations and consequently increase the risk of adverse events (46, 50). In this context, the kinetics of cytokine induction and resolution may serve as a clinically actionable parameter, informing dosage selection, regimen designs, and management of CRS.

Finally, our work highlights a broader design principle in engineering structurally ordered metalloimmunotherapy (51). By selecting different ligands, metal ions, and therapeutics, this modular platform may enable the rational design of metalloimmunotherapies with tunable secondary structures and programmable immune activation profiles, thereby addressing drug delivery and immunomodulatory challenges.

## Materials and methods

### Synthesis and characterization of CDA/His11 and CDA/Mn/His11 nanoassemblies

To prepare CDA/His11 nanoassemblies, CDA (MedChemExpress, HY-12326A) [1 mg/ml, methanol (MeOH)] and His11 (GenScript) [10 mg/ml, ethanol (EtOH)] were mixed together with 100:40 volume ratio, followed by vortexing at room temperature for 2 hours. The self-assembling condition was kept at pH < 5 to protonate His11. After water bath sonication (37 Hz, 100 W) to clear suspension, 10 μl of suspensions containing CDA/His11 was added onto TEM grid (Electron Microscopy Sciences, CF400-CU-UL) and air-dried for TEM analysis.

To form CDA/Mn/His11 nanoassembly, CDA (1 mg/ml, MeOH) and His11 (10 mg/ml) (100:40 volume ratio) were rapidly mixed together to form the first nanoassembly. The self-assembling condition was kept at pH < 5 to protonate His11. After complete self-assembling, MnCl_2_ (100 mM, MeOH, 100:40:7 volume ratio) was added rapidly with vortexing to form the intermetallic nanoassembly. CDA/Mn/His11 was vortexed at room temperature for 2 hours with sporadic water bath (37 Hz, 100 W) sonication. After centrifugation at 20,000*g* for 10 min, pellets were resuspended in deionized (DI) water with water bath sonication. Ten microliters of suspension containing CDA/Mn/His11 was added onto a TEM grid and air-dried for TEM and STEM-EDX analysis. For AFM analysis, 10 μl of suspension containing CDA/Mn/His11 was added onto a Mica disc (Fisher Scientific, NC1535937).

TEM was performed on Thermo Fisher Talos F200X G2 S/TEM with TEM mode for morphology observations and STEM mode for EDX analysis. To reduce beam damage and carbon accumulation, STEM-EDX analysis of CDA/Mn/His11 nanoassemblies was taken at cryogenic temperature (<128 K). For other TEM analysis, images were taken at room temperature (300 K). TEM image analysis was performed on DigitalMicrograph. AFM was performed on Veeco Dimension Icon Atomic Force Microscope with SCANASYSTAIR probe (Bruker) using PeakForce Quantitative Nanomechanical Mapping mode. AFM image analysis was performed on NanoScope Analysis 2.0. Zeta potential of nanoassemblies was measured with Zetasizer (Nano ZSP, Malvern, UK). FTIR spectroscopy of freeze-dried nanoassemblies was performed on IR-TRACER100 (Shimadzu).

### Synthesis and characterization of CRYSTAL and uCMP

CRYSTAL was prepared by first forming CDA/Mn/His11 nanoassemblies and then encapsulating the nanoassemblies within lipid layers. To form CDA/Mn/His11 nanoassembly, CDA (1 mg/ml, MeOH) and DOPE-His11 [10 mg/ml, EtOH with 0.5% trifluoroacetic acid (TFA)] (100:40 volume ratio) were rapidly mixed together to form the first nanoassembly. Addition of 0.5% TFA kept the histidine protonated, thus providing binding sites and electrostatic repulsion to stabilize the nanoassembly (pH < 5). After complete self-assembling, MnCl_2_ (100 mM, MeOH, 100:40:7 volume ratio) was added rapidly with vortexing to form the intermetallic nanoassembly. CDA/Mn/His11 was vortexed at room temperature for 2 hours with sporadic water bath (37 Hz, 100 W) sonication. After centrifugation at 20,000*g* for 10 min, the intermetallic nanoassembly (containing 100 μg CDA) was resuspended in lipid mixtures containing 20 μl DOPC (Avanti Polar Lipids: 850375C-25mg, 10 mg/ml in EtOH), 10 μl cholesterol (Avanti Polar Lipids: 700000P-500mg, 10 mg/ml in EtOH), and 12.8 μl 18:0 PEG5000 PE (Avanti Polar Lipids: 880220P-25mg, 10 mg/ml in EtOH). After brief water bath sonication (37 Hz, 100 W), 128 μl H_2_O was added to clear CDA/Mn/His11@lipid mixture for CRYSTAL formation. For CRYSTAL-6, DOPC was replaced with DOTAP (Avanti Polar Lipids: 890890P-200mg). The solvent and unloaded drugs were removed by dialysis against 10% sucrose overnight. To make CRYSTALs (CRYSTAL-1, -2, -3, and -4) with different lipid compositions, the additional lipid was added at the indicated amount during the dialysis, including 14PA (Avanti Polar Lipids: 830845P-200mg), 16PA (Avanti Polar Lipids: 830855P-200 mg), DOPG (Avanti Polar Lipids: 840475C-25mg), and DOTAP (Avanti Polar Lipids: 890890P-200mg). After overnight dialysis, CRYSTALs were further purified by ultrafiltration (100 kDa, Sigma Aldrich, no. UFC810008) and resuspended in 10% sucrose for further investigation. To make CRYSTAL with other STING agonists, CDA was replaced with Tak-676 (MedChemExpress, HY-148029), E7766 (MedChemExpress, HY-111999A), and ADU-S100 (MedChemExpress, HY-12885A), following the same synthesis protocol.

uCMP was prepared by rapid mixing of CDA in water phase and the lipid mixtures in ethanol, as adapted from the mRNA liquid nanoparticle fabrication method (24). The lipid mixture contained 40 μl DOPE-His11 (10 mg/ml, EtOH with 0.5% TFA), 20 μl DOPC (10 mg/ml in EtOH), 10 μl cholesterol (10 mg/ml in EtOH), and 12.8 μl 18:0 PEG5000 PE (10 mg/ml in EtOH). The aqueous phase contained 100 μg CDA (in 248 μl DI water). After rapid mixing of two phases, 7 μl MnCl_2_ (100 mM in water) was added, and the solution was dialyzed against 10% sucrose overnight, during which 26.4 μl 14PA (2.5 mg/ml, 25% THF containing DI) was added. After overnight dialysis, uCMP was further purified by ultrafiltration (100 kDa) and resuspended in 10% sucrose for further investigation.

DOPE-His11 was prepared, as previously reported (20). Briefly, DOPE-NHS (NOF America, COATSOME FE-8181SU5) [100 mg, 2 ml *N*,*N*′-dimethylformamide (DMF)] and His11 (300 mg, 2 ml DMF) were mixed at room temperature. One hundred microliters of 10% triethylamine (DMF) was added to the reaction every 12 hours three times. After 24 hours, unconjugated reactants were removed by dialysis against water (molecular weight cut-off: 2 kDa) for 2 days. The purified DOPE-His11 powder was obtained by freeze-drying.

The concentration of CDA was measured with ultraviolet absorbance at 260 nm and validated by ultra-performance liquid chromatography. The size distribution and surface charge of CRYSTAL and uCMP were measured by Zetasizer (Nano ZSP, Malvern, UK). Cryo-EM (Talos Arctica) was used to characterize the morphology of CRYSTAL and uCMP. CRYSTAL and uCMP were resuspended in 3% trehalose for the preparation of cryo-EM samples (Vitrobot). The Vitrobot setting used was 4°C, 100% humidity with 5-s blotting time, zero blotting force, and 10-s wait time.

### In vitro investigation of CRYSTAL

BMDCs were generated by harvesting bone marrow from C57BL/6, Gsdmd^−/−^, and Sting^−/−^ mice and culturing in bacteriological Petri dishes with granulocyte-macrophage colony-stimulating factor–containing medium. Media was refreshed on days 3, 6, and 8, and BMDCs were collected on day 8 for experiments. For other immune cell populations, B cells, CD4^+^ T cells, and CD8^+^ T cells were isolated from C57BL/6 spleens using isolation kits (Stemcell). Whole splenocytes were isolated from C57BL/6 spleens for bulk analysis. For cytokine assays, cells (1 × 10^5^ per well) were seeded in 96-well plates and incubated with indicated agents. After overnight culture, supernatants were collected for cytokine enzyme-linked immunosorbent assay (ELISA) at the Cancer Center Immunology Core, University of Michigan. For the cellular cytotoxicity assay, CellTox Green Cytotoxicity Assay (Promega) was used followed by the manufacturer’s instructions. For cellular uptake study, BMDCs were incubated with free CDG-Dy547 or CDG-Dy547 encapsulated in CRYSTAL for 4 hours and then co-stained with 4′,6-diamidino-2-phenylindole and Lysotracker Green for confocal imaging (Nikon A1SI).

### Description of Monte Carlo lattice growth simulations

We developed a phenomenological coarse-grained molecular model and performed lattice growth MC simulations to investigate the electrostatically controlled growth of the uniform CDA/Mn/His11 nanoribbons observed in the experiments (7) via a custom python script. In developing the model, we assumed that CDA binds to His11 and that neighboring His11/CDA complexes associate through Mn bridges (52, 53), as illustrated in fig. S3A. We further assumed that each His11 binds to a variable number of CDA. Together, these two assumptions led to the periodicity in one direction (the short axis of the nanoribbons) but not the other (the long axis). On the basis of these assumptions, the basic building block in the model comprises a single His11 oligomer oriented along the z axis and coordinated with a variable number of CDA/Mn complexes, each protruding out along the x direction (fig. S3A). We set the x,y, and z dimensions of the building block on the basis of a 3D molecular model of the CDA/Mn/His11 complex (fig. S3A). The lattice growth simulations were performed on an orthorhombic lattice with lattice vectors set by the x, y, and z dimensions of the building block.

We considered two flavors of energetic interactions, long-range electrostatic repulsion and short-range, direction-dependent attraction. Given that His11 is fully protonated at the experimental conditions, each building block contains a charge of +11*e* centered on the building block. We model the charge-charge repulsion as a Coulombic interaction with a $k_{\text{medium}}$ chosen to phenomenologically match fiber growth for His11. We assume that the energy of growth along the z direction is in the limit of irreversible binding, consistent with the uniform fiber growth observed experimentally. Growth in the y direction has an associated change in energy of a few $k_{\text{B}}T$, consistent with histidine-histidine potential of mean force calculations (54). Growth in the x direction depends on the number of CDA bound to the incoming building block (a number chosen on the fly for each attempted insertion in the simulation; see below), with a constant binding energy per CDA. Each incoming building block has at least one CDA bound to it, as our model assumes that the CDA-His11 interaction is crucial to crystal growth. We assumed that CDA cannot saturate each “side” (+x or −x) of His11, and we therefore set the maximum number CDA per side of His11 to four. Therefore, when attempting to add a building block to the growing crystal in the x direction, we randomly select one, two, three, or four CDA with equal probability that is bound to it, which in turn determines the associated change in energy.

With the interactions in the model as described above, the lattice growth simulations proceeded as follows. We randomly select a building block on the surface of the growing crystal and an open surface direction (±x,±y, or ±z) and as a trial addition add a new building block displaced one lattice vector along the growth direction from the surface particle. If the growth direction is ±x, we also choose $N_{\text{CDA-Mn}}$ as a random integer from [1, 4], distributed uniformly. The associated change in energy ΔE is the sum of the electrostatic repulsion $E_{\text{electrostatic}}$ and the direction-dependent attractions $E_{\text{growth}}$

$$\Delta E=E_{\text{electrostatic}}+E_{\text{growth}}$$

where

$$E_{\text{electrostatic}}=Q^{2}k_{\text{medium}}\sum_{i=1}^{N}r_{i}^{-1}$$

with $Q^{2}$ the charge on each particle, $k_{\text{medium}}$ the constant that is a function of the dielectric constant, and $r_{i}$ the distances between the incoming building block and the other building blocks in the cluster. The sum over i runs over all the building blocks in the cluster at the beginning of the trial move. The attractive interactions are given by $E_{\text{growth}}=N_{\text{CDA-Mn}}E_{\text{CDA-Mn}}$ for growth along ±x, $E_{\text{growth}}=E_{y}$ for growth along ±y, and $E_{\text{growth}}=E_{z}$ for growth along ±z. The trial addition is accepted with probability $p_{\text{acc}}$ according to the Metropolis criterion

$$p_{acc}=min\left(1,e^{-\Delta E/kT}\right)$$

For each trial addition, we also performed one trial removal. The removal moves consist of selecting a building block on the surface of the crystal (i.e., one with at least one exposed surface) and computing the change in energy associated with its removal using the expression for ΔE above. Note that for removal moves, the electrostatic contribution to ΔE is negative (favorable), whereas the other contributions are positive (unfavorable). The trial removal is also accepted according to the Metropolis criterion.

We ran each simulation until the growth in the x and y directions stopped, and the nanoribbons were only growing along ±z, typically 2 × 10^6^ MC sweeps, where each sweep consisted of one trial addition and one trial removal.

In addition to the CDA/Mn/His11 building blocks, we also simulated building blocks representing CDA/Mn/His6 and CDA/Mn/His33 to probe the effect of the size and charge of the polyhistidine component on the resulting crystal morphology (“Effect of His charge on crystal morphology” below). We changed the charge Q to match the number on His. Additionally, we scaled $E_{y},\;d_{z}$, and the maximum $N_{CDA-Mn}$ linearly on the basis of the values used for His11 baseline [$max\;(N_{CDA-Mn})\;=\;2$ for His6 and 12 for His33].

We characterized the self-assembled nanoribbons by their x and y dimensions. To account for irregularities in the surface of the ribbons, we took slices of the ribbon along z and averaged the x(y) across these slices to obtain the average x(y) thickness for the nanoribbon. For ribbons that “split” during growth (CDA/Mn/His33, see “Effect of His charge on crystal morphology” below), we consider each branch of the split separately; that is, the reported x and y dimensions represent the average x and y dimensions of contiguous regions of the nanoribbons.

### Effect of charge on crystal morphology

The crystal morphologies formed in the CDA/Mn/His6, CDA/Mn/His11, and CDA/Mn/His33 simulations are summarized in table S2 (dimensions reported at mean ± standard error of the mean over three replicates). We observed continuous growth along the z direction in all cases. However, the charge of the His component affected the growth in the self-limited x and y directions, with smaller x and y dimensions when increasing His length. The nanoribbon in the CDA/Mn/His33 system split into two smaller ribbons in some regions because of the relatively large electrostatic repulsion compared with the His6 and His11 systems. These results agree with experiments, which also show decreasing width and thickness of the nanoribbons with increasing His length and more irregular crystals with His33 (fig. S5).

### Investigation of CRYSTAL in mice

#### Murine tumor models:

All animal procedures were conducted following ethical guidelines and were in accordance with and approved by the Institutional Animal Care and Use Committee (IACUC) at the University of Michigan, Ann Arbor. Mice were housed in a specific pathogen–free facility on a 12 hours light-dark cycle at 20° to 26°C with 30 to 70% humidity, in ventilated cages with standard bedding and environmental enrichment. Animals were group-housed (three to five mice per cage) and provided ad libitum access to standard chow and water. Animals were euthanized in accordance with institutional and NIH guidelines using CO_2_ inhalation followed by cervical dislocation as a secondary physical method to ensure death. The sample size for tumor experiments was determined a priori on the basis of the effect size and variability observed in related pilot experiments, and the calculations are listed in table S3. For late-stage B16F10 murine tumor model, 6- to 8-week-old female C57BL6 mice (Jackson Laboratories or laboratorybred) were inoculated subcutaneously with the indicated number of B16F10 tumor cells [ in 100 μl Hanks’ balanced salt solution (HBSS)] on the right flank. For the late-stage spontaneous breast cancer model, MMTV-PyMT mice with an age of ~D85 were enrolled. For the B16F10 tumor model, C57BL/6 (WT), *Sting*^−/−^, *Ifnar*^−/−^, *Ifngr*^−/−^, *Tcrd*^−/−^, *Rag1*^−/−^, *Batf3*^−/−^ mice (breeding pairs purchased at Jackson Laboratories) were inoculated subcutaneously with the indicated number of B16F10 cells (in 100 μl HBSS) on the right flank. For the B16F10-OVA tumor model, C57BL/6 were inoculated subcutaneously with the indicated number of B16F10-OVA cells (in 100 μl HBSS) on the right flank. For NOOC1 tumor models, C57BL/6 and *Sting*^−/−^ mice were inoculated with the indicated number of STING WT NOOC1 or STING KO NOOC1 cancer cells [ in 100 μl 1:1 phosphate-buffered saline (PBS):Matrigel mixture] on the right flank. Tumor size and survival were monitored every 3 or 4 days. Tumor size was calculated with the equation volume = length × width^2^ × 0.5, and tumor-bearing mice were assigned to different treatment groups. For cancer cell culture, B16F10 cells were cultured in complete RPMI 1640 [10% fetal bovine serum (FBS), 1% penicillin-streptomycin (Pen/Strep)]. NOOC1 cells were cultured in MOC media. For preparation of the 1 liter MOC media, 626 ml Iscove’s modified Dulbecco’s medium, 313 ml F12 nutrient mixture, 50 ml FBS, 10 ml Pen/Strep, 1.25 ml 4 mg/ml insulin, 200 μl 200 μg/ml hydrocortisone, and 50 μl 100 μg/ml epidermal growth factor were mixed. NOOC1 cells are available through Kerafast Inc. (Cat.No. EMU061). To evaluate the safety profile of CRYSTAL, B16F10 tumor–bearing C57BL/6 mice were treated with CRYSTAL, and serum was collected at the indicated time points for chemistry panel analysis at ULAM pathology core of the University of Michigan. For orthotopic pancreatic cancer model, FVB/N mice (Jackson Laboratories) were inoculated with indicated number of Pan65671 cells (in 20 μl 1:1 PBS:Matrigel mixture) directly into the pancreas. After 14 days, the mice were euthanized to evaluate tumor burden in pancreas.

#### Cell and cytokine depletion studies:

For cell depletion study, 150 μg anti-CD8a (clone 2.43, Bioxcell), anti-CD4 (clone GK1.5, Bioxcell), anti-CD19 (clone 1D3, Bioxcell), anti-NK1.1 (clone PK136, Bioxcell), anti-Ly6C (clone Monts 1, Bioxcell), anti-Ly6G (clone 1A8, Bioxcell), and anti-CSF1R (clone AFS98, Bioxcell) were administered intraperitoneally with the indicated regimen. For cytokine depletion study, 150 μg anti-TNFa (clone XT3.11, Bioxcell), anti-IL6 (clone MP5–20F3, Bioxcell), anti-IFN-γ (clone XMG1.2, Bioxcell), anti-IFNAR (clone MAR1–5A3, Bioxcell), and anti-IL10 (clone 1D3, Bioxcell) were administered intraperitoneally with the indicated regimen.

#### Biodistribution and cellular uptake:

To analyze the in vivo biodistribution of STING agonist, 2′-[DY-547]-AHC-c-diGMP (Biolog, D 116–001) or c[3′-[sCya7]-AHC-G(2′,5′)pA(3′,5′)p] (Biolog, C 240–001) was admixed with CDA (1:20, w/w) to prepare Dy547-labeled CRYSTAL/uCMP or Cy7-labeled CRYSTAL/uCMP, following the same synthesis procedure as used for CRYSTAL and uCMP as described above. Loading of Dy547 and Cy7 was quantified by fluorescence intensity. To quantify the biodistribution of STING agonists, c[3′-[sCya7]-AHC-G(2′,5′)pA(3′,5′)p] in free form, CRYSTAL, or uCMP were injected intravenously. Mice were euthanized at 6 hours after injection, and the fluorescence intensity in the major organs was measured with IVIS. For the cellular uptake study, 2′-[DY-547]-AHC-c-diGMP in free form, CRYSTAL, or uCMP were injected intravenously. Mice were euthanized at 6 hours after injection, and the efficiency of cellular uptake among various immune subsets was investigated by flow cytometry.

#### Pharmacodynamics:

To analyze the systemic STING activation, various STING agonists were injected intravenously at the indicated doses. The cytokine levels in serum were measured by ELISA in the Cancer Center Immunology Core of the University of Michigan. For measuring the cytokines in the tumor, tumor tissues were collected at the indicated time points and weighed. Tumor homogenates were obtained by tissue dissociator (Miltenyi Biotec) and centrifuged to remove debris and precipitates. The supernatants were collected for ELISA analysis and were normalized by the tumor weight. For reverse transcription polymerase chain reaction (RT-PCR) analysis, tumor and spleen tissues were collected at the indicated time points. Tissues were cut into small pieces and preserved in RNAlater (Fisher Scientific, AM7021) until RNA extraction with RNeasy Plus Mini Kit (Qiagen, 74134), following the manufacturer’s instructions. Extracted RNA was quantified by Nanodrop (Thermo Scientific), and cDNA was obtained with PrimeScript RT Reagent Kit (Takara Bio, RR037A), following the manufacturer’s instructions. RT-PCR assay was performed on QuantStudio 5 using TaqMan Gene Expression Assay (FAM). TaqMan primers were purchased from Thermo Fisher Scientific: *Rn18s* (Mm04277571_s1), *Ifnb1* (Mm00439552_s1), *Tnfa* (Mm00443258_m1), and *Cxcl10* (Mm00445235_m1).

#### Pharmacokinetics:

To analyze pharmacokinetic profiles, CRYSTAL and uCMP were administered intravenously at the indicated doses, and plasma samples were collected at specified time points. CDA concentrations were quantified by liquid chromatography–tandem mass spectrometry (LC–MS/MS) at the PK Core of the University of Michigan. Briefly, plasma (50 μl) was mixed with 50 μl of calibration standards or water in a 96-well plate. Protein precipitation was performed by adding 250 μl of ice-cold acetonitrile containing the internal standard (AICAR-^13^C_2_^15^N, 10 ng/ml), followed by vortexing (1000 rpm, 5 min) and incubation on ice (10 min). Samples were then centrifuged (4000 rpm, 10 min, 4°C), and the supernatant (~250 μl) was transferred to a fresh plate and evaporated at 35°C. Dried samples were reconstituted in 100 μl of water, vortexed (1500 rpm, 10 min), and centrifuged again (4000 rpm, 10 min, 4°C). A 15 μl aliquot was injected for LC–MS/MS analysis.

#### OT-I adoptive transfer study:

OT-I CD8^+^ T cells were isolated from the spleens of OT-I mice using a CD8^+^ T cell isolation kit (Stemcell). Freshly isolated OT-I CD8^+^ T cells were intravenously transferred into B16F10 or B16F10-OVA tumor–bearing mice on D0. On D1, CRYSTAL was administered intravenously. Expansion of transferred OT-I CD8^+^ T cells was identified in blood (D4), spleen (D6), and tumors (D6) by Thy1.1 staining.

#### In vivo immune profiling in mice:

For immune profiling of TME and spleen, MMTV-PyMT mice with age of ~D80 or B16F10 tumor–bearing mice were enrolled. Tumor and spleen tissues were harvested for immune profiling with Spectra Flow cytometer (Cytek Aurora). For processing tumor samples, tumor tissues were cut into small pieces, dissociated into single-cell suspension with a tissue dissociator (Miltenyi Biotec). Single-cell suspension was then filtered through a 70 μm strainer and washed with fluorescence-activated cell sorting (FACS) buffer before antibody staining. For processing spleen samples, spleens were dissociated into single-cell suspension by mashing spleens with syringe plunger and filtered through 70 μm strainer. After centrifugation, cell pellets were resuspended in ACK lysis buffer (Gibco, no. A10492) for removing red blood cells. Single-cell suspension was then filtered through 70 μm strainer and washed with FACS buffer before antibody staining. For ex vivo stimulation assay, 2 × 10^6^ splenocytes or tumor cells were stimulated with 750x eBioscience Cell Stimulation Cocktail (Thermo Fisher, 00–4970-93) for 4 hours. Brefeldin A Solution (BioLegend, 420601) was added to samples after 1 hour of stimulation.

For staining of single-cell suspension from tumor and spleen, cells were stained for viability (Live/Dead fixable Near-IR, Invitrogen, no. L10119) and treated with Fc blocker (anti-mouse CD16/CD32 monoclonal antibody, Fisher Scientific, no. 50–112-9520), followed by surface staining for 30 min at room temperature. For intracellular staining, washed cells were fixed and permeabilized with Fixation/Permeabilization kit (BD Biosciences, 554714), following the manufacturer’s instructions. After incubation with intracellular antibody cocktail for 30 min, cells were washed twice and resuspended in FACS buffer for flow cytometry. For major immune cells/phenotypes/functionality analysis, antibodies were used at 1:200 dilutions for surface antibodies and 1:100 dilutions for intracellular antibodies. The antibodies used in the immune profiling are listed in table S4.

### Investigation of CRYSTAL in rabbits

All animal procedures were conducted following ethical guidelines and were in accordance with and approved by the Institutional Animal Care and Use Committee (IACUC) at the University of Michigan, Ann Arbor. To establish the VX2 tumor model in rabbits, frozen VX2 tumor tissues (liquid nitrogen, 10% dimethyl sulfoxide of FBS) were rinsed in PBS several times before cutting into small pieces (~1 mm^3^), and around 100 small tumor fragments were resuspended in 10 ml PBS. For each rabbit, both hind limbs were inoculated intramuscularly with 1 ml of VX2 tissue suspension via a 19-gauge needle. All treatments were given via the marginal ear vein. Blood was collected from the central ear artery via a 23-gauge butterfly needle. Serum samples were collected at the indicated time points for liver panel analysis at ULAM pathology core of the University of Michigan. For RT-PCR analysis, tumor and spleen tissues were collected at the indicated time points postmortem. RNA and cDNA were obtained the same as described above. The RT-PCR assay was performed on QuantStudio 5 using TaqMan Gene Expression Assay (FAM). TaqMan primers were purchased from Thermo Fisher Scientific: *Hprt* (Oc03399461_m1), *Ifnb1* (Oc06813776_s1), and *Cxcl10* (Oc06781609_g1).

### Investigation of CRYSTAL in dogs

The dose escalation trial of CRYSTAL in dogs was conducted at Michigan State University to determine the PD and safety profiles of repeated intravenous infusions of CRYSTAL. All animal procedures were conducted following ethical guidelines and were in accordance with and approved by the Institutional Animal Care and Use Committee (IACUC) at Michigan State University. Four purpose-bred naïve beagles (two males and two females; Oak Hill Genetics) were enrolled into the study. Blood samples were collected on D-5 to serve as a baseline. Animals were administered with CRYSTAL by a 30-min intravenous infusion administration at the indicated doses and days. For blood chemistry and hematology analysis, serum and whole blood were collected on 1 day and 7 days after each dose for measurement at Veterinary Diagnostic Laboratory at Michigan State University. For PD analysis, serum and PBMCs were collected at 4 hours and 1 day after each dose for cytokine analysis with ELISA or RT-PCR assays. At the completion of the study, all dogs were adopted.

### Investigation of CRYSTAL in NHPs

The dose escalation trial of CRYSTAL in NHP was conducted at WuXi AppTec to determine immune activation and safety profiles of repeated intravenous infusions of CRYSTAL. The protocol was approved by Wuxi AppTec (Suzhou) Co. Ltd. Institutional Animal Care and Use Committee (Study Number: 400577–2023022401-CPK). Four cynomolgus monkeys (two males and two females), aged 40 to 51 months, were enrolled (WuXi AppTec). In this study, animals were fasted overnight prior to blood collection for clinical pathology. Animals were fed at other times. Blood samples were collected on D-7 to serve as a baseline. Animals were administered with CRYSTAL by a 60 min intravenous infusion via cephalic vein at the indicated doses and days. During the intravenous infusion, plasma samples (0.5 hours) were collected for investigation of C3a via ELISA. For PD analysis, serum was collected at 4 hours, 1 day, and 3 days after each dose for cytokine analysis with ELISA or MSD assays [U-PLEX Custom Biomarker Group 1 (NHP) Assays]. For chemistry and hematology analysis, serum and whole blood were collected on 1 day and 7 days after each dose for measurement.

### Investigation of CRYSTAL in human samples

Human PBMCs were purified from Human Peripheral Blood Leukopak, Fresh (STEMCELL technology), and stored in liquid nitrogen. PBMCs were thawed and plated at 2 × 10^6^ cells per well in a 6-well plate. PBMCs were rested overnight before the treatment with 2.5 μM or 5 μM CRYSTAL-Cy7. After 1 hour incubation, cells were washed with PBS and stained for flow cytometry analysis. The antibodies used in human PBMCs were listed in table S5. For cytokine analysis, human PBMCs was plated at 1 × 10^5^ cells per well in a 96-well plate, and CRYSTAL was incubated with PBMCs overnight for ELISA analysis.

All procedures using human patients’ samples were conducted following guidelines and were in accordance with and approved by Institutional Review Board (IRB) (HUM00113038 and HUM00042189) at University of Michigan, Ann Arbor. Consent was obtained from all participants prior to sample collection. Participants’ age and sex are reported for human patients’ samples and are summarized in the table S6. Primary HNSCC tumors were surgically excised at University of Michigan hospital. On the day of surgical resection, human HNSCC tumors were submerged in RPMI 1640 (Gibco) supplemented with 10% FBS and divided into 2- to 4-mm^3^ sections using a scalpel. Individual sections were then placed in a 48-well plate containing 200 μl of media and injected with vehicle control, free drug STING agonists, or CRYSTAL within each well at indicated doses. After overnight culture, RNA and cDNA were obtained the same as described above. The RT-PCR assay was performed on QuantStudio 5 using TaqMan Gene Expression Assay (FAM). TaqMan primers were pur-chased from Thermo Fisher Scientific: *IFNB1* (Hs01077958_s1) and *PTPRC* (Hs04189704_m1).

For human STING haplotypes sequencing, genomic DNA was extracted from tumor tissues (PureLink Genomic DNA Mini Kit, Thermo Fisher Scientific, K182001). To prepare for DNA sequencing (Eurofins Genomics), primers were designed on the basis of the genomic sequence of *STING1* (NG_034249) and a previous report (11). Thermo Scientific Phusion High-Fidelity DNA Polymerase (2 U/μl) (Fisher Scientific, F530L) was used for PCR (T100 Thermo Cycle, BIO RAD). For exon 3, the forward primer was 5′-GCTGAGACAGGAGCTTTGG-3′ and the reverse primer was 5′-AGCCAGAGAGGTTCAAGGA-3′. For exon 6, the forward primer was 5′-CTGGCCTCCTGTACAATGAGAGT-3′ and the reverse primer was 5′-CAGCTAGGGACACTACAGCTCAGA-3′. For exon 7, the forward primer was 5′-TCAGAGTTGGGTATCAGAGGC-3′ and the reverse primer was 5′-ATCTGGTGTGCTGGGAAGAGG-3′. For sequencing, the following primers were used: For exon 3, the forward primer was 5′-GCTGAGACAGGAGCTTTGG-3′ and the reverse primer was 5′-GGCAGGGCTAGGCATCAAGG-3′. For exon 6, the forward primer was 5′-CTGGCCTCCTGTACAATGAGAGT-3′ and the reverse primer was 5′-CAGCTAGGGACACTACAGCTCAGA-3′. For exon 7, the forward primer was 5′-GGCTTAGTCTGGTCTTCCTCTTACC-3′ and the reverse primer was 5′-ATCTGGTGTGCTGGGAAGAGG-3′.

### Statistical analysis

The results are expressed as means ± standard error of the mean (SEM). One- or two-way analysis of variance (ANOVA), followed by Tukey’s multiple comparison post hoc test or two-sided Students’ *t* test, was used for testing statistical differences among groups. Analyses of animal survival were performed using Kaplan-Meier survival analyses with log-rank Mantel-Cox. All the animal studies were performed after randomization. Data collection and analysis were not performed blind to the conditions of the experiments. The data were approximately normally distributed, and variance was similar between groups. Shown in each figure is a complete dataset from one representative dataset or pooled datasets, as indicated in the figure captions. No samples were excluded from analysis. GraphPad Prism 10.0 (GraphPad Software) was used for statistical analyses.

## Supplementary Material

SI

movie s1

movie s2

movie s3

MDAR Reproducibility Checklist

science.org/doi/10.1126/science.adx1893

Figs. S1 to S37; Tables S1 to S6; Reference (57); MDAR Reproducibility Checklist; Movies S1 to S3

## Data Availability

CRYSTAL is available from J.J.M. under a material transfer agreement with University of Michigan, Ann Arbor. All data needed to evaluate the conclusions in the paper are present in the paper or the supplementary materials or in Dryad (55). DNA sequencing data are deposited in the Dryad (55). The code used for simulation can be accessed in the University of Michigan’s Deep Blue Data repository (56).
