# Supplementary material for: Intermetallic nanoassemblies potentiate systemic STING activation
Source: Science. Author manuscript; Available in PMC 2026 Jul 27. (PMC13403147; doi:10.1126/science.adx1893)
Supplement: SI [file NIHMS2185471-supplement-SI.pdf]

Science AAAS

Supplementary Materials for

Intermetallic nanoassemblies potentiate systemic STING activation

Xingwu Zhou *et al*.

Corresponding author: James J. Moon, moonjj@umich.edu

Science **392**, eadx1893 (2026)
DOI: 10.1126/science.adx1893

The PDF file includes:

Figs. S1 to S37
Tables S1 to S6
References

Other Supplementary Material for this manuscript includes the following:

MDAR Reproducibility Checklist
Movies S1 to S3

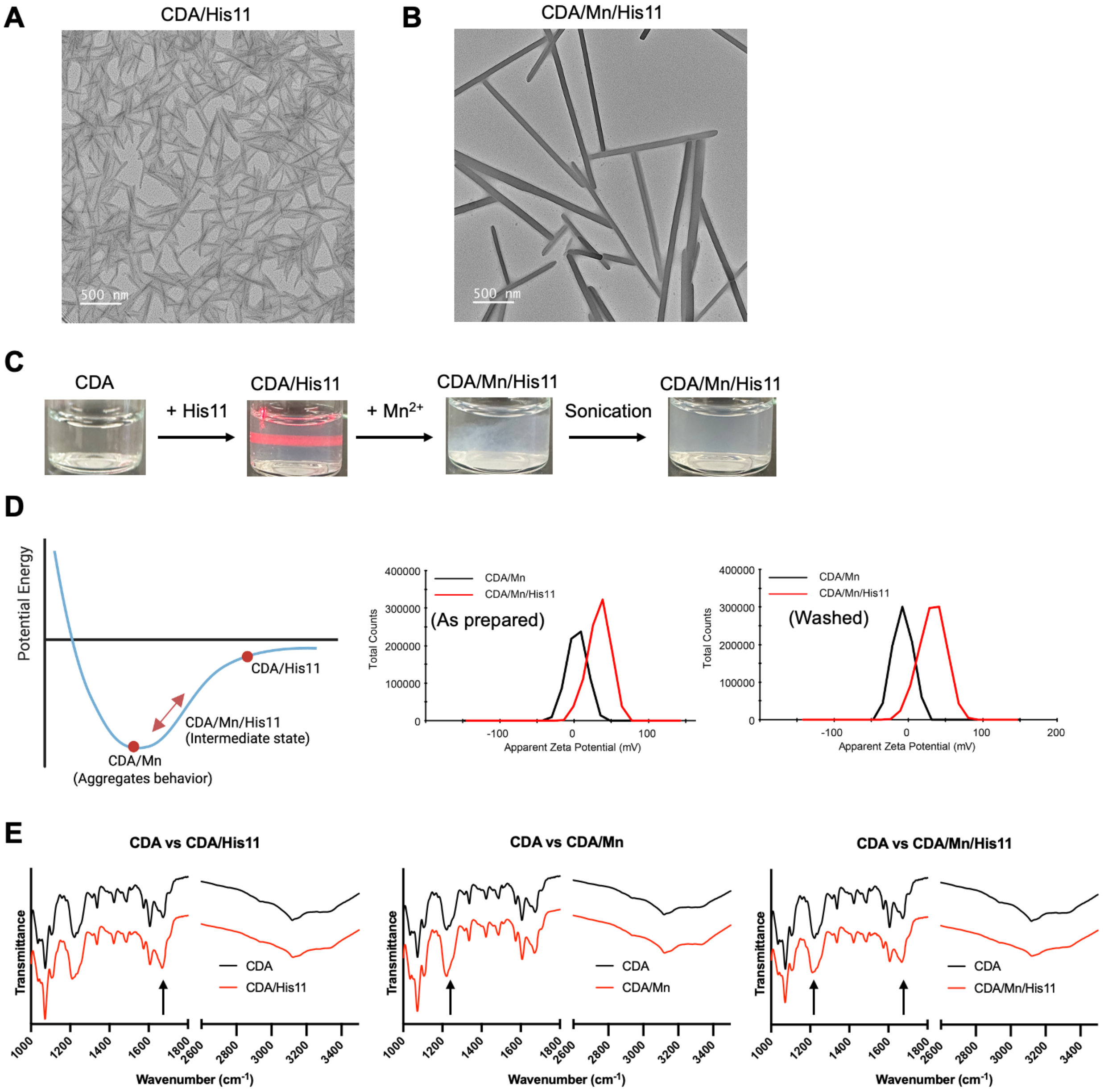

Fig. S1: **A**, **B**, Transmission Electron Microscopy (TEM) images of nanoassemblies of CDA and His 11 (CDA/His11) (**A**) and CDA, His 11, and Mn^{2+} (CDA/Mn/His11) (**B**) (Scale bar: 500nm). **C**, Photographs of the assembling process: the addition of His11 resulted in the self-assembly; the addition of Mn^{2+} led to precipitations; after brief sonication, CDA/Mn/His11 nanoassemblies were formed. **D**, Schematic illustration of different states of nanoassemblies and zeta potential values of as prepared and washed CDA/Mn and CDA/Mn/His11 nanoassemblies. **E**, Fourier-transform infrared (FTIR) spectrum of CDA, CDA/His11, CDA/Mn, and CDA/Mn/His11.

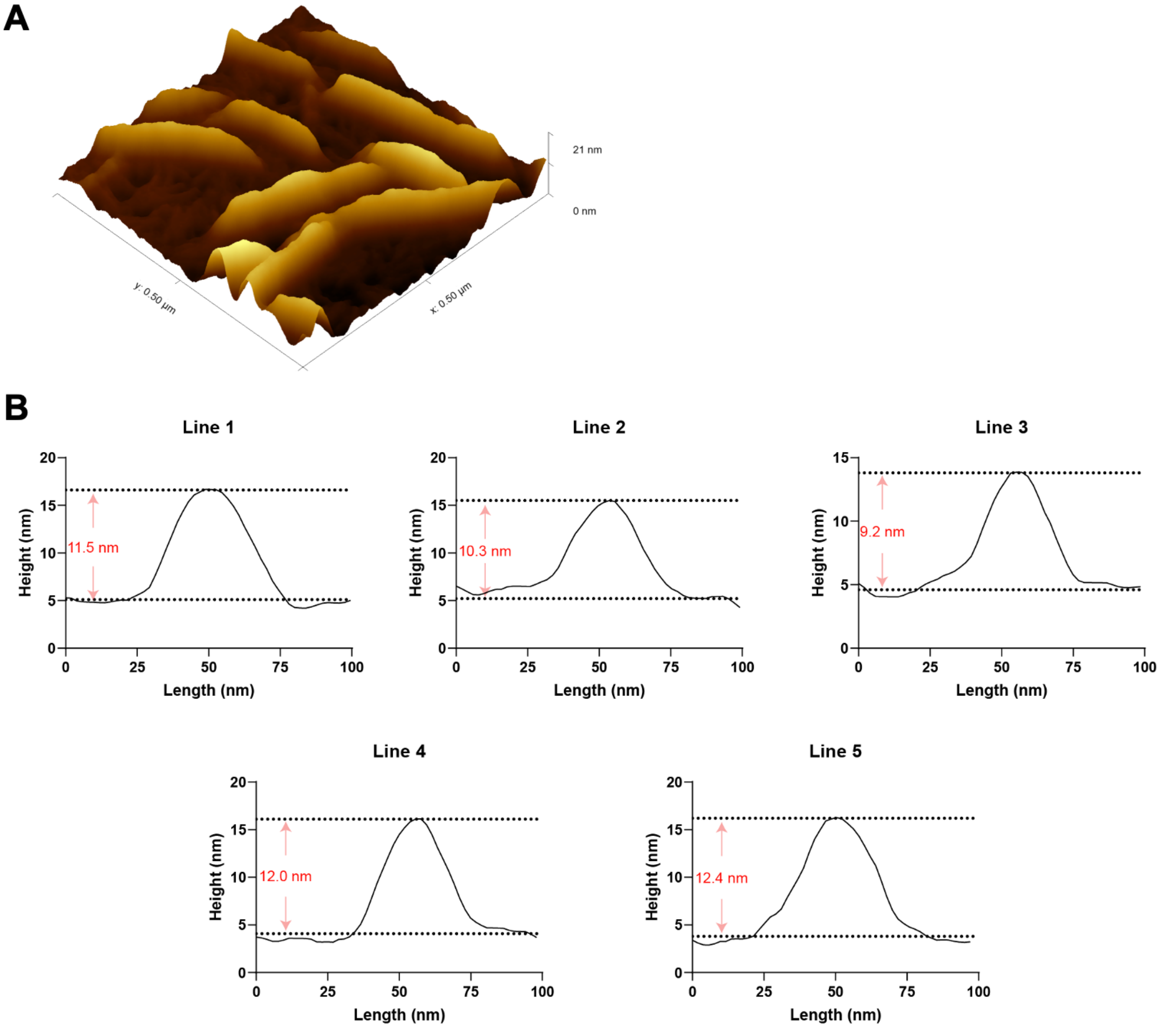

Fig. S2: **A**, Three-dimensional AFM images of CDA/Mn/His11 nanoassemblies. **B**, Height analysis of 5 individual CDA/Mn/His11 nanoassemblies by AFM.

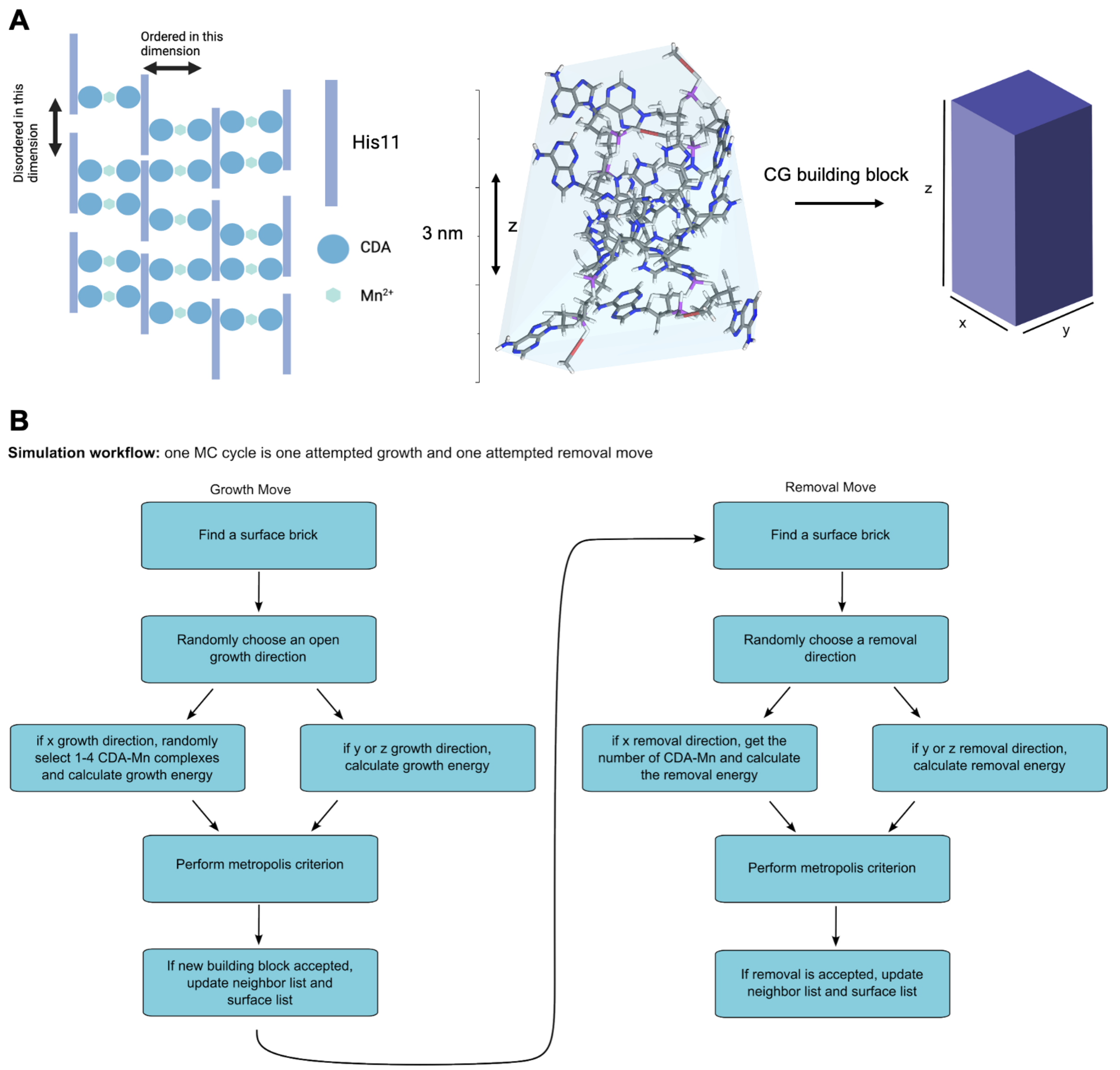

Fig. S3: **A**, Schematics of a coarse-grained CDA/Mn/His11 building block (purple: P; blue: N; red: Mn). **B**, workflow of the steps in a single MC sweep.

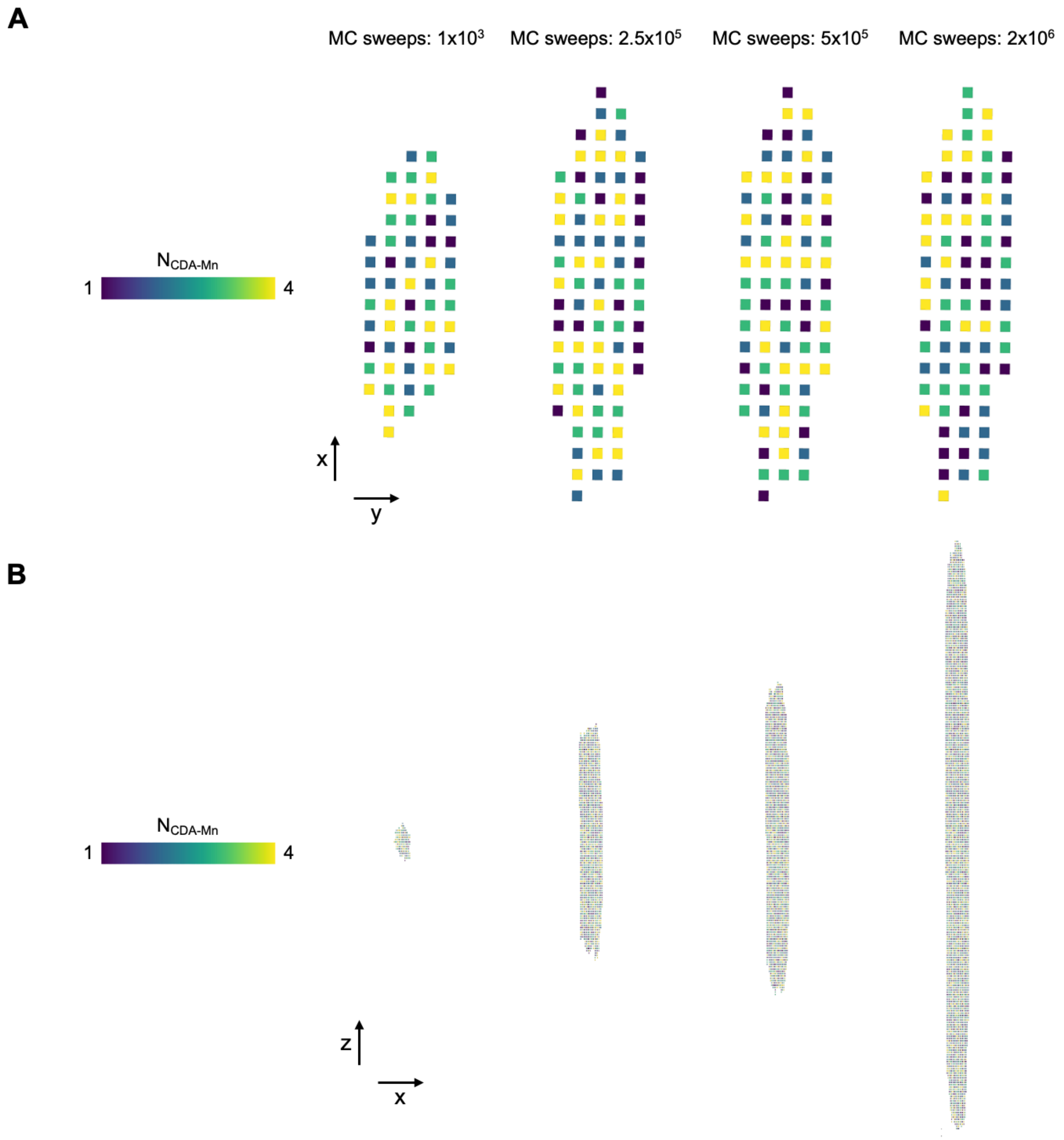

Fig. S4: A, **B**, Snapshots of CDA/Mn/His11 growth during the MC simulations at x-y dimension (**A**) and x-z dimension (**B**).

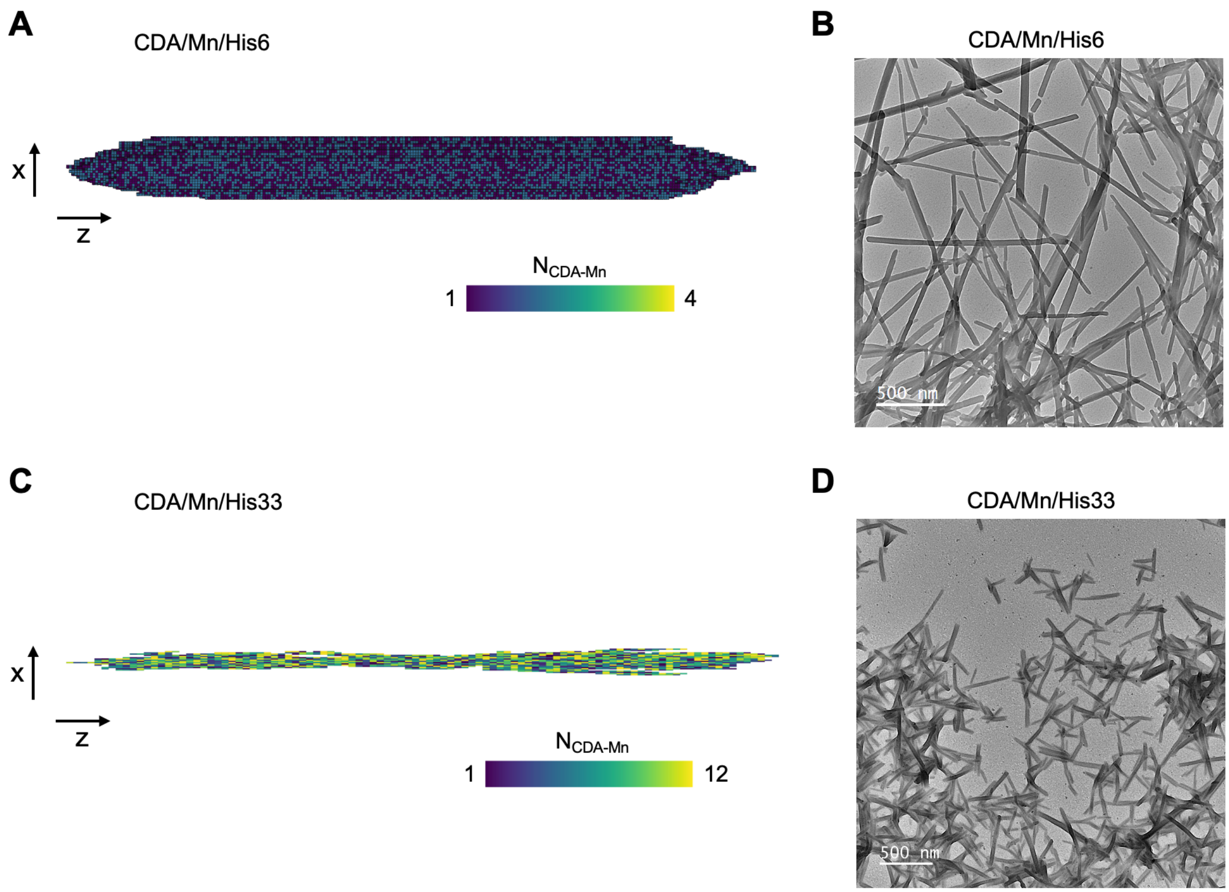

Fig. S5: **A**, Snapshots of CDA/Mn/His6 growth during the MC simulations. **B**, TEM image of CDA/Mn/His6 nanoassemblies (Scale bar: 500nm). **C**, Snapshots of CDA/Mn/His33 growth during the MC simulations. **D**, TEM image of CDA/Mn/His33 nanoassemblies (Scale bar: 500nm).

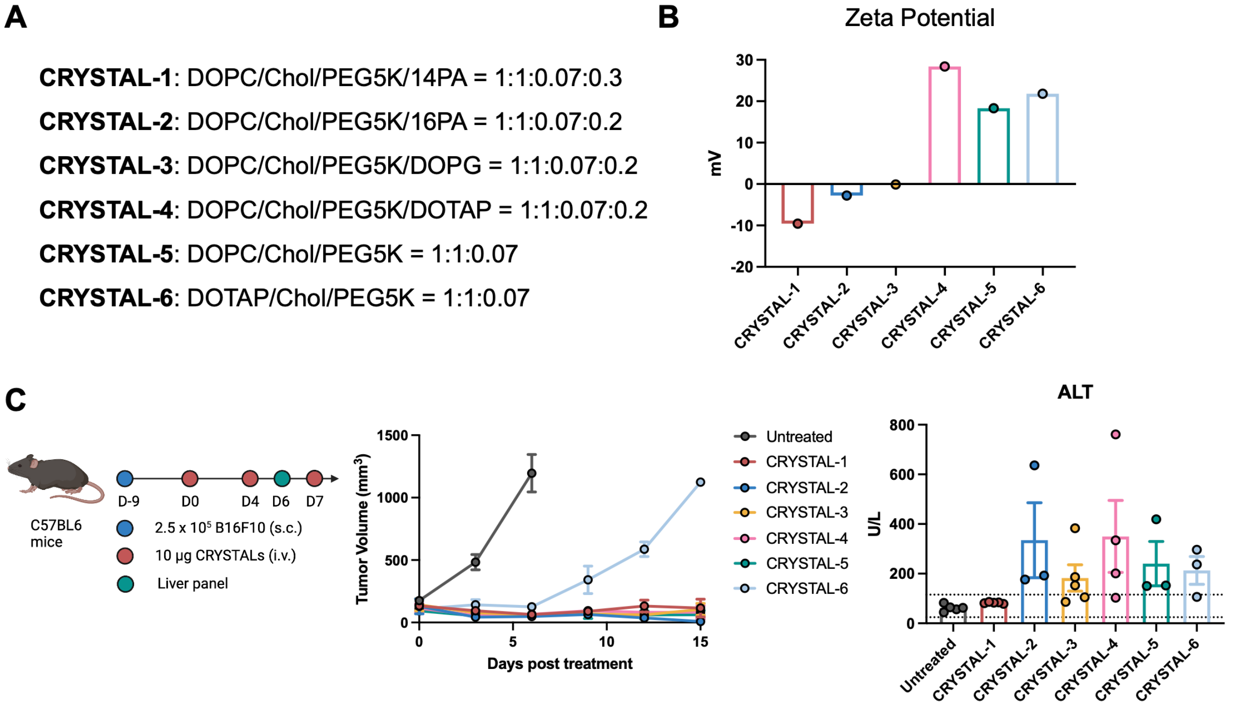

Fig. S6: Screening CRYSTAL by altering the lipid compositions. **A**, molar ratio of the lipid compositions for CRYSTAL-1 to CRYSTAL-6. **B**, Zeta potential of CRYSTAL-1 to CRYSTAL-6 by dynamic light scattering (DLS). **C**, Antitumor efficacy and liver enzyme test of CRYSTAL-1 to CRYSTAL-6 in B16F10 tumor bearing C57BL/6 mice. From left to right: treatment regimen, tumor growth curve, and liver enzyme ALT levels on D6 (dashed grey lines are the normal range). The data represent the mean ± s.e.m. with n = 3-5 biologically independent samples (**C**) and each dot represents an individual mouse (**C**, right panel).

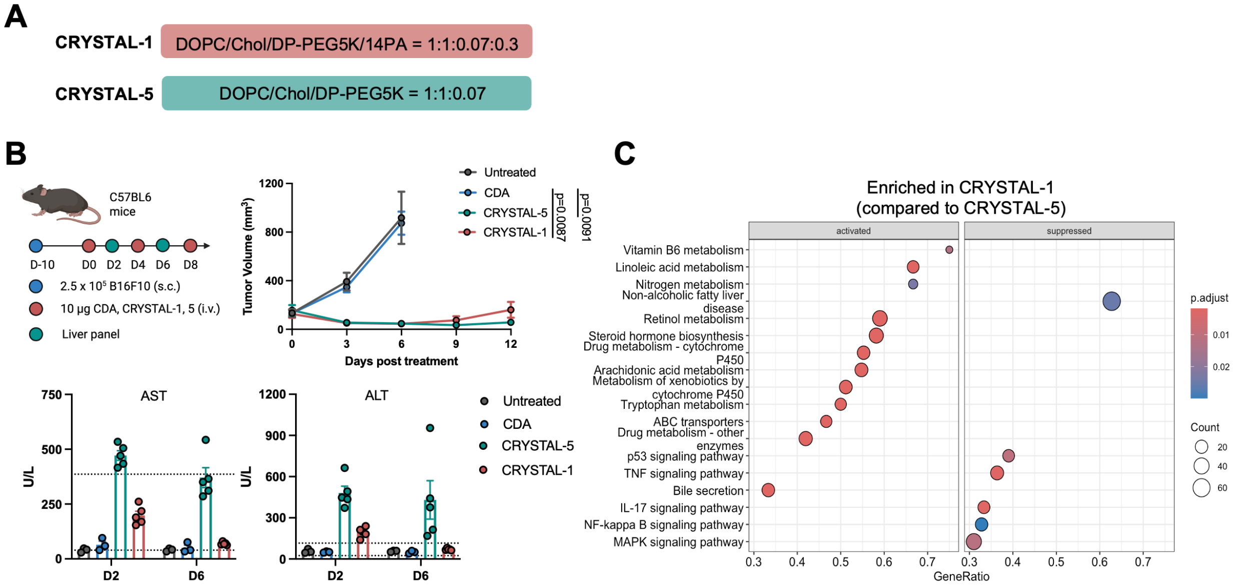

Fig. S7: Comparison of CRYSTAL-1 and CRYSTAL-5 in B16F10 tumor bearing C57BL/6 mice. **A**, molar ratio of the lipid compositions for CRYSTAL-1 and CRYSTAL-5. **B**, Antitumor efficacy and liver enzyme test of CRYSTAL-1 and CRYSTAL-5 in B16F10 tumor bearing C57BL/6 mice: treatment regimen (top left), tumor growth curve (top right), and liver enzyme ALT and AST levels on D2 and D6 (dashed grey lines are the normal range) (bottom). **C**, differential gene expression profile of CRYSTAL-1 and CRYSTAL-5 treated liver isolated on D2 via bulk RNAseq. The data represent the mean ± s.e.m. with n = 3-5 biologically independent samples (**B**) and each dot represents an individual mouse (**B**, bottom). The data were analyzed by two-way ANOVA with Tukey's HSD multiple comparison *post hoc* test for tumor growth curve (**B**).

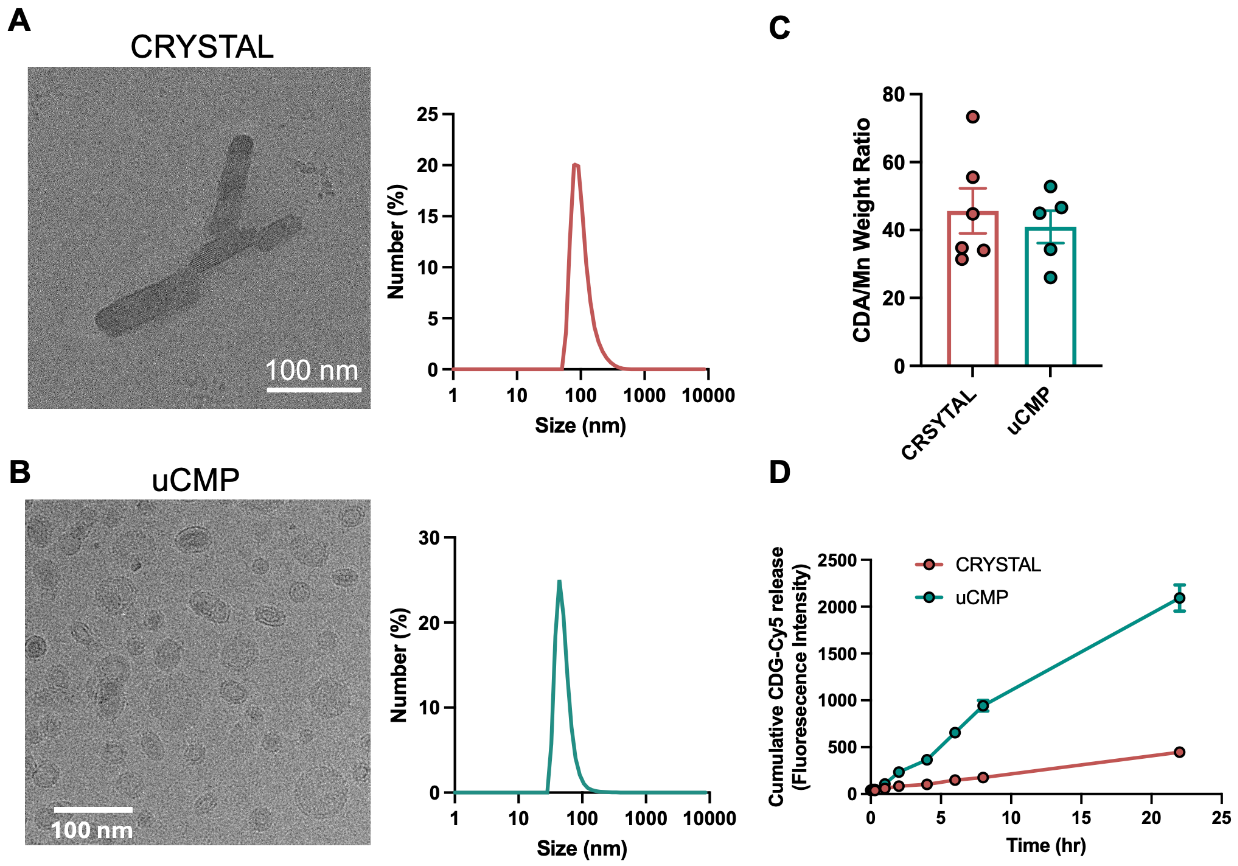

Fig. S8: **A**, Cryo-EM image of CRYSTAL and size distribution profile of CRYSTAL, as determined by DLS. **B**, Cryo-EM image of uCMP and size distribution profile of uCMP, as determined by DLS. **C**, Quantification of CDA/Mn ratio in CRYSTAL and uCMP. **D**, CDA release profile of CRYSTAL and uCMP (CDG-Cy5 labeled) in PBS (37 °C). Released CDA was determined by measuring the fluorescence intensity of Cy5.

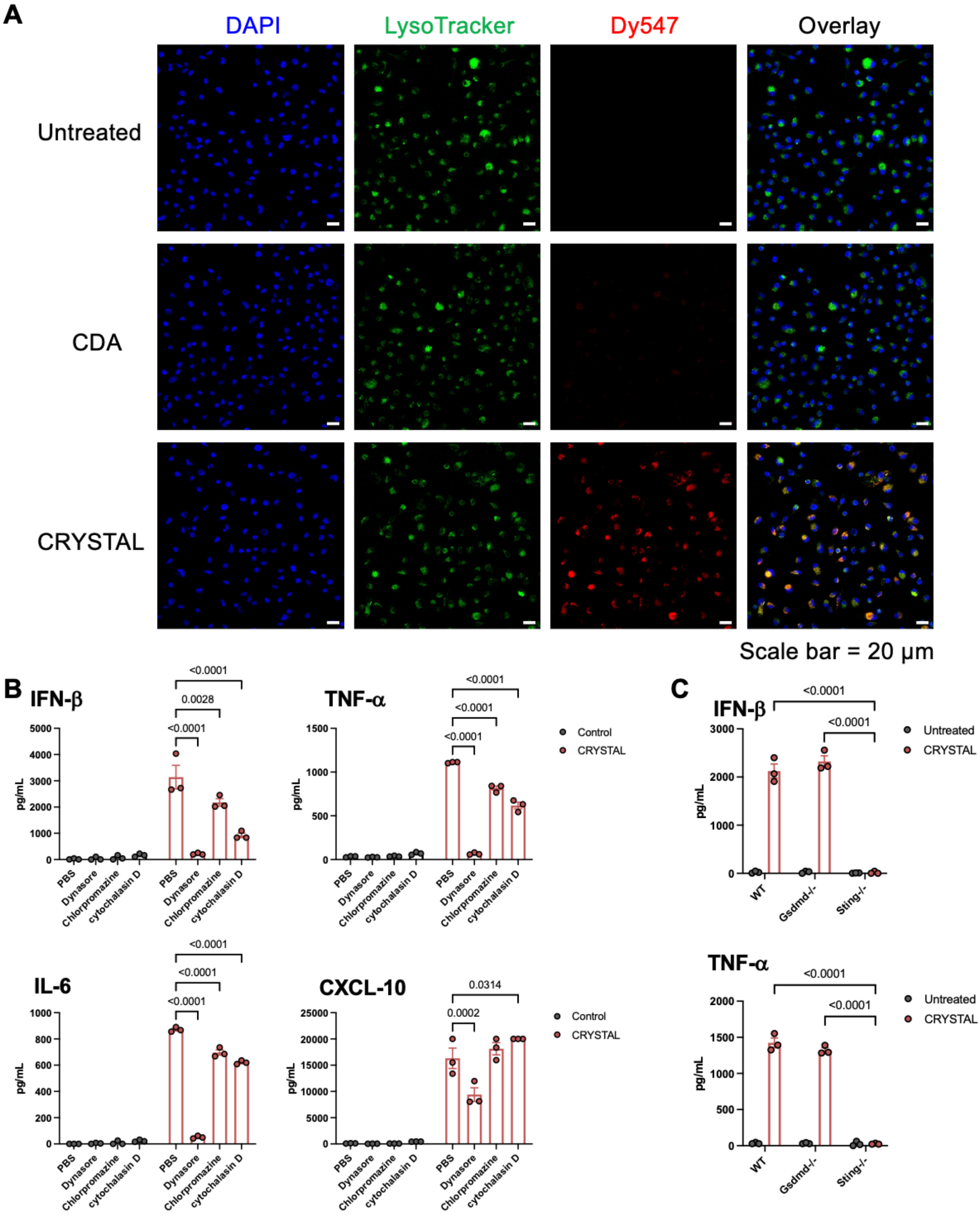

Fig. S9: Investigation of cellular uptake and action pathway of CRYSTAL. **A**, BMDCs were incubated with free CDG-Dy547 or CDG-Dy547 encapsulated in CRYSTAL for 4 hr and then co-stained with DAPI and Lysotracker Green for confocal imaging. **B**, BMDCs were pretreated with different uptake inhibitor (125 μM dynasore, 30 μM chloropromazine, and 2.5 μM cytochalasin D) for 30 min and followed by 10 μg/mL CRYSTAL treatment. After overnight culture, supernatants were used for ELISA analysis of IFN-β, TNF-α, IL-6, and CXCL10. **C**, BMDCs

derived from WT, Gsdmd-/-, and Sting-/- mice were co-cultured with 10 μg/mL CRYSTAL. After overnight culture, supernatants were used for ELISA analysis of IFN-β and TNF-α. The data represent the mean ± s.e.m. with $n = 3$ technically independent samples (**B**, **C**). The data were analyzed by two-way ANOVA, followed by Tukey's HSD multiple comparison *post hoc* test.

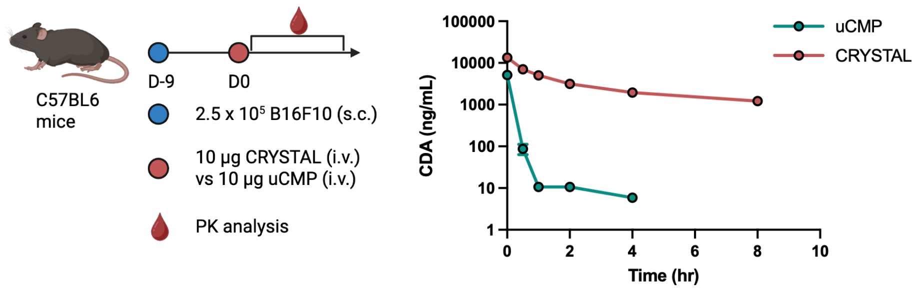

	$t_{1/2}$ (hr)
uCMP	0.08 ± 0.007 hr
CRYSTAL	0.43 ± 0.09 hr

Fig. S10: Pharmacokinetic profile of uCMP and CRYSTAL in B16F10-bearing mice. The data represent the mean ± s.e.m. with n = 5 biologically independent samples. Alpha phase half-life is determined by WinNonlin with two compartment model IV bolus.

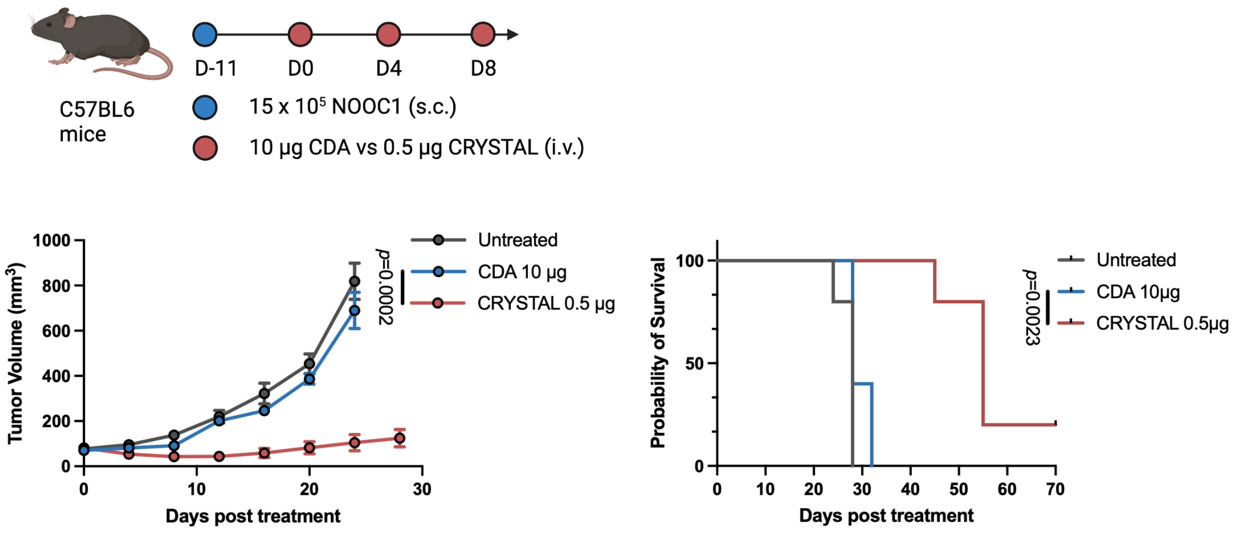

Fig. S11: Investigation of antitumor efficacy of 10 µg CDA and 0.5 µg CRYSTAL in NOOC1 tumor-bearing C57BL/6 mice. Treatment with the indicated dosage was performed on D0, 4, and 8 via IV administration. Tumor size and survival were monitored. The data represent the mean ± s.e.m. with n = 5 biologically independent samples. The experiment was repeated twice and the one additional independent repeat was displayed in **Fig. S37**. The data were analyzed by two-way ANOVA with Tukey's HSD multiple comparison *post hoc* test for tumor growth curve, or log-rank (Mantel-Cox) test for survival curve.

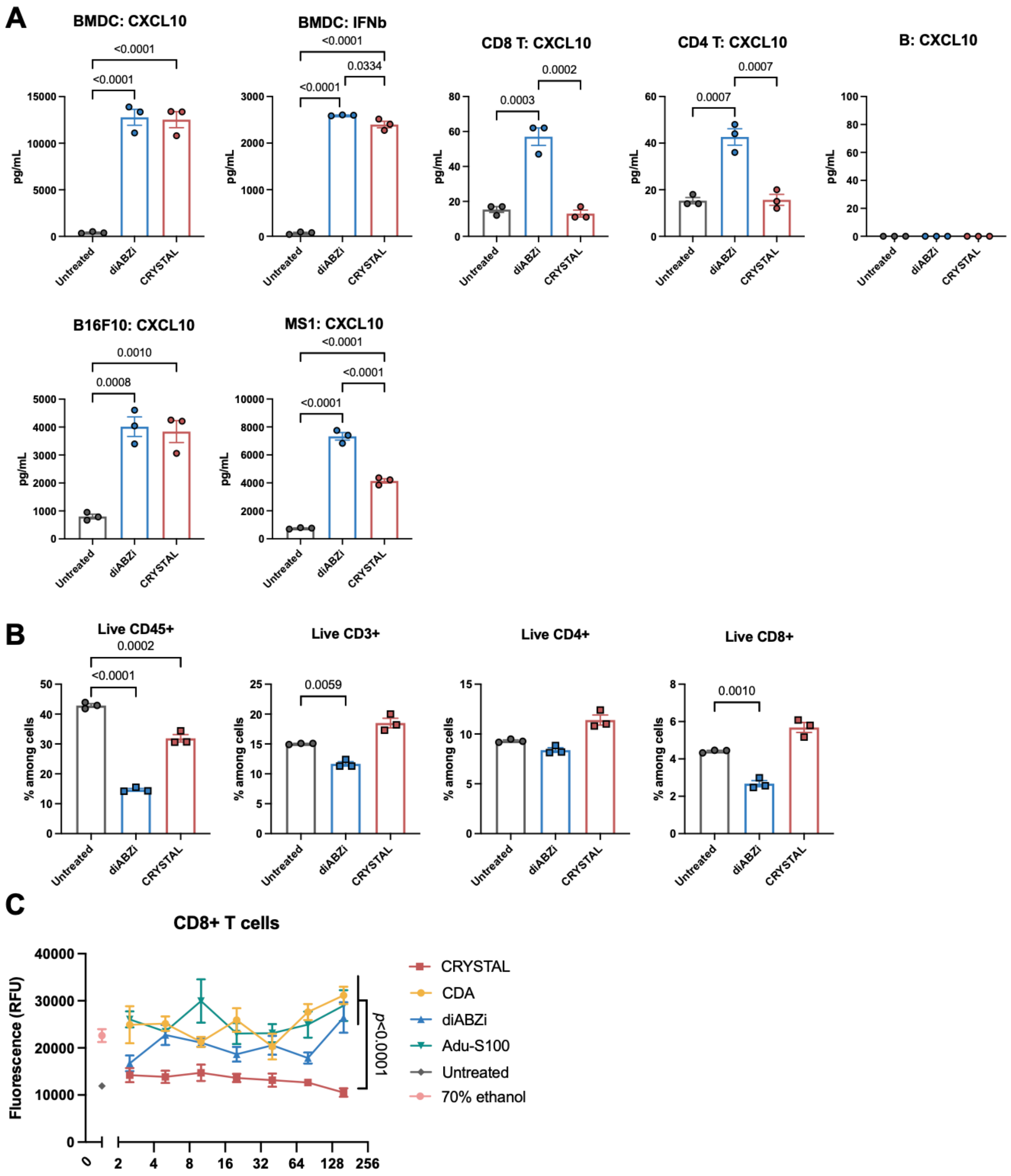

Fig. S12: *In vitro* investigation of diABZi and CRYSTAL: **A**, cytokine analysis of cell supernatants of BMDCs, CD8$^+$ T, CD4$^+$ T, B, B16F10, and MS1 cells after overnight incubation with 10 µg/mL of diABZi and CRYSTAL. **B**, live cell population analysis of freshly isolated splenocytes after 10 hr incubation with 10 µg/mL of diABZi and CRYSTAL. **C**, *in vitro* cytotoxic assay of CD8$^+$ T cells incubated with various STING agonists (CRYSTAL, CDA, diABZi, Adu-S100) at different concentrations for 24 hr. The data represent the mean ± s.e.m. with n = 3 technically independent samples (**A**-**C**). The data were analyzed by one-way ANOVA (**A, B**) and two-way ANOVA (**C**), followed by Tukey's HSD multiple comparison *post hoc* test.

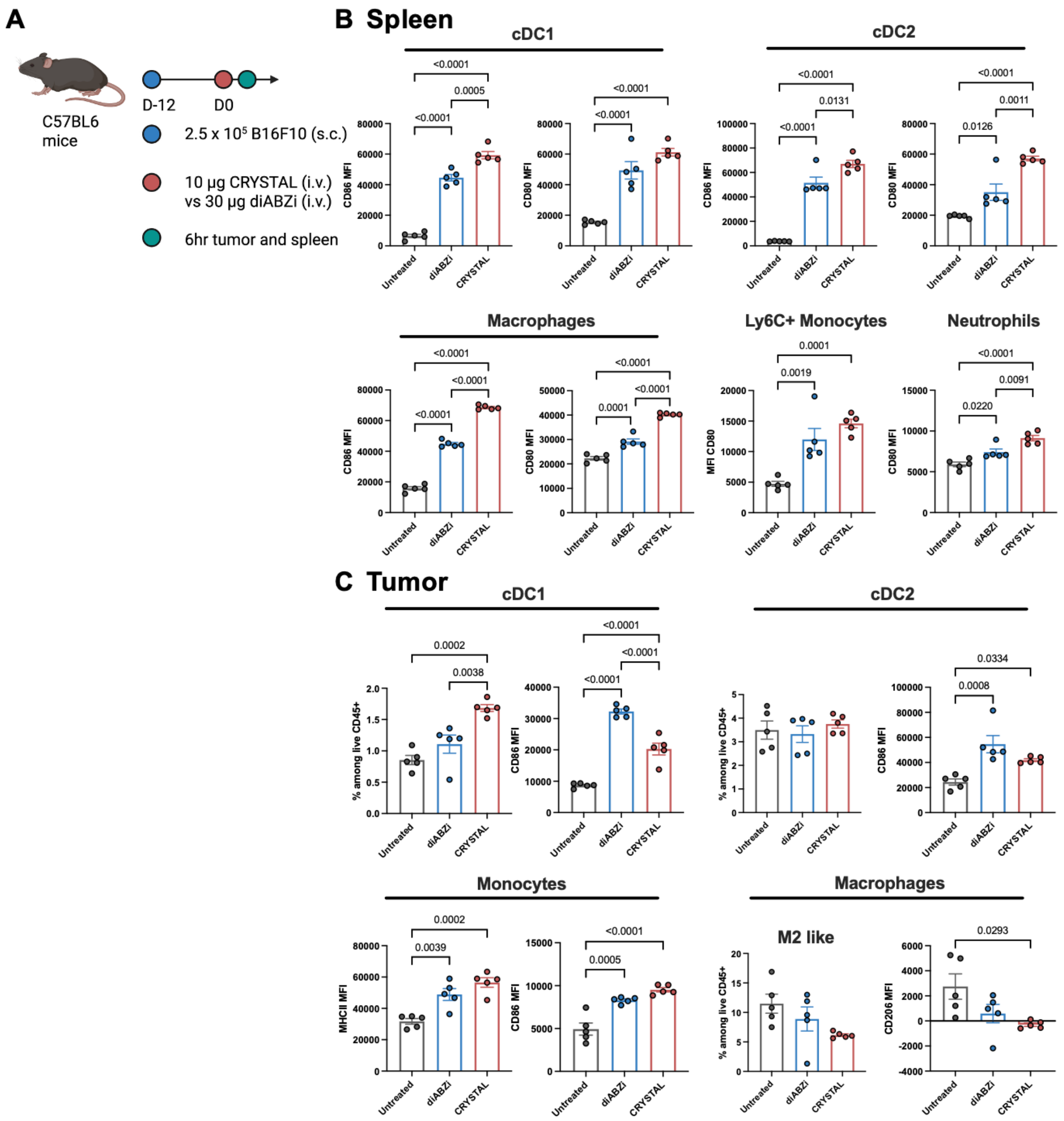

Fig. S13: Investigation of acute innate immune activation of diABZi and CRYSTAL. B16F10 tumor-bearing mice were administered intravenously with either 30 µg diABZi or 10 µg CRYSTAL (**A**). After 6 hr, activation of innate immune cells was analyzed in spleen (**B**) and tumor (**C**). The data represent the mean ± s.e.m. with n = 5 biologically independent samples and each dot represents an individual mouse (**A**-**C**). The data were analyzed by one-way ANOVA, followed by Tukey's HSD multiple comparison *post hoc* test.

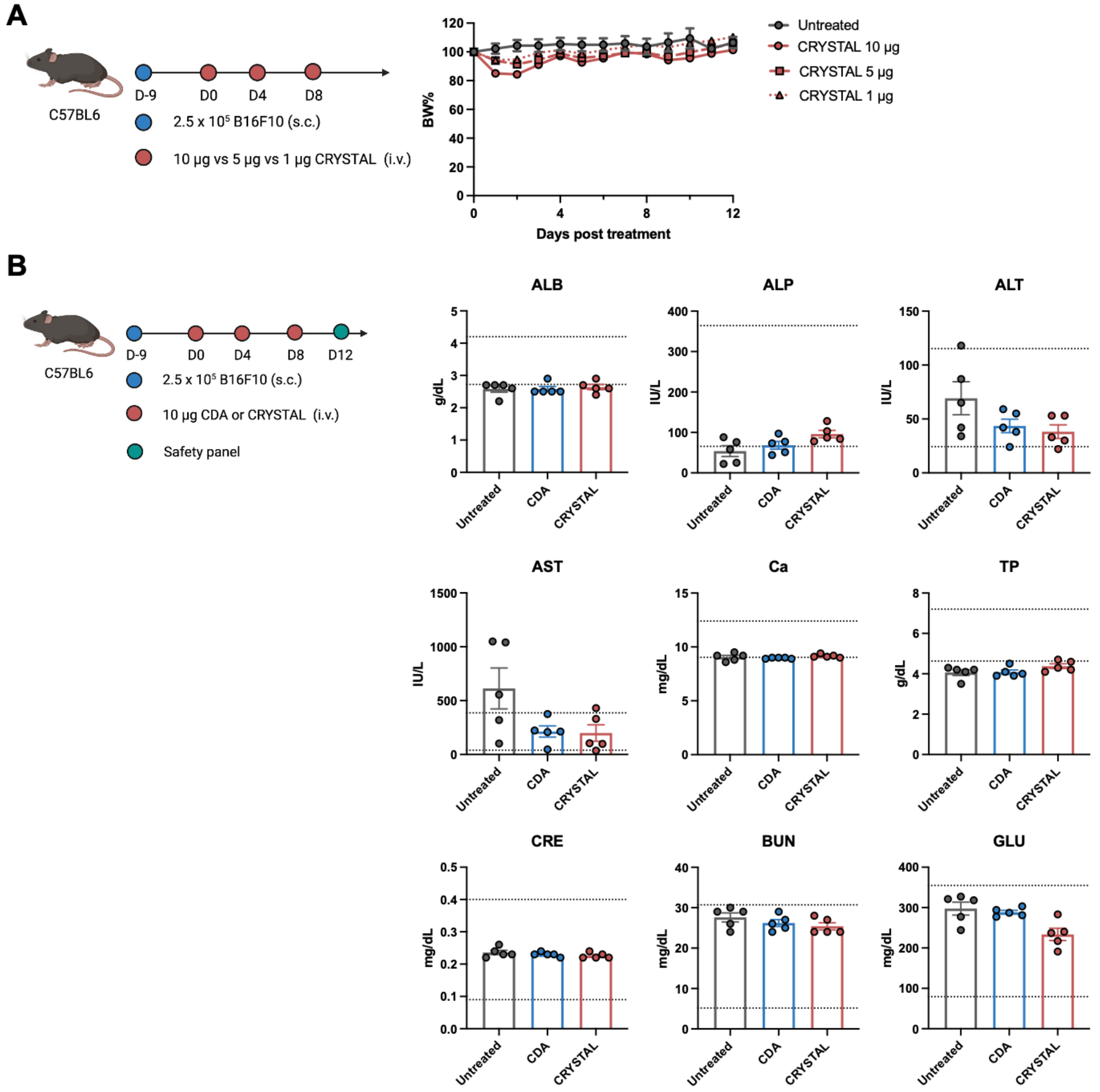

Fig. S14: Safety profiles of systemic CRYSTAL treatment. **A,** 10 µg, 5 µg, and 1 µg CRYSTAL were given intravenously on D0, 4, and 8 in B16F10 tumor-bearing C57BL/6 mice, and bodyweight (BW) were monitored. **B,** 10 µg CDA and CRYSTAL were given intravenously on D0, 4, and 8 in B16F10 tumor-bearing C57BL/6 mice, and chemistry panel were measured on D12. Dotted grey lines mark normal ranges of each parameter. (ALB: Albumin, ALP: Alkaline phosphatase, ALT: Alanine transaminase, AST: Aspartate aminotransferase, Ca: Calcium, TP: Total protein, CRE: Creatinine, BUN: Urea nitrogen, GLU: glucose) The data represent the mean ± s.e.m. with n = 5 biologically independent samples (**A**, **B**) and each dot represents an individual mouse (**B**).

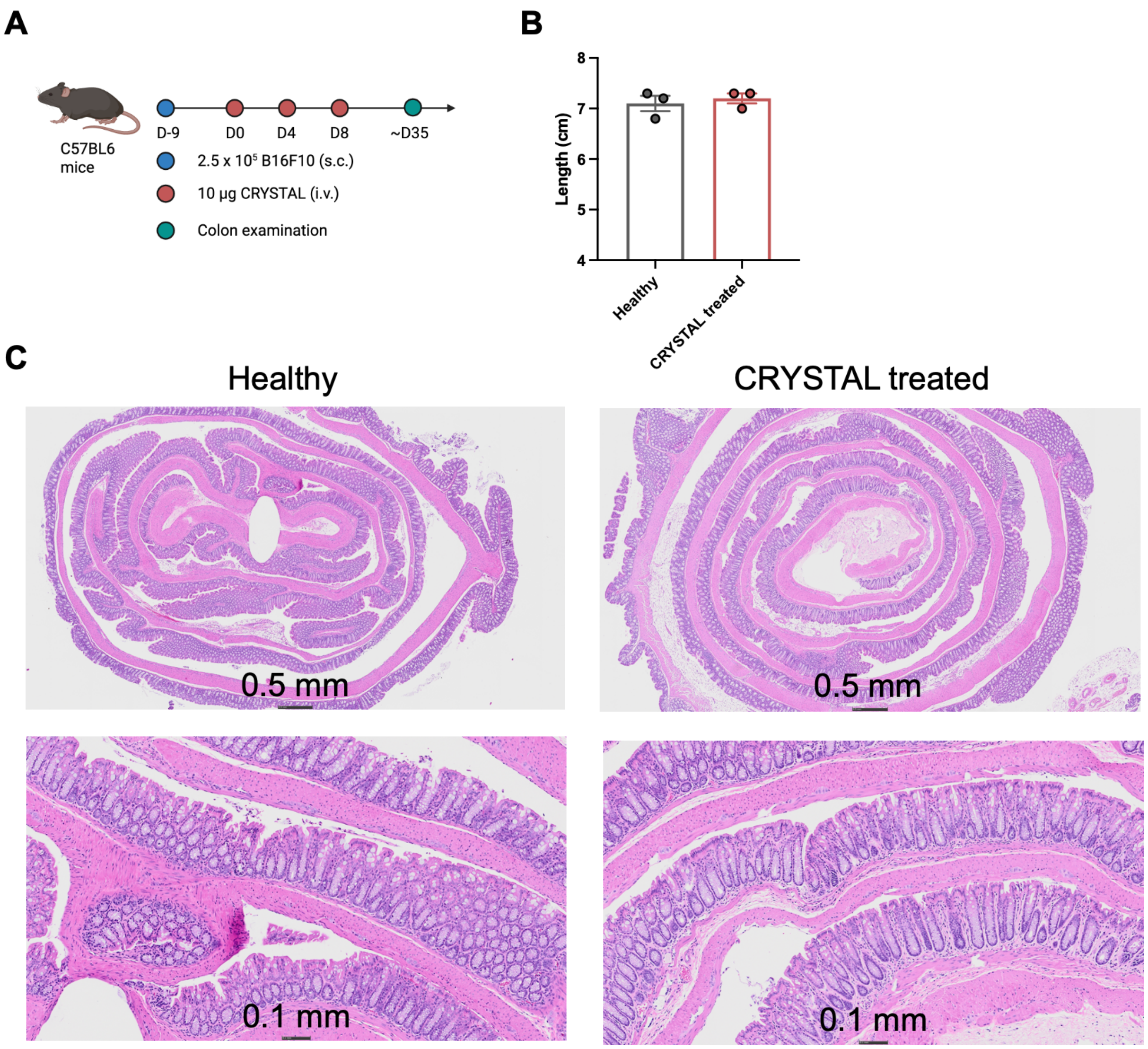

Fig. S15: Investigation of long-term immunotherapy related adverse events in colon. In B16F10 bearing mice, after three cycles of CRYSTAL treatment (**A**), the colon from survivors were obtained for measuring its length (**B**) and the histopathological HE evaluation (**C**). The data represent the mean ± s.e.m. with n = 3 biologically independent samples and each dot represents an individual mouse (**B**). The data were analyzed by unpaired two-sided Student's *t*-test.

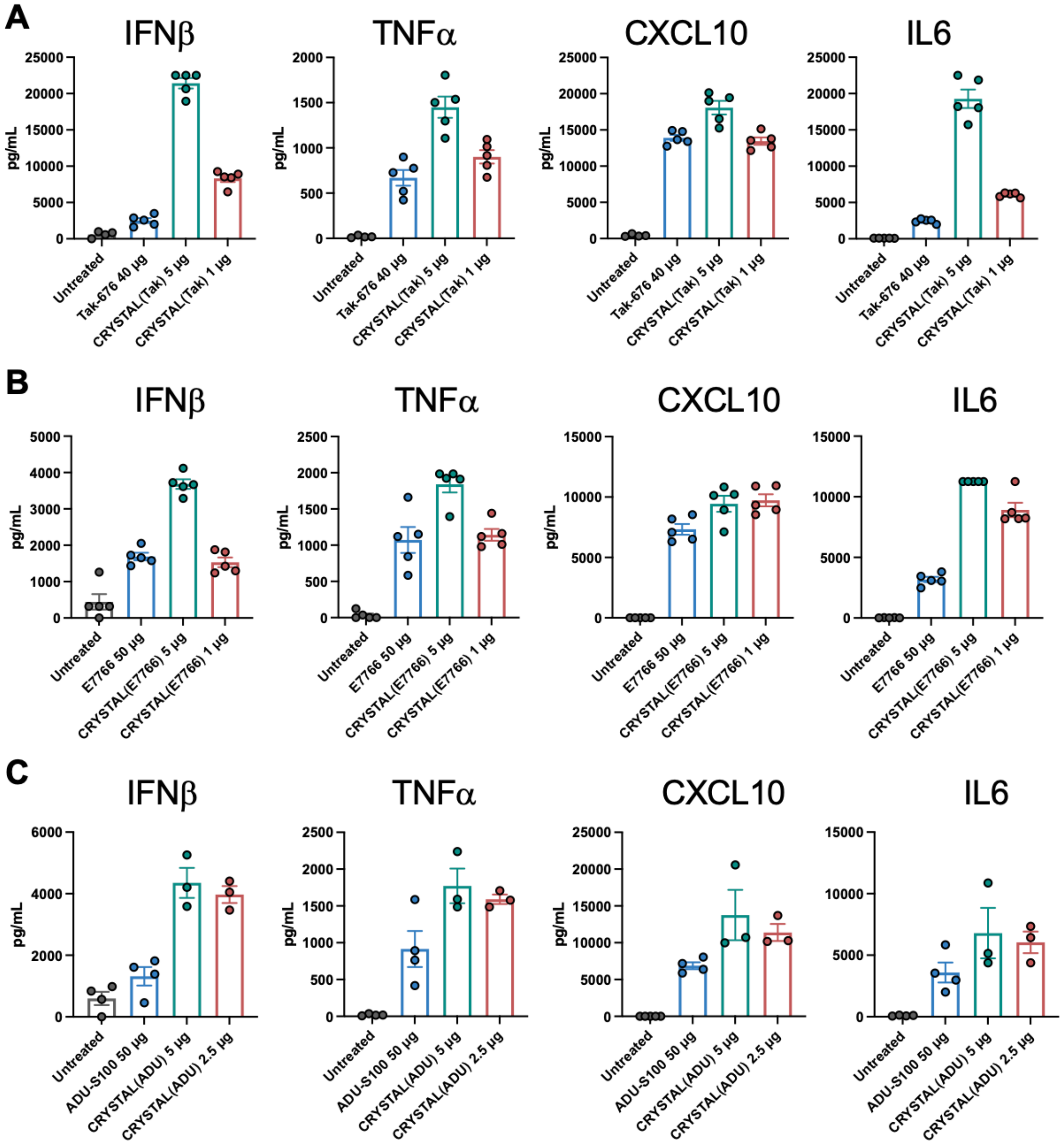

Fig. S16: A-C, Cytokine profile for CRYSTAL(Tak), CRYSTAL(E7766), CRYSTAL(Adu). Investigation of systemic STING activation of CRYSTALs carrying various STING agonists, including Tak-676 (**A**), E7766 (**B**), and ADU-S100 (**C**) in B16F10 tumor-bearing C57BL/6 mice. Serum IFN-β, TNF-α, CXCL10, and IL-6 were measured via ELISA at 4 hr after IV administration of each drug. The data represent the mean ± s.e.m. with n = 3-5 biologically independent samples and each dot represents an individual mouse (**A**-**C**).

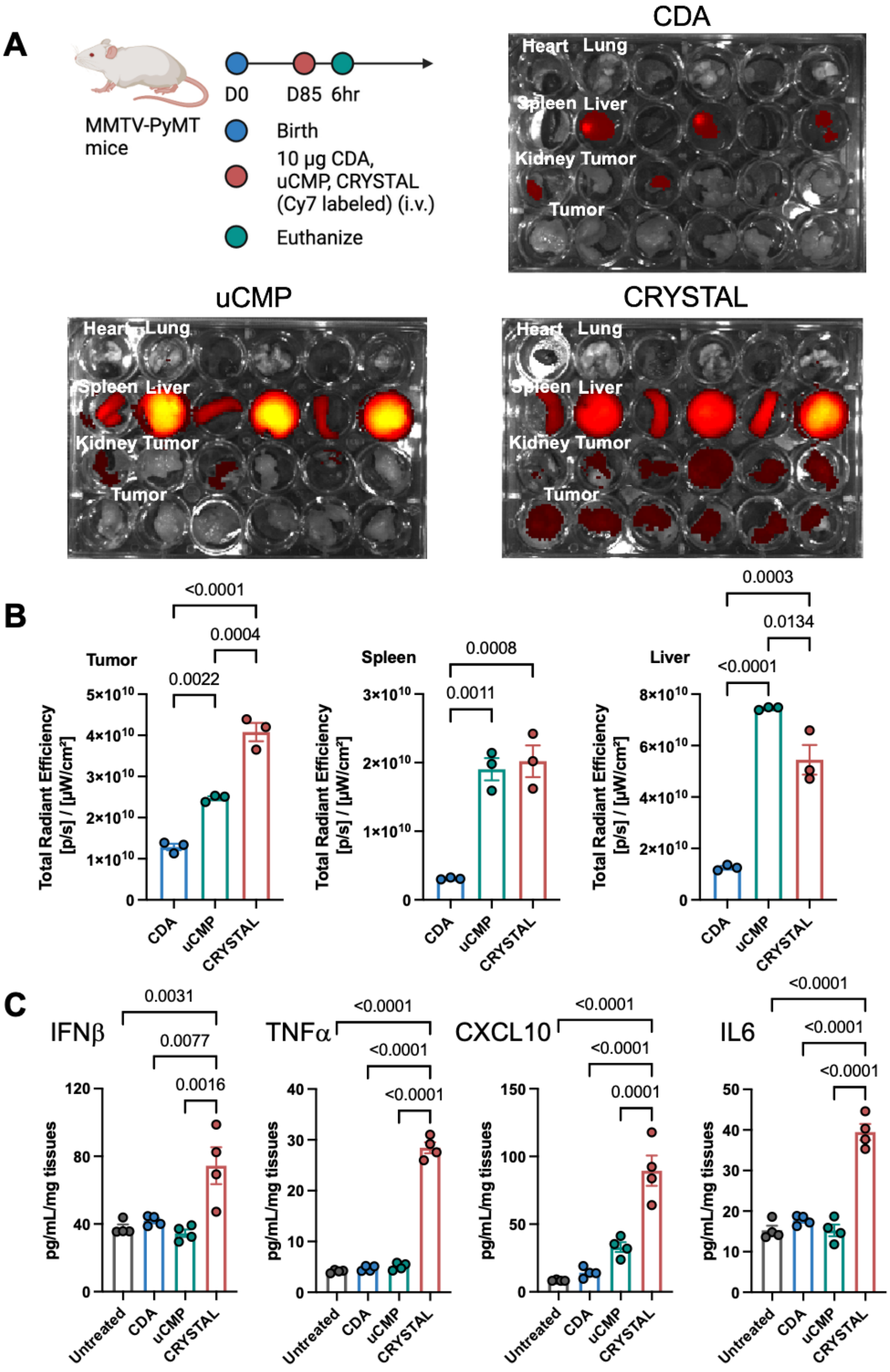

Fig. S17: **A**, Biodistribution of CDA, uCMP, and CRYSTAL (each drug labeled with Cy7) was determined with IVIS. MMTV-PyMT mice (Age ~D85) were injected IV with 10 µg CDA, uCMP, and CRYSTAL, and major organs (heart, lung, spleen, liver, kidney) and 3 random tumors (throughout the mammary pads) were collected for IVIS imaging at 6 hr after IV injection. **B**, Quantification of total radiant efficiency of tumor, spleen, and liver. **C**, Tumors were collected and homogenized to examine tumor intrinsic STING activation profile. IFN-β, TNF-α, CXCL10, and

IL-6 in tumor homogenates were measured via ELISA at 6 hr after IV administration of each drug. The data represent the mean ± s.e.m. with n = 3 (**A, B**) and n = 4 (**C**) biologically independent samples and each dot represents an individual mouse (**B**, **C**). The datasets were analyzed by one-way ANOVA, followed by Tukey's HSD multiple comparison *post hoc* test.

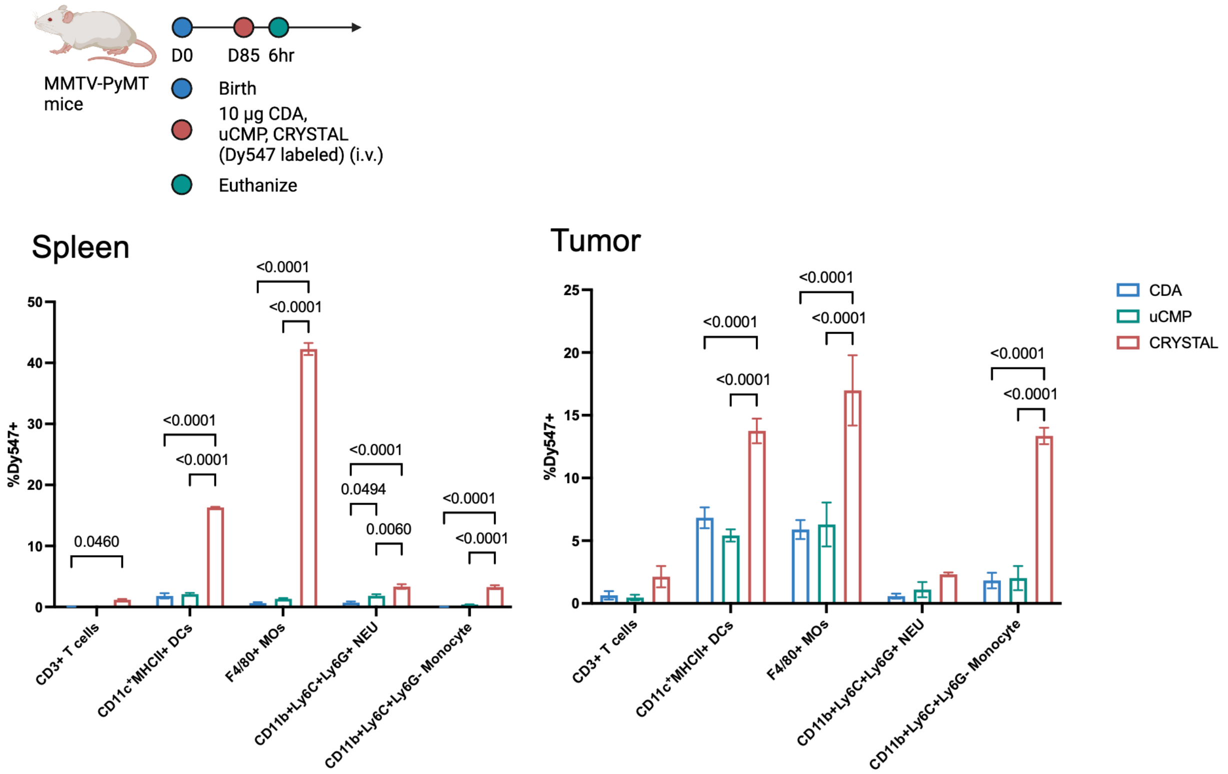

Fig. S18: Cellular uptake of CDA, uCMP, and CRYSTAL (using drug labeled with Dy547) was determined with flow cytometry. MMTV-PyMT mice (Age ~D85) were IV injected with 10 μg CDA, uCMP, and CRYSTAL, spleen and tumor were collected to examine immune cellular uptake. The data represent the mean ± s.e.m. with n = 4 biologically independent samples. The data were analyzed by two-way ANOVA with Tukey's HSD multiple comparison *post hoc* test.

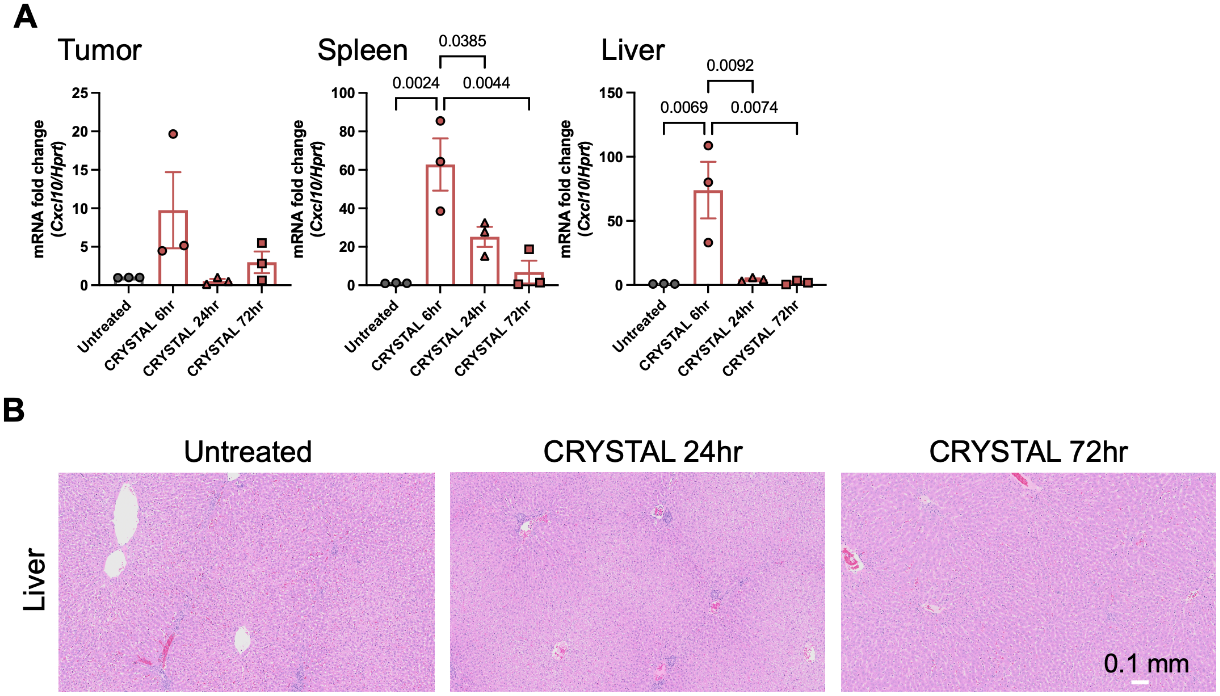

Fig. S19: **A**, Pharmacodynamics of STING activation in rabbits. RT-PCR analysis of *Cxcl10* expression in tumor, spleen, and liver at 6, 24, and 72 hr post IV injection of 0.5 mg CRYSTAL in VX2 tumor-bearing rabbits. **B**, the histopathological HE evaluation of liver at 24 and 72 hr post IV injection of 0.5 mg CRYSTAL in VX2 tumor-bearing rabbits in comparison to the untreated rabbits. The data represent the mean ± s.e.m. with n = 3 (**A, B**) biologically independent samples and each dot represents an individual rabbit (**A**). The data were analyzed by one-way ANOVA, followed by Tukey's HSD multiple comparison *post hoc* test.

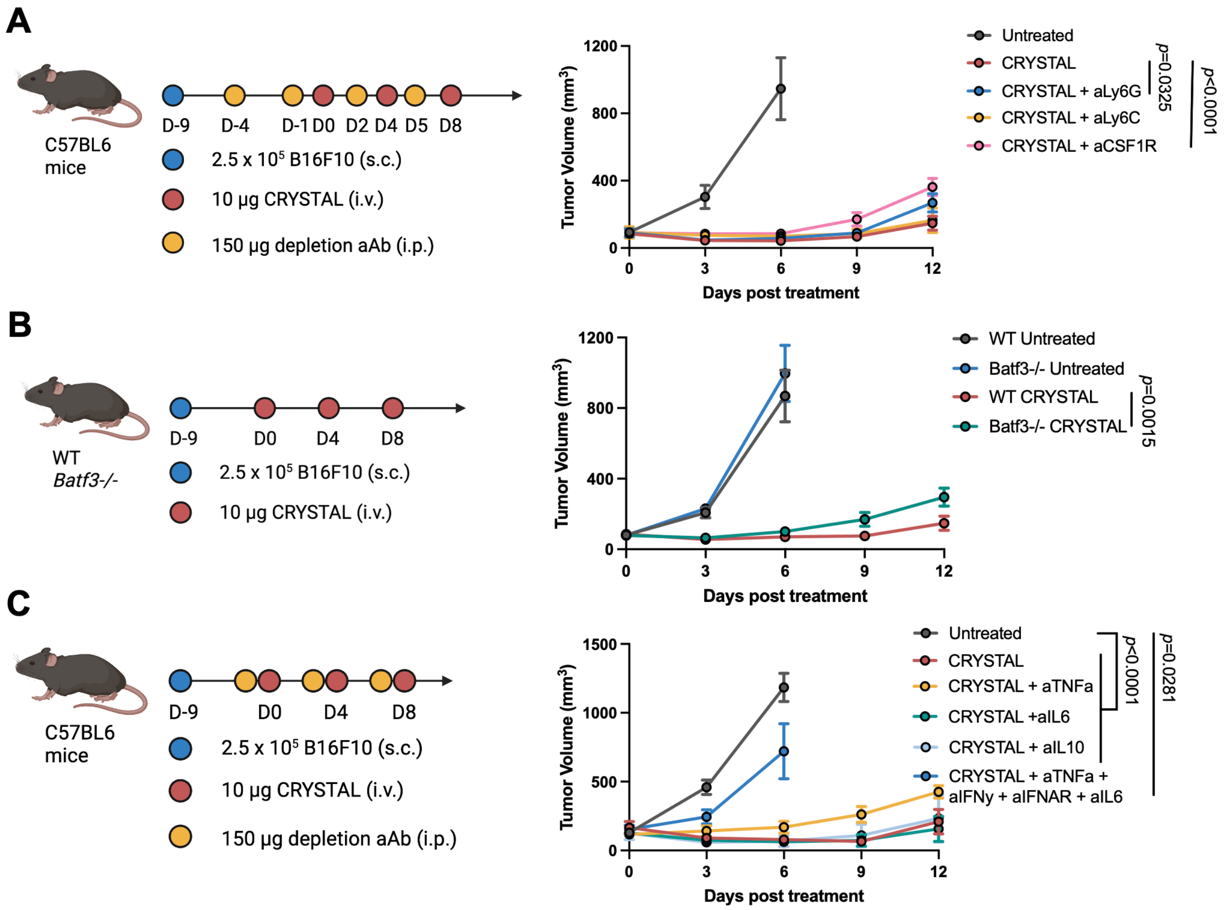

Fig. S20: **A**, Antitumor efficacy of CRYSTAL after antibody-mediated depletion of various immune cells (anti-Ly6G, anti-Ly6C, and anti-CSF1R) in B16F10 tumor-bearing mice. **B**, Antitumor efficacy of CRYSTAL in B16F10 tumor-bearing WT and *Batf3*$^{-}$/$^{-}$ C57BL/6 mice. **C**, Antitumor efficacy of CRYSTAL after antibody-mediated depletion of various cytokines (anti-TNF-α, anti-IL-6, anti-IL-10, anti-IFN-γ, and anti-IFNAR) in B16F10 tumor-bearing mice. The data represent the mean ± s.e.m. with n = 5 (**A**, **B**) and n= 4-5 (**C**) biologically independent samples. The data were analyzed by two-way ANOVA with Tukey's HSD multiple comparison *post hoc* test for tumor growth curve.

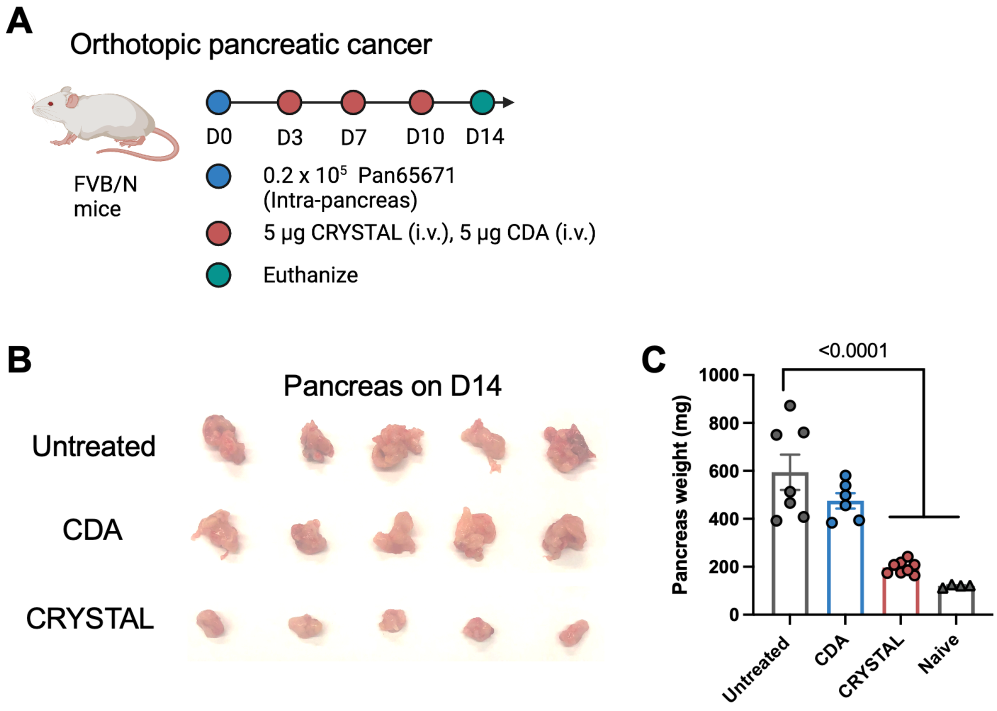

Fig. S21: Antitumor efficacy of CDA and CRYSTAL in an orthotopic pancreatic cancer model: treatment regimen (**A**), representative tumor images on D14 post treatment (**B**), and pancreas weight on D14 post treatment (**C**). The data represent the mean ± s.e.m. with n = 4-7 biologically independent samples and each dot represents an individual mouse (**C**). The experiment was repeated twice and the one additional independent repeat was displayed in **Fig. S37**. The data were analyzed by one-way ANOVA, followed by Tukey's HSD multiple comparison *post hoc* test.

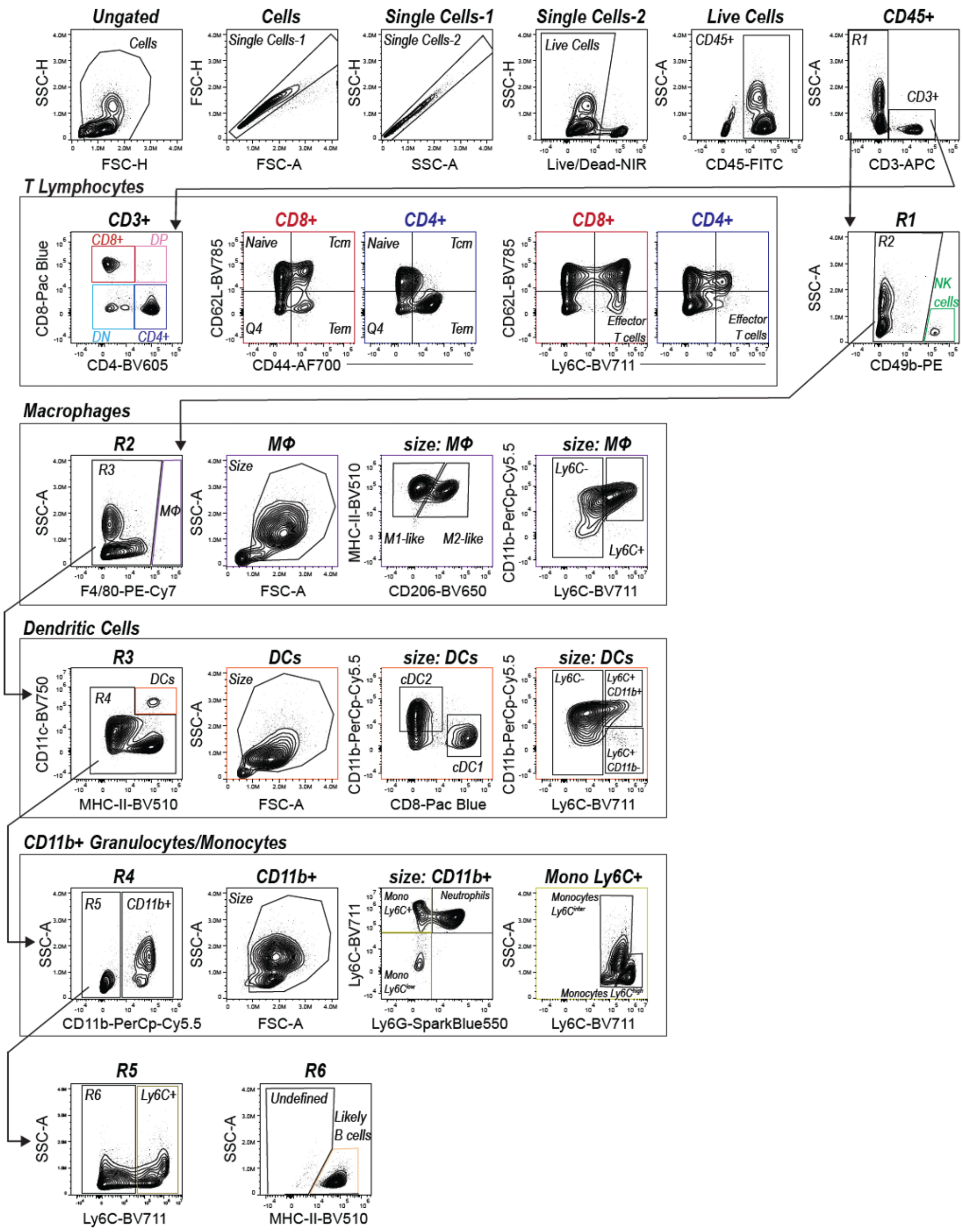

Fig. S22: Representative gating strategy of major immune cell populations in spleen of MMTV-PyMT mice.

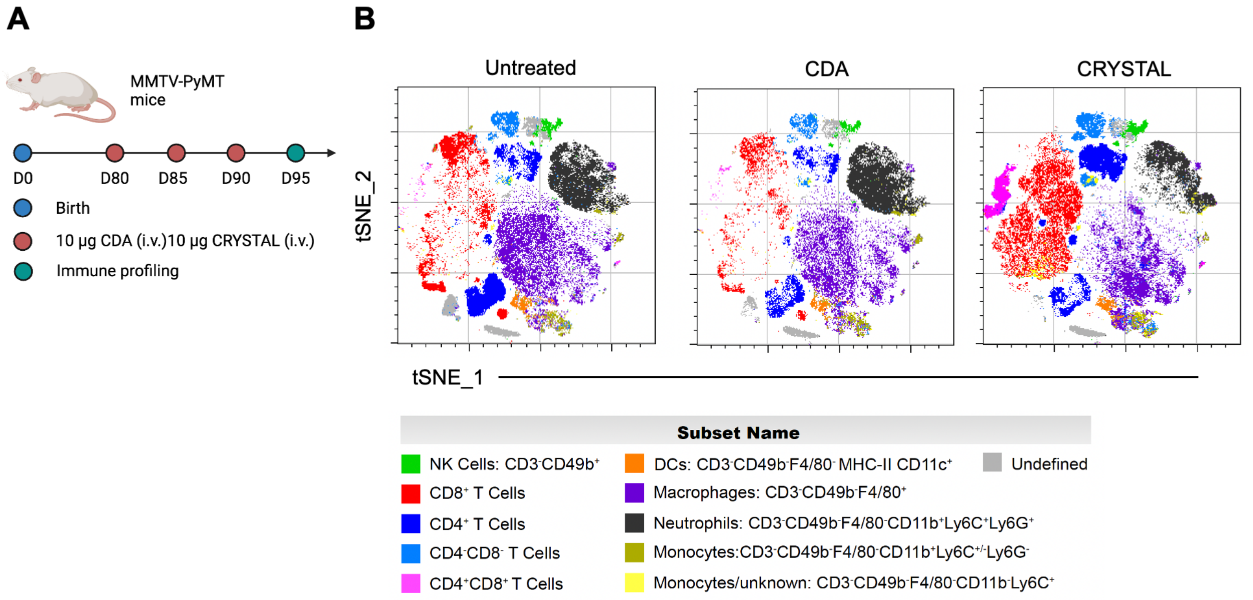

Fig. S23: **A**, MMTV-PyMT mice (around D80 post birth) were enrolled and given IV injections of 10 µg CDA or CRYSTAL on D0, D5, and D10. Tumor tissues were collected for immune profiling on D15. **B**, tSNE plots of tumor CD45$^+$ immune cells among untreated, CDA, and CRYSTAL groups. The data represent the concatenated populations with n = 5 biologically independent samples. The experiment was repeated twice and the one additional independent repeat was displayed in Dryad (*55*).

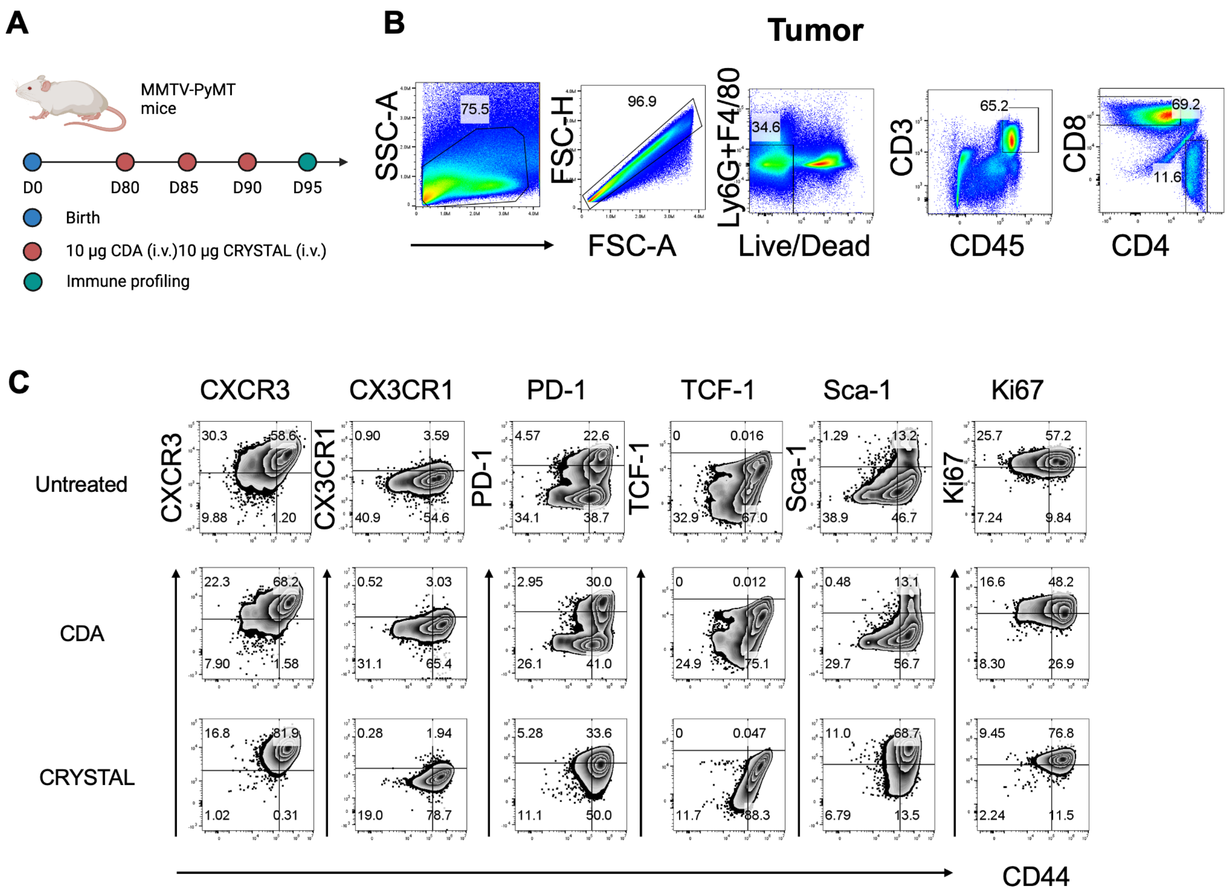

Fig. S24: A-C, MMTV-PyMT mice (around D80 post birth) were enrolled and given IV injections of 10 µg CDA or CRYSTAL on D0, D5, and D10. Tumor tissues and spleen were collected for immune profiling on D15 (**A**). Gating strategy in tumor (**B**) and representative plots of concatenated tumor $CD8^{+}$ T cells (**C**). The data represent the concatenated populations with n = 5 biologically independent samples.

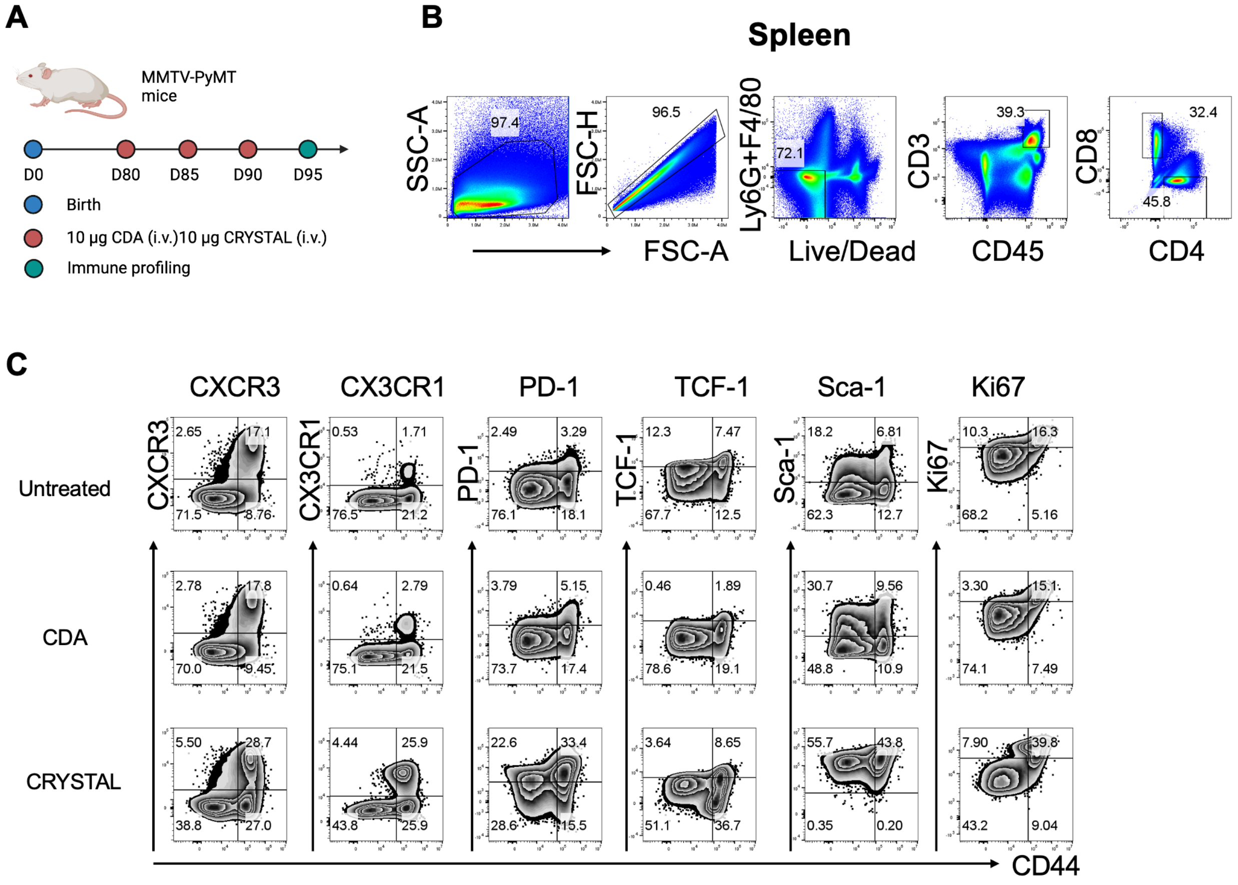

Fig. S25: A-C, MMTV-PyMT mice (around D80 post birth) were enrolled and given IV injection of 10 µg CDA or CRYSTAL on D0, D5, and D10. Tumor tissues and spleen were collected for immune profiling on D15 (**A**). Gating strategy in spleen (**B**) and representative plots of spleen CD8^{+} T cells (**C**). The data represent the concatenated populations with n = 5 biologically independent samples.

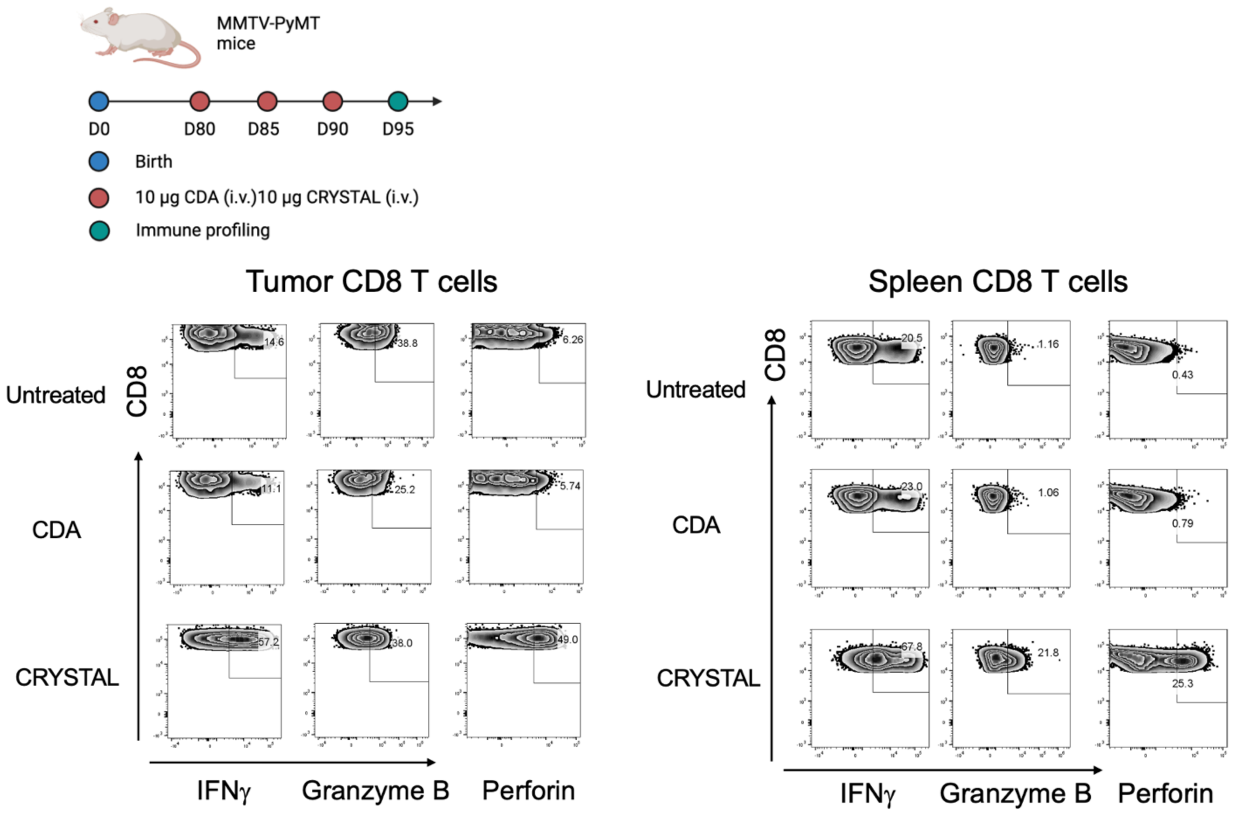

Fig. S26: Representative plots of *ex vivo* activation of CD8^{+} T cell in tumor and spleen in the MMTV-PyMT model. The data represent concatenated populations with n = 5 biologically independent samples.

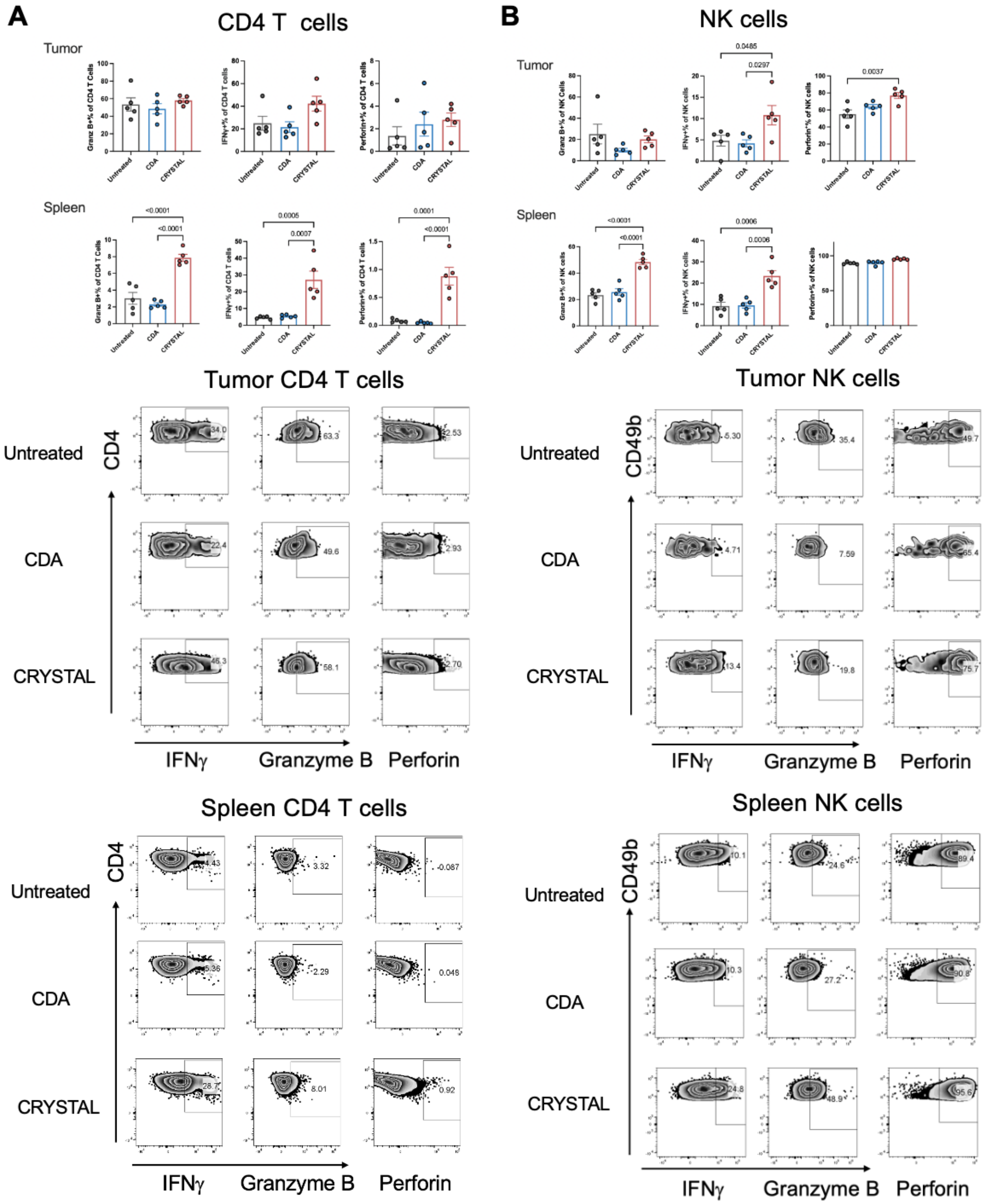

Fig. S27: **A**, **B**, *Ex vivo* activation of CD4$^+$ T cells (**A**) and NK cells (**B**) in tumor and spleen in the MMTV-PyMT model. The data represent the mean ± s.e.m. with n = 5 biologically independent samples and each dot represents an individual mouse (**A**, **B**, top). The data represent concatenated

populations with n = 5 biologically independent samples (**A**, **B**, bottom). The data were analyzed by one-way ANOVA, followed by Tukey's HSD multiple comparison *post hoc* test (**A, B**).

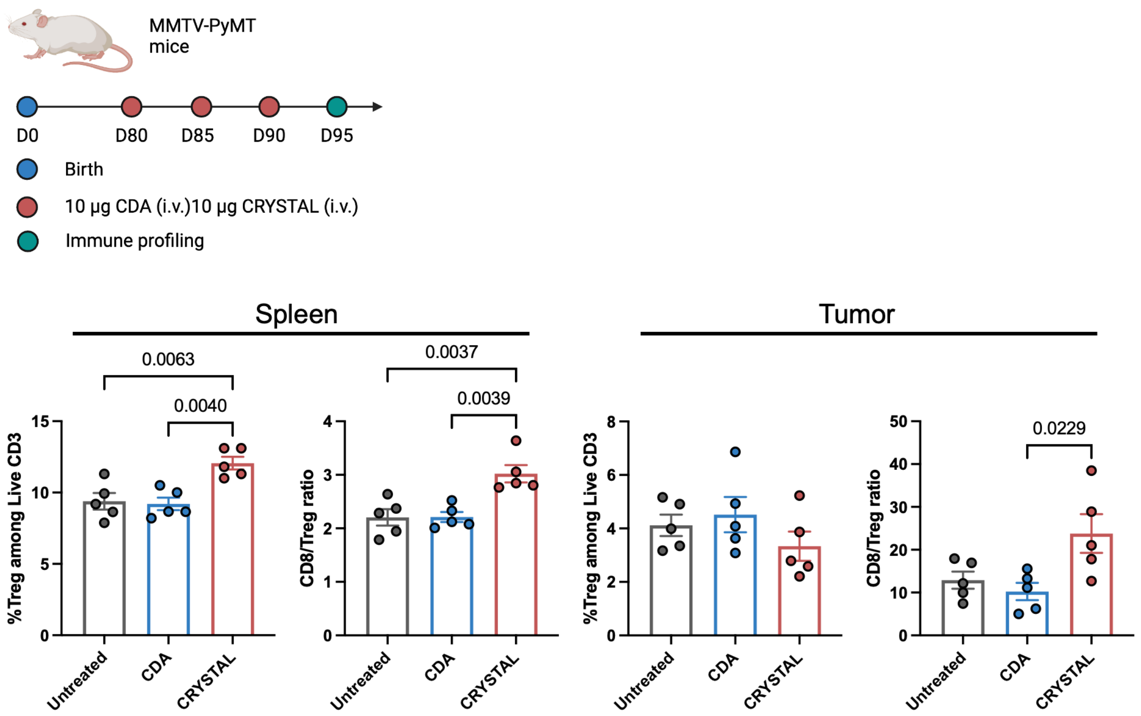

Fig. S28: MMTV-PyMT mice (D80 post birth) were enrolled and received IV injections of 10 μg CDA or CRYSTAL on D0, D5, and D10. Tumors and spleens were collected for immune profiling on D15. Frequency of Treg among live CD3 T cells and CD8 to Treg ratio were analyzed in spleen and tumor. The data represent the mean ± s.e.m. with n = 5 biologically independent samples and each dot represents an individual mouse. The data were analyzed by one-way ANOVA, followed by Tukey's HSD multiple comparison *post hoc* test.

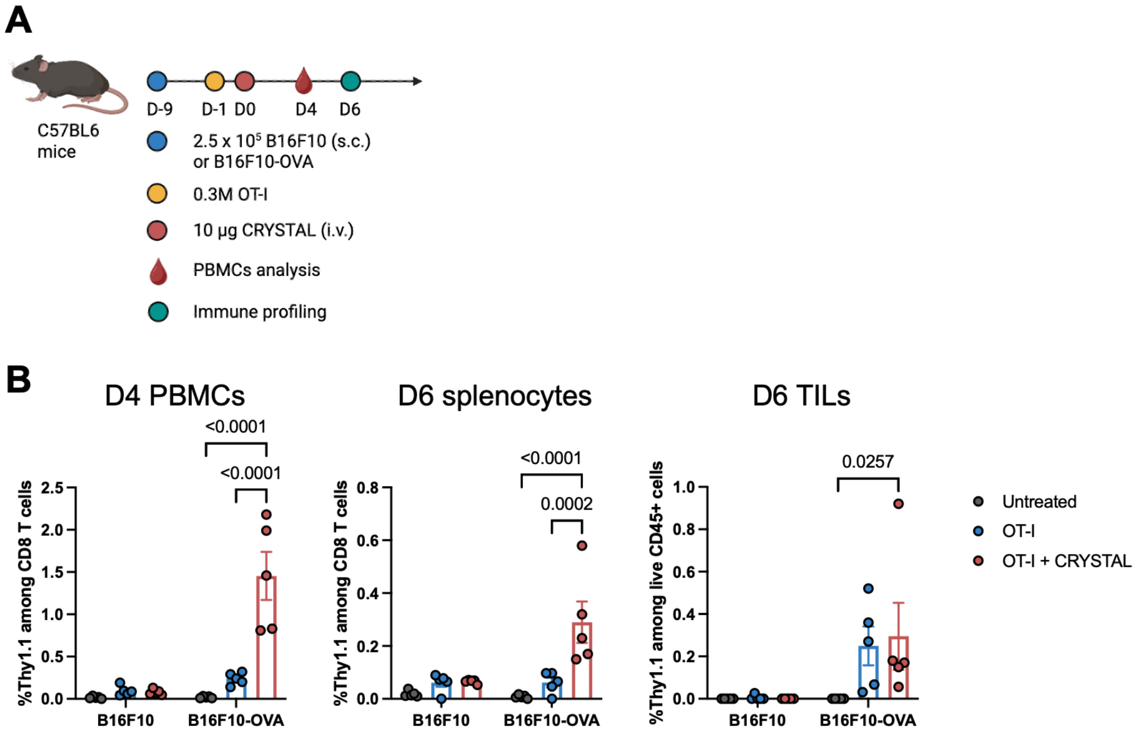

Fig. S29: *In vivo* expansion of antigen-specific T cells via CRYSTAL. 0.3 M freshly isolated OT-I cells were adoptively transferred to the mice bearing B16F10 or B16F10-OVA tumors. After CRYSTAL treatment, the expansion of OT-I cells was analyzed: treatment regimen (**A**), frequency of Thy1.1$^+$ OT-I in blood on D4 and in spleen and tumor on D6 (**B**). The data represent the mean ± s.e.m. with n = 5 biologically independent samples and each dot represents an individual mouse (**B**). The data were analyzed by two-way ANOVA, followed by Tukey's HSD multiple comparison *post hoc* test.

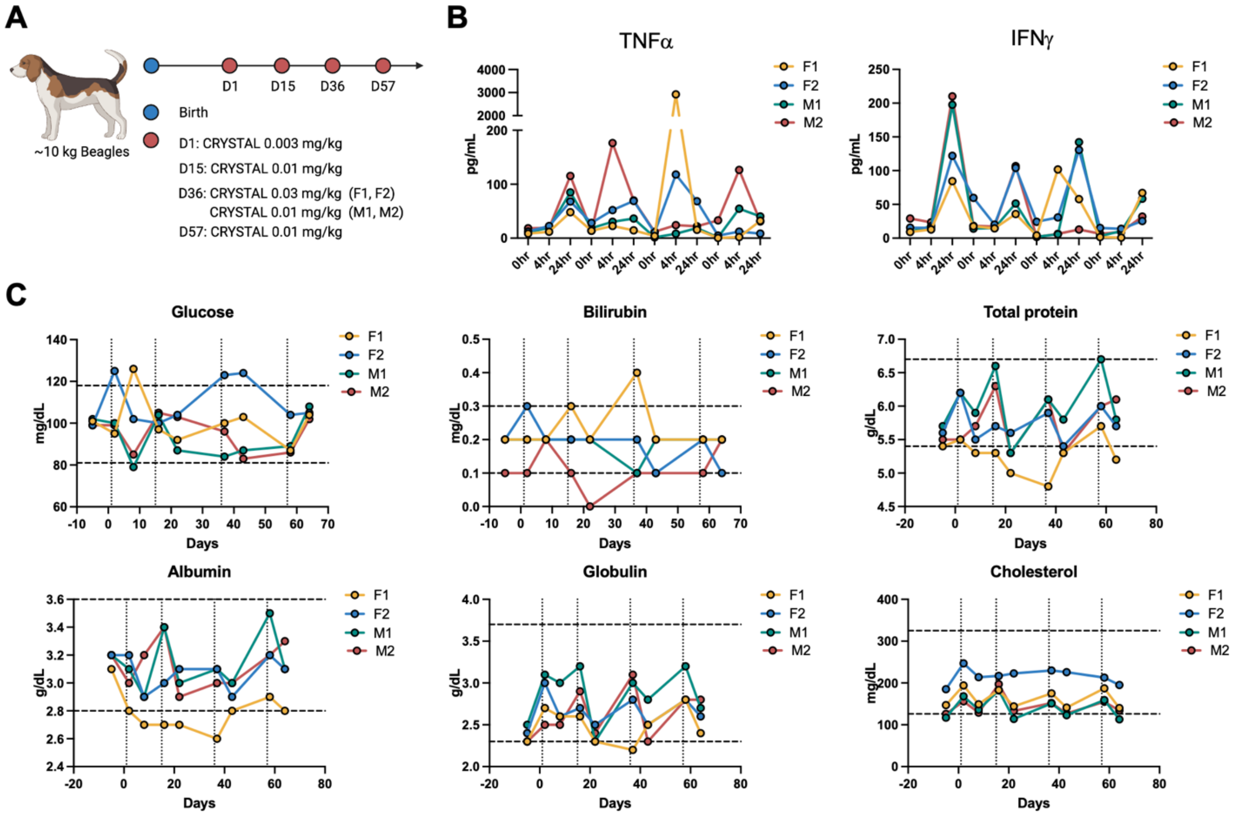

Fig. S30: **A**-**C**, Dose escalation trial of systemic CRYSTAL treatment in two female (F1, F2) and two male (M1, M2) naïve beagles. Dosing regimen was shown in (**A**). Investigation of systemic STING activation responses 4 hr and 24 hr after each dose with ELISA (TNF-α and IFN-γ) (values before each dose (0 hr) serve as the baseline) (**B**). Safety measurements of Glucose, Bilirubin, Total protein, Albumin, Globulin, and cholesterol levels of individual dogs (dashed horizontal lines indicate normal ranges, dotted vertical lines indicate the day of treatment) (**C**). Each line represents an individual animal, and each dot represents a measurement at a given time point.

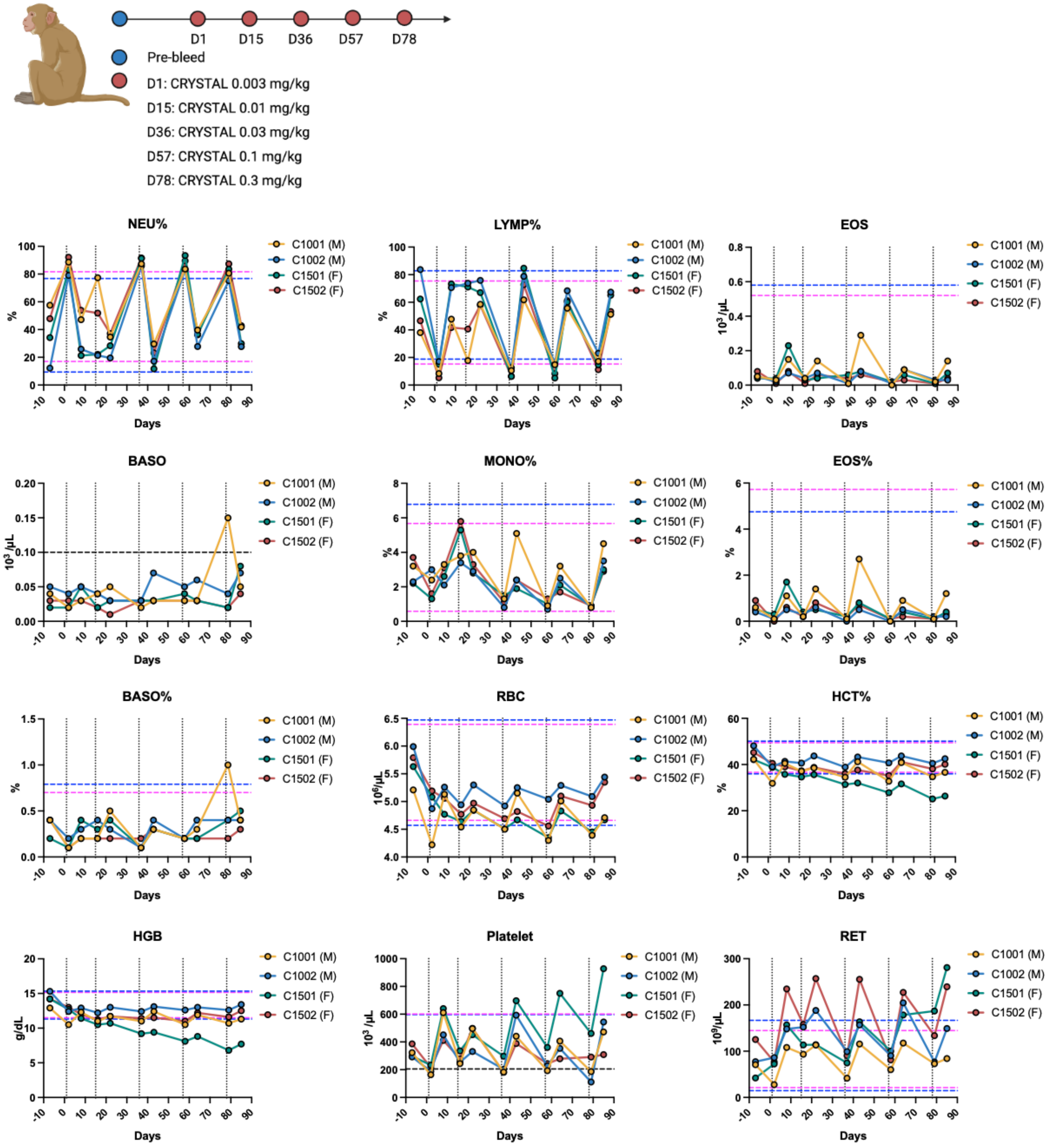

Fig. S31: Complete blood count (CBC) analysis in the dose escalation trial of systemic CRYSTAL treatment in two female (C1501, C1502) and two male (C1001, C1002) cynomolgus monkeys. Each line represents an individual animal, and each dot represents a measurement at a given time point. (dashed blue and pink horizontal lines indicate normal ranges for male and female NHPs respectively, dotted vertical lines indicate the day of treatment) (NEU: neutrophil, LYMP: lymphocytes, EOS: eosinophil, BASO: basophils, RBC: red blood cell, HCT: hematocrit, HGB: hemoglobin, RET: reticulocyte)

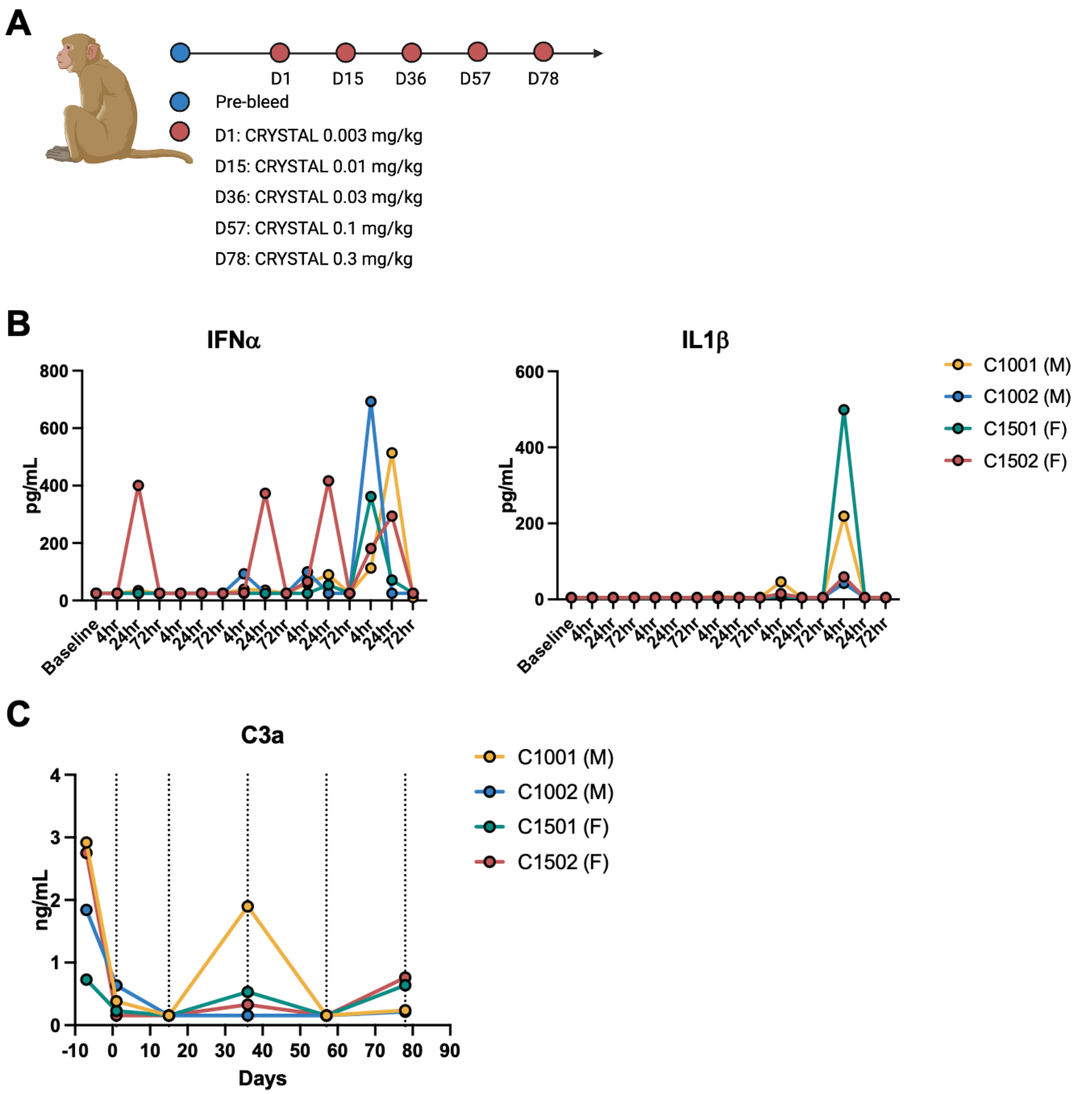

Fig. S32: A-C, Dose escalation trial of systemic CRYSTAL treatment in two female (C1501, C1502) and two male (C1001, C1002) cynomolgus monkeys. Dosing regimen was shown in (**A**). **B**, Investigation of systemic STING activation responses 4hr, 24hr, and 72hr after each dose with ELISA (IFN-α) and Meso Scale Discovery (MSD) assays (IL-1β) (values from pre-bleeding (D-7) serve as the baseline). **C,** C3a was measured via ELISA at the 0.5 hr timepoint during the IV infusion of each CRYSTAL dose. Each line represents an individual animal, and each dot represents a measurement at a given time point. (values from pre-bleeding (D-7) serve as the baseline, dotted vertical lines indicate the day of treatment)

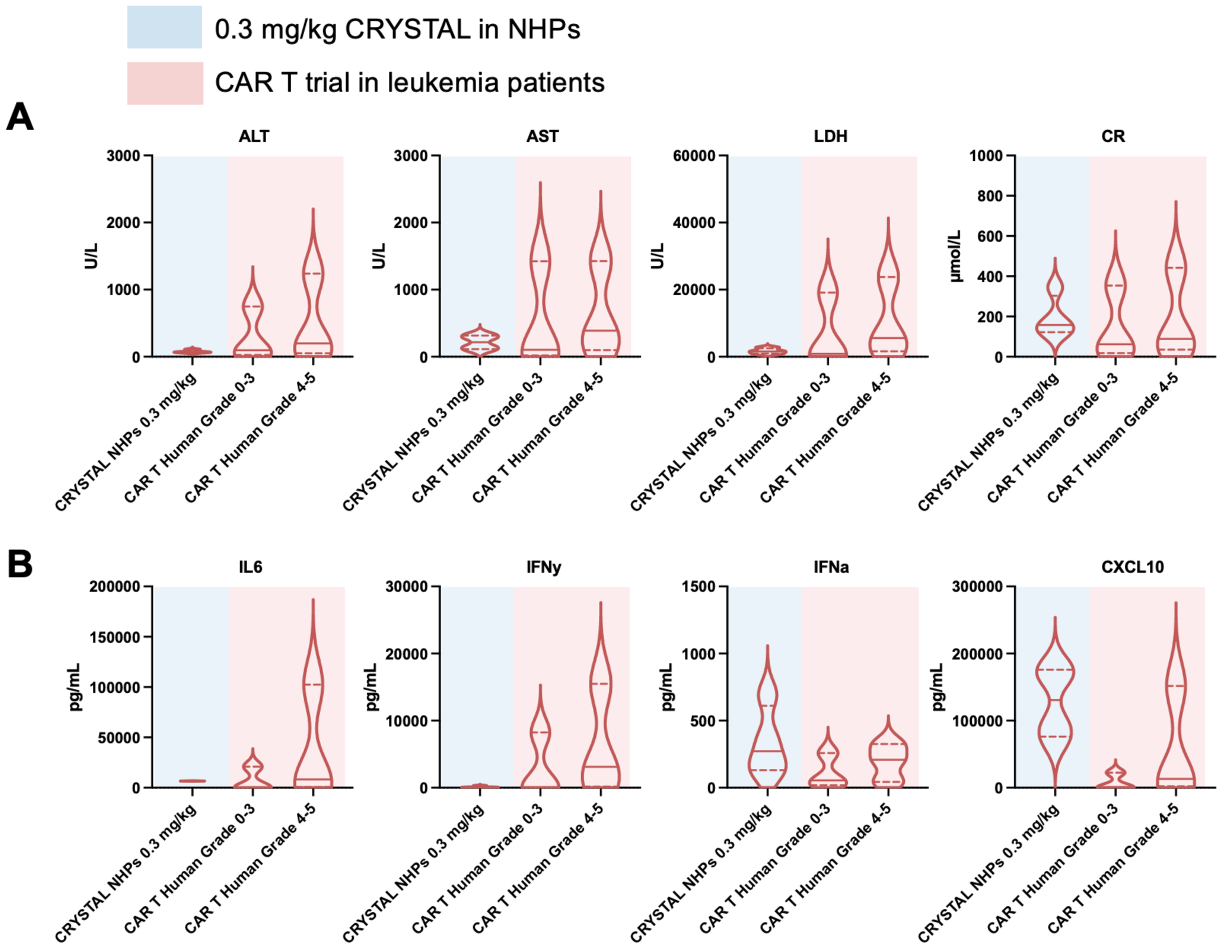

Fig. S33: Comparison of various chemistry and cytokine profiles of CRYSTAL (data represents peak values from 0.3 mg/kg CRYSTAL in NHPs, in blue shaded box) vs a reported CAR-T trial for acute lymphoblastic leukemia (data represents maximal, minimal, and median values from one-month-peak values in children and adults by grade (Grade 0-3: N = 37; Grade 4-5: N = 14), in red shaded box) (*47*).

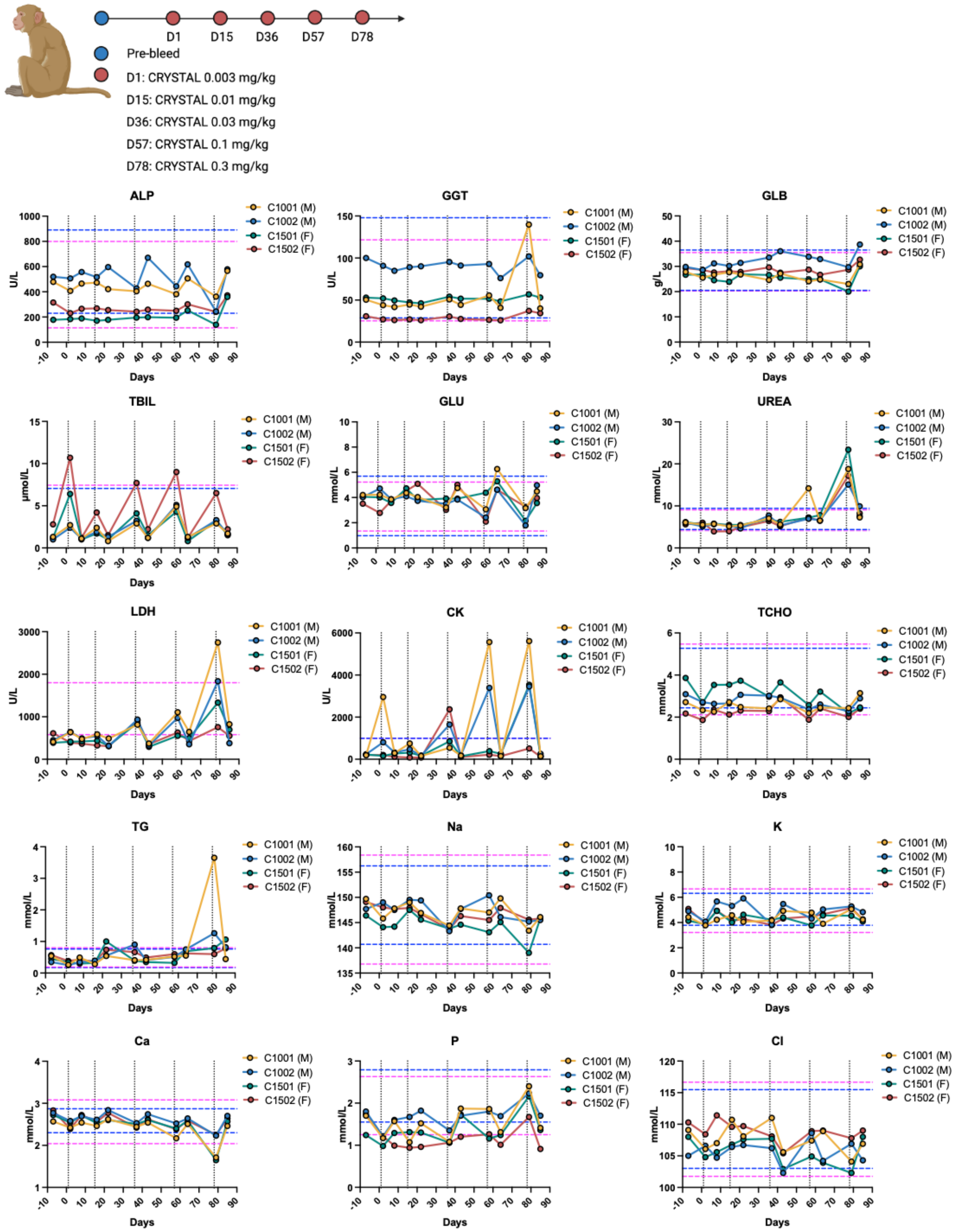

Fig. S34: Clinical chemistry panel in the dose escalation trial of systemic CRYSTAL treatment in two female (C1501, C1502) and two male (C1001, C1002) cynomolgus monkeys. Each line represents an individual animal, and each dot represents a measurement at a given time point. (dashed blue and pink horizontal lines indicate normal ranges for male and female NHPs respectively, dotted vertical lines indicate the day of treatment) (ALP: alkaline phosphatase, GGT: Gamma-glutamyl transferase, GLB: Globulin, TBIL: Total bilirubin, GLU: glucose, LDH: Lactate dehydrogenase, CK: Creatine kinase, TCHO: Total cholesterol, TG: Triglycerides.)

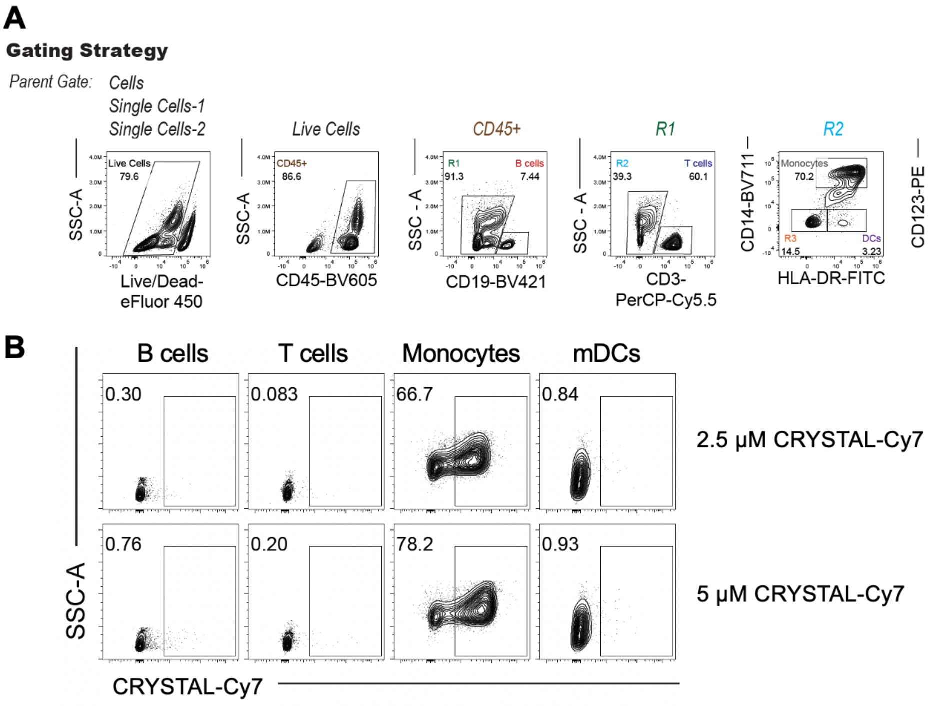

C

	Exon 3: 71	Exon 6: 230	Exon 6: 232	Exon 7: 293
WT	R	G	R	R
HAQ	H	A	R	Q
R232H	R	G	H	R
AQ	R	A	R	Q

Fig. S35: **A**, Gating strategy for identifying key immune subsets among human PBMCs. **B**, Representative flow plots of CRYSTAL-Cy7 uptake by B cells, T cells, monocytes, and mDCs. **C**, Table of human STING haplotypes with corresponding mutations highlighted in red.

A

Dose in mice (μg)	Dose in mice (mg/kg)	Equiv. Dose in NHPs (mg/kg)	Equiv. Dose in Dogs (mg/kg)	Equiv. Dose in Rabbits (mg/kg)
10	0.5	0.125	0.075	0.125
5	0.25	0.0625	0.0375	0.0625
2.5	0.125	0.0313	0.01875	0.03125
1	0.05	0.0125	0.0075	0.0125
0.5	0.025	0.00625	0.00375	0.00625
0.1	0.005	0.00125	0.00075	0.00125

B

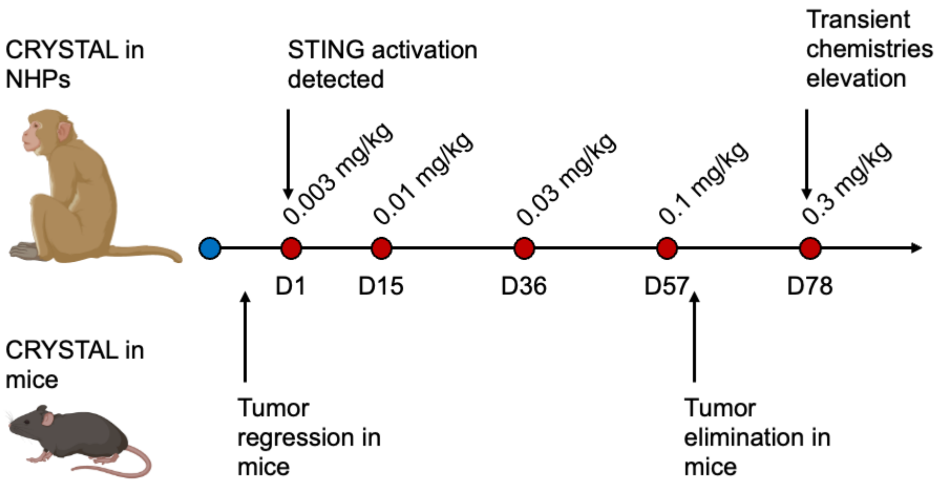

C

	Dose (μg)	Dose (mg/kg)	Models	Figures
Mice (~20g)	10	0.5	B16F10	Fig. 1I
			Large B16F10	Fig. 2A
			Large PyMT-MMTV	Fig. 2B
			NOOC1	Fig. 3F
	5	0.25	B16F10	Fig. 1I, 1J
			PDAC	Fig. S21
	2.5	0.125	B16F10	Fig. 1J
	1	0.05	B16F10	Fig. 1I, 1J
	0.5	0.025	B16F10	Fig. 1I
			NOOC1	Fig. S11
	0.1	0.005	B16F10	Fig. 1I
Rabbits (~2.9 kg)	500	0.17	VX2	Fig. 2F

Fig. S36: **A**, Doses used in mouse studies and the corresponding equivalent doses in rabbits, dogs, and NHPs calculated using standard body-surface-area (BSA) allometric scaling. Cross-species dose conversion was performed according to FDA-recommended BSA normalization using the following equation (*57*): $Dose_{species} = Dose_{mouse} \times K_m(mouse) / K_m(species)$. K_m(mouse)

= 3, K_m(rabbit) = 12, K_m(dog) = 20, K_m(NHPs) = 12. **B**, Schematic illustrating the dose ranges tested in NHPs and the aligned dose ranges in mice. **C**, Table summarizing all doses used across the murine and rabbit tumor models, along with the associated figure references. A broad range of doses was initially tested in aggressive B16F10 tumor–bearing mice to define the therapeutic window. Additional doses were subsequently selected to validate these findings across different tumor models.

Independent repeats of anti-tumor studies in mice

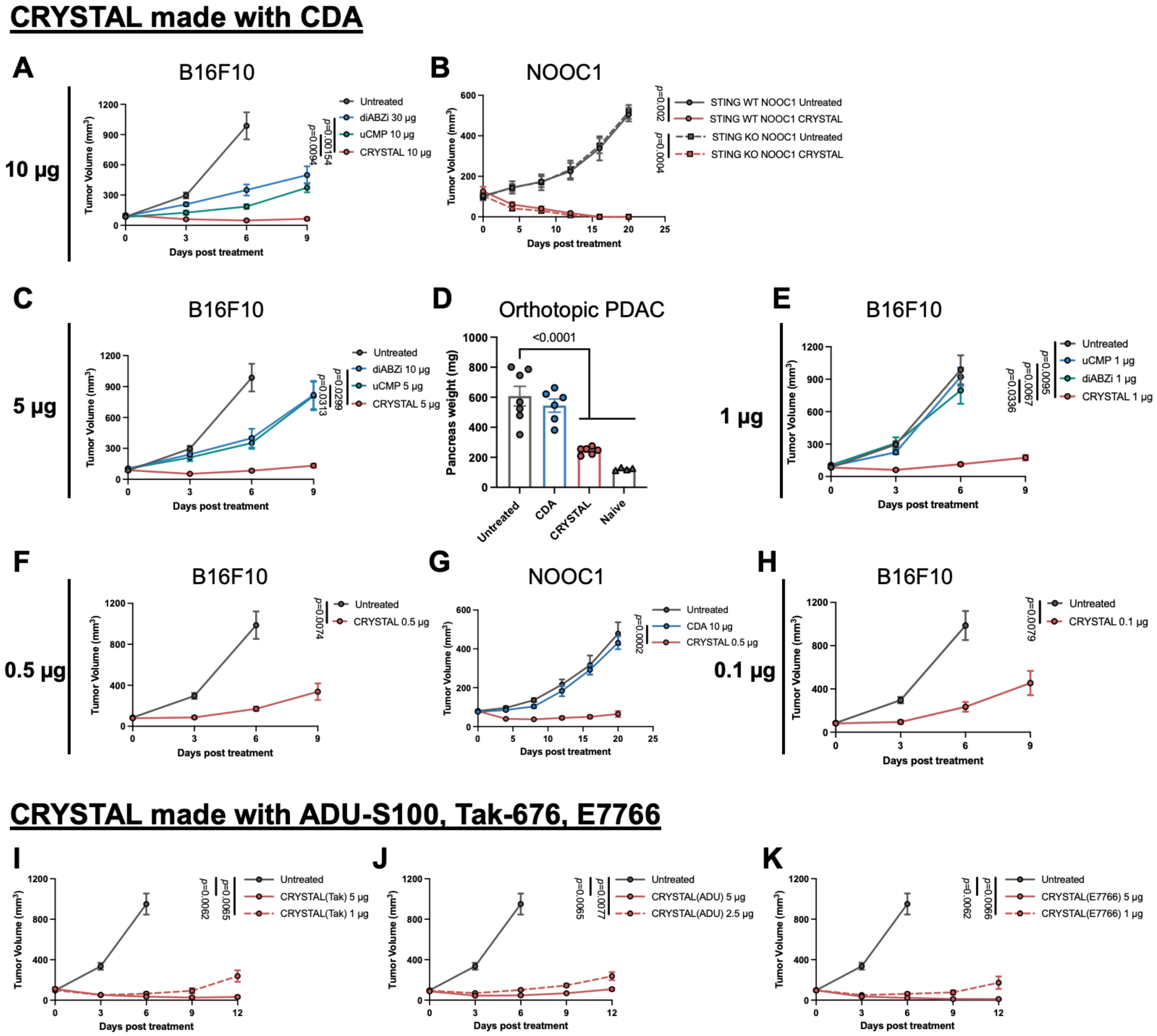

Fig. S37: Independent replicate data are shown for: Fig. 1I (**A**), Fig. 3F (**B**), Fig. 1I (**C**), Fig. S21 (**D**), Fig. 1I (**E**), Fig. 1I (**F**), Fig. S11 (**G**), Fig. 1I (**H**), Fig. 1J (**I-K**). For B16F10 tumor model, treatment with the indicated dosage of diABZi, uCMP, and CRYSTAL was performed on D0, 4, 8 via IV administration. The data represent the mean ± s.e.m. with n = 4 or 5 biologically independent samples. The data were analyzed by two-way ANOVA with Tukey's HSD multiple comparison *post hoc* test for tumor growth curve. For NOOC1 tumor model, treatment with the indicated dosage of CDA and CRYSTAL was performed on D0, 4, 8 via IV administration. The data represent the mean ± s.e.m. with n = 4 or 5 biologically independent samples. The data were analyzed by two-way ANOVA with Tukey's HSD multiple comparison *post hoc* test for tumor growth curve. For the Pan65671 orthotopic pancreatic cancer model, treatment with the indicated dosage of CDA and CRYSTAL was performed on D3, 7, 10 via IV administration. The pancreas weight on D14 were examined. The data represent the mean ± s.e.m. with n = 4-7 independent

biological samples and each dot represents an individual mouse. The data were analyzed by one-way ANOVA followed by Tukey's HSD multiple comparison *post hoc* test. For the investigation of antitumor efficacy of CRYSTALs loaded with various STING agonists, including Tak-676, ADU-S100, and E7766 in B16F10 tumor-bearing C57BL/6 mice, treatment with the indicated dosage was performed on D0, 4, 8 via IV administration. The data represent the mean ± s.e.m. with $n = 4$ biologically independent samples. The data were analyzed by two-way ANOVA with Tukey's HSD multiple comparison *post hoc* test for tumor growth curve.

Table S1: Parameters used in the coarse-grained computational modeling. Q, dz, and E_y depend on the length of histidine; the values listed are for His11.

Parameter	Value used in simulation
Q	$11e$
k_{medium}	$0.005\ k_BT - nm$
dx	$1.7nm$
dy	$1.6\ nm$
dz	$3.3\ nm$
E_y	$-4\ k_BT$
E_z	$-100\ k_BT$
E_{Mn}	$-8\ k_BT$

Table S2: Effect of His charge on crystal morphology.

Q	mean x dimension (nm)	mean y dimension (nm)	mean z dimension (nm)
$6e$	29.7 ± 1.62	9.45 ± 1.23	continuous growth
$11e$	25.7 ± 0.651	6.32 ± 0.511	continuous growth
$33e$	13.1 ± 0.410	0.735 ± 0.0894	continuous growth

Table S3: Power calculations based on pilot anti-tumor efficacy data from the B16F10 and MMTV-PyMT models. Effect sizes were estimated from pilot studies comparing CRYSTAL treatment with major control groups treated with free STING agonists (e.g., CDA and diABZi). These data suggested that group sizes of $n \geq 3$ mice per group would provide >90% statistical power for the indicated comparisons.

Model: MMTV-PyMT	Difference	Pooled SD	Cohen's d
10 µg CRYSTAL vs 30 µg diABZi	3761.71	589.5532	6.380612
Model: B16F10			
10 µg CRYSTAL vs 10 µg CDA	889.3638	156.8923	5.668627

Table S4: Antibodies used in the flow cytometry in mice samples.

Target	Fluorophore	Vendor	Catalog No.	Clone
CD45	FITC	BioLegend	157607	QA17A26
CD3	APC	BioLegend	100235	17A2
CD8	Pacific Blue	BioLegend	100728	53-6.7
CD4	BV605	BioLegend	100451	GK1.5
CD62L	BV785	BioLegend	104440	MEL-14
CD44	AF700	BioLegend	156009	NIM-R8
Ly6C	BV711	BioLegend	128037	HK1.4
CD49b	PE	BioLegend	103506	HMa2
F4/80	PE-Cy7	BioLegend	123113	BM8

CD206	BV650	BioLegend	141723	C068C2
MHC II	BV510	BioLegend	107635	M5/114.15.2
CD11b	PerCP-Cy5.5	BioLegend	101227	M1/70
CD11c	BV750	BioLegend	117357	N418
Ly6G	Spark Blue 550	BioLegend	127663	1A8
XCR1	Pacific Blue	BioLegend	148241	ZET
Thy1.1	BV711	BioLegend	202539	OX-7
CD172a (SIRPα)	RB744	Fisher Scientific	BDB757596	P84
FOXP3	PE-Cy5.5	Fisher Scientific	50-112-3557	FJK-16s
CXCR3	BV421	BioLegend	126521	CXCR3-173
CD4	BV510	BioLegend	100449	GK1.5
CD62L	BV605	BioLegend	104437	MEL-14
CX3CR1	BV711	BioLegend	149031	SA011F11
PD-1	BV785	BioLegend	135225	29F.1A12
TCF1	PE	Cell Signaling Technology	14456S	C63D9
CD44	PE-Cy5	BioLegend	103009	IM7
Sca-1	PE-Cy7	BioLegend	108114	D7
Ki-67	Alexa Fluor 647	BioLegend	652408	16A8
CD3	Alexa Fluor 700	BioLegend	100215	17A2
CD45	BV605	BioLegend	103155	30-F11
IFN-γ	BV785	BioLegend	505837	XMG1.2
Granzyme B	PE-Cy7	Fisher Scientific	50-245-757	NGZB
Perforin	APC	BioLegend	154403	S16009B

Table S5: Antibodies used in the flow cytometry in human samples.

Target	Fluorophore	Vendor	Catalog No.	Clone
CD45	BV605	BioLegend	304042	HI30
CD19	BV421	BioLegend	302234	HIB19
CD3	PerCP-Cy5.5	BioLegend	344807	SK7
HLA-DR	FITC	BD	BDB555560	TU36
CD11c	APC	BioLegend	301614	3.9
CD56	PE-Cy7	BioLegend	362509	NCAM

Table S6: Age and sex information for human participants.

Sample number	Age	Sex
1	75	M
2	69	M
3	43	F

4	62	F
5	54	M
6	42	F
7	74	M
8	67	M
9	56	F
10	69	F
11	61	M
12	61	M
13	50	M
14	47	M
15	64	M
16	76	M
17	53	F
18	69	M
19	65	F
20	45	F
21	73	F
22	59	M
23	62	M
24	62	F
25	58	M
26	72	M
27	58	F
28	62	M
29	81	M

Movie S1: Video of CDA/Mn/His11 growth during the MC simulations.
Movie S2: Video of CDA/Mn/His6 growth during the MC simulations.
Movie S3: Video of CDA/Mn/His33 growth during the MC simulations.

Reference

57. A. B. Nair, S. Jacob, A simple practice guide for dose conversion between animals and human. *J Basic Clin Pharm* **7**, 27-31 (2016).

References and Notes

1. K. L. Bren, R. Eisenberg, H. B. Gray, Discovery of the magnetic behavior of hemoglobin: A beginning of bioinorganic chemistry. *Proc. Natl. Acad. Sci. U.S.A.* **112**, 13123–13127 (2015). doi:10.1073/pnas.1515704112 Medline

2. A. La Fontaine, A. Zavgorodniy, H. Liu, R. Zheng, M. Swain, J. Cairney, Atomic-scale compositional mapping reveals Mg-rich amorphous calcium phosphate in human dental enamel. *Sci. Adv.* **2**, e1601145 (2016). doi:10.1126/sciadv.1601145 Medline

3. H. Zhao, S. Liu, Y. Wei, Y. Yue, M. Gao, Y. Li, X. Zeng, X. Deng, N. A. Kotov, L. Guo, L. Jiang, Multiscale engineered artificial tooth enamel. *Science* **375**, 551–556 (2022). doi:10.1126/science.abj3343 Medline

4. K. Pounot, G. W. Grime, A. Longo, M. Zamponi, D. Noferini, V. Cristiglio, T. Seydel, E. F. Garman, M. Weik, V. Foderà, G. Schirò, Zinc determines dynamical properties and aggregation kinetics of human insulin. *Biophys. J.* **120**, 886–898 (2021). doi:10.1016/j.bpj.2020.11.2280 Medline

5. C.-L. Yang, L.-N. Wang, P. Yin, J. Liu, M.-X. Chen, Q.-Q. Yan, Z.-S. Wang, S.-L. Xu, S.-Q. Chu, C. Cui, H. Ju, J. Zhu, Y. Lin, J. Shui, H.-W. Liang, Sulfur-anchoring synthesis of platinum intermetallic nanoparticle catalysts for fuel cells. *Science* **374**, 459–464 (2021). doi:10.1126/science.abj9980 Medline

6. B. Peng, Z. Liu, L. Sementa, Q. Jia, Q. Sun, C. U. Segre, E. Liu, M. Xu, Y.-H. Tsai, X. Yan, Z. Zhao, J. Huang, X. Pan, X. Duan, A. Fortunelli, Y. Huang, Embedded oxide clusters stabilize sub-2 nm Pt nanoparticles for highly durable fuel cells. *Nat. Catal.* **7**, 818–828 (2024). doi:10.1038/s41929-024-01180-x

7. P. Kumar, T. Vo, M. Cha, A. Visheratina, J.-Y. Kim, W. Xu, J. Schwartz, A. Simon, D. Katz, V. P. Nicu, E. Marino, W. J. Choi, M. Veksler, S. Chen, C. Murray, R. Hovden, S. Glotzer, N. A. Kotov, Photonically active bowtie nanoassemblies with chirality continuum. *Nature* **615**, 418–424 (2023). doi:10.1038/s41586-023-05733-1 Medline

8. J. Wu, L. Sun, X. Chen, F. Du, H. Shi, C. Chen, Z. J. Chen, Cyclic GMP-AMP is an endogenous second messenger in innate immune signaling by cytosolic DNA. *Science* **339**, 826–830 (2013). doi:10.1126/science.1229963 Medline

9. L. Sun, J. Wu, F. Du, X. Chen, Z. J. Chen, Cyclic GMP-AMP synthase is a cytosolic DNA sensor that activates the type I interferon pathway. *Science* **339**, 786–791 (2013). doi:10.1126/science.1232458 Medline

10. N. Samson, A. Ablasser, The cGAS–STING pathway and cancer. *Nat. Cancer* **3**, 1452–1463 (2022). doi:10.1038/s43018-022-00468-w Medline

11. L. Corrales, L. H. Glickman, S. M. McWhirter, D. B. Kanne, K. E. Sivick, G. E. Katibah, S.-R. Woo, E. Lemmens, T. Banda, J. J. Leong, K. Metchette, T. W. Dubensky Jr., T. F. Gajewski, Direct activation of STING in the tumor microenvironment leads to potent and systemic tumor regression and immunity. *Cell Rep.* **11**, 1018–1030 (2015). doi:10.1016/j.celrep.2015.04.031 Medline

12. F. Meric-Bernstam, R. F. Sweis, S. Kasper, O. Hamid, S. Bhatia, R. Dummer, A. Stradella, G. V. Long, A. Spreafico, T. Shimizu, N. Steeghs, J. J. Luke, S. M. McWhirter, T.

Müller, N. Nair, N. Lewis, X. Chen, A. Bean, L. Kattenhorn, M. Pelletier, S. Sandhu, Combination of the STING agonist MIW815 (ADU-S100) and PD-1 inhibitor spartalizumab in advanced/metastatic solid tumors or lymphomas: An open-label, multicenter, phase Ib study. *Clin. Cancer Res.* **29**, 110–121 (2023). doi:10.1158/1078-0432.CCR-22-2235 Medline

13. J. M. Ramanjulu, G. S. Pesiridis, J. Yang, N. Concha, R. Singhaus, S.-Y. Zhang, J.-L. Tran, P. Moore, S. Lehmann, H. C. Eberl, M. Muelbaier, J. L. Schneck, J. Clemens, M. Adam, J. Mehlmann, J. Romano, A. Morales, J. Kang, L. Leister, T. L. Graybill, A. K. Charnley, G. Ye, N. Nevins, K. Behnia, A. I. Wolf, V. Kasparcova, K. Nurse, L. Wang, A. C. Puhl, Y. Li, M. Klein, C. B. Hopson, J. Guss, M. Bantscheff, G. Bergamini, M. A. Reilly, Y. Lian, K. J. Duffy, J. Adams, K. P. Foley, P. J. Gough, R. W. Marquis, J. Smothers, A. Hoos, J. Bertin, Design of amidobenzimidazole STING receptor agonists with systemic activity. *Nature* **564**, 439–443 (2018). doi:10.1038/s41586-018-0705-y Medline

14. E. Carideo Cunniff, Y. Sato, D. Mai, V. A. Appleman, S. Iwasaki, V. Kolev, A. Matsuda, J. Shi, M. Mochizuki, M. Yoshikawa, J. Huang, L. Shen, S. Haridas, V. Shinde, C. Gemski, E. R. Roberts, O. Ghasemi, H. Bazzazi, S. Menon, T. Traore, P. Shi, T. D. Thelen, J. Conlon, A. O. Abu-Yousif, C. Arendt, M. H. Shaw, M. Okaniwa, TAK-676: A Novel stimulator of interferon genes (STING) agonist promoting durable IFN-dependent antitumor immunity in preclinical studies. *Cancer Res. Commun.* **2**, 489–502 (2022). doi:10.1158/2767-9764.CRC-21-0161 Medline

15. E. N. Chin, C. Yu, V. F. Vartabedian, Y. Jia, M. Kumar, A. M. Gamo, W. Vernier, S. H. Ali, M. Kissai, D. C. Lazar, N. Nguyen, L. E. Pereira, B. Benish, A. K. Woods, S. B. Joseph, A. Chu, K. A. Johnson, P. N. Sander, F. Martínez-Peña, E. N. Hampton, T. S. Young, D. W. Wolan, A. K. Chatterjee, P. G. Schultz, H. M. Petrassi, J. R. Teijaro, L. L. Lairson, Antitumor activity of a systemic STING-activating non-nucleotide cGAMP mimetic. *Science* **369**, 993–999 (2020). doi:10.1126/science.abb4255 Medline

16. K. Yang, W. Han, X. Jiang, A. Piffko, J. Bugno, C. Han, S. Li, H. Liang, Z. Xu, W. Zheng, L. Wang, J. Wang, X. Huang, J. P. Y. Ting, Y.-X. Fu, W. Lin, R. R. Weichselbaum, Zinc cyclic di-AMP nanoparticles target and suppress tumours via endothelial STING activation and tumour-associated macrophage reinvigoration. *Nat. Nanotechnol.* **17**, 1322–1331 (2022). doi:10.1038/s41565-022-01225-x Medline

17. D. Shae, K. W. Becker, P. Christov, D. S. Yun, A. K. R. Lytton-Jean, S. Sevimli, M. Ascano, M. Kelley, D. B. Johnson, J. M. Balko, J. T. Wilson, Endosomolytic polymersomes increase the activity of cyclic dinucleotide STING agonists to enhance cancer immunotherapy. *Nat. Nanotechnol.* **14**, 269–278 (2019). doi:10.1038/s41565-018-0342-5 Medline

18. P. Dosta, A. M. Cryer, M. Z. Dion, T. Shiraishi, S. P. Langston, D. Lok, J. Wang, S. Harrison, T. Hatten, M. L. Ganno, V. A. Appleman, G. M. Taboada, N. Puigmal, S. Ferber, S. Kalash, M. Prado, A. L. Rodríguez, W. S. Kamoun, A. O. Abu-Yousif, N. Artzi, Investigation of the enhanced antitumour potency of STING agonist after conjugation to polymer nanoparticles. *Nat. Nanotechnol.* **18**, 1351–1363 (2023). doi:10.1038/s41565-023-01447-7 Medline

19. E. L. Dane, A. Belessiotis-Richards, C. Backlund, J. Wang, K. Hidaka, L. E. Milling, S. Bhagchandani, M. B. Melo, S. Wu, N. Li, N. Donahue, K. Ni, L. Ma, M. Okaniwa, M. M. Stevens, A. Alexander-Katz, D. J. Irvine, STING agonist delivery by tumour-penetrating PEG-lipid nanodiscs primes robust anticancer immunity. *Nat. Mater.* **21**, 710–720 (2022). doi:10.1038/s41563-022-01251-z Medline

20. X. Sun, Y. Zhang, J. Li, K. S. Park, K. Han, X. Zhou, Y. Xu, J. Nam, J. Xu, X. Shi, L. Wei, Y. L. Lei, J. J. Moon, Amplifying STING activation by cyclic dinucleotide-manganese particles for local and systemic cancer metalloimmunotherapy. *Nat. Nanotechnol.* **16**, 1260–1270 (2021). doi:10.1038/s41565-021-00962-9 Medline

21. S. Li, M. Luo, Z. Wang, Q. Feng, J. Wilhelm, X. Wang, W. Li, J. Wang, A. Cholka, Y. X. Fu, B. D. Sumer, H. Yu, J. Gao, Prolonged activation of innate immune pathways by a polyvalent STING agonist. *Nat. Biomed. Eng.* **5**, 455–466 (2021). doi:10.1038/s41551-020-00675-9 Medline

22. J. Lu, W. Wu, F. M. Colombari, A. Jawaid, B. Seymour, K. Whisnant, X. Zhong, W. Choi, N. Chalmpes, J. Lahann, R. A. Vaia, A. F. de Moura, D. Nepal, N. A. Kotov, Nano-achiral complex composites for extreme polarization optics. *Nature* **630**, 860–865 (2024). doi:10.1038/s41586-024-07455-4 Medline

23. W. Jiang, Z. B. Qu, P. Kumar, D. Vecchio, Y. Wang, Y. Ma, J. H. Bahng, K. Bernardino, W. R. Gomes, F. M. Colombari, A. Lozada-Blanco, M. Veksler, E. Marino, A. Simon, C. Murray, S. R. Muniz, A. F. de Moura, N. A. Kotov, Emergence of complexity in hierarchically organized chiral particles. *Science* **368**, 642–648 (2020). doi:10.1126/science.aaz7949 Medline

24. Q. Cheng, T. Wei, L. Farbiak, L. T. Johnson, S. A. Dilliard, D. J. Siegwart, Selective organ targeting (SORT) nanoparticles for tissue-specific mRNA delivery and CRISPR–Cas gene editing. *Nat. Nanotechnol.* **15**, 313–320 (2020). doi:10.1038/s41565-020-0669-6 Medline

25. Y. Sun, S. Chatterjee, X. Lian, Z. Traylor, S. R. Sattiraju, Y. Xiao, S. A. Dilliard, Y.-C. Sung, M. Kim, S. M. Lee, S. Moore, X. Wang, D. Zhang, S. Wu, P. Basak, J. Wang, J. Liu, R. J. Mann, D. F. LePage, W. Jiang, S. Abid, M. Hennig, A. Martinez, B. A. Wustman, D. J. Lockhart, R. Jain, R. A. Conlon, M. L. Drumm, C. A. Hodges, D. J. Siegwart, In vivo editing of lung stem cells for durable gene correction in mice. *Science* **384**, 1196–1202 (2024). doi:10.1126/science.adk9428 Medline

26. M. Rabinovitch, Professional and non-professional phagocytes: An introduction. *Trends Cell Biol.* **5**, 85–87 (1995). doi:10.1016/S0962-8924(00)88955-2 Medline

27. B. C. Lo, I. Kryczek, J. Yu, L. Vatan, R. Caruso, M. Matsumoto, Y. Sato, M. H. Shaw, N. Inohara, Y. Xie, Y. L. Lei, W. Zou, G. Núñez, Microbiota-dependent activation of $CD4^+$ T cells induces CTLA-4 blockade–associated colitis via Fcγ receptors. *Science* **383**, 62–70 (2024). doi:10.1126/science.adh8342 Medline

28. D.-S. Kim, A. Endo, F. G. Fang, K.-C. Huang, X. Bao, H. W. Choi, U. Majumder, Y. Y. Shen, S. Mathieu, X. Zhu, K. Sanders, T. Noland, M.-H. Hao, Y. Chen, J. Y. Wang, S. Yasui, K. TenDyke, J. Wu, C. Ingersoll, K. A. Loiacono, J. E. Hutz, N. Sarwar, E7766, a macrocycle-bridged stimulator of interferon genes (STING) agonist with potent pan-

genotypic activity. *ChemMedChem* **16**, 1740–1743 (2021). doi:10.1002/cmdc.202100068 Medline

29. L. Li, Q. Yin, P. Kuss, Z. Maliga, J. L. Millán, H. Wu, T. J. Mitchison, Hydrolysis of 2′3′-cGAMP by ENPP1 and design of nonhydrolyzable analogs. *Nat. Chem. Biol.* **10**, 1043–1048 (2014). doi:10.1038/nchembio.1661 Medline

30. K. D. Moynihan, C. F. Opel, G. L. Szeto, A. Tzeng, E. F. Zhu, J. M. Engreitz, R. T. Williams, K. Rakhra, M. H. Zhang, A. M. Rothschilds, S. Kumari, R. L. Kelly, B. H. Kwan, W. Abraham, K. Hu, N. K. Mehta, M. J. Kauke, H. Suh, J. R. Cochran, D. A. Lauffenburger, K. D. Wittrup, D. J. Irvine, Eradication of large established tumors in mice by combination immunotherapy that engages innate and adaptive immune responses. *Nat. Med.* **22**, 1402–1410 (2016). doi:10.1038/nm.4200 Medline

31. H. Zheng, F. Zhang, W. Monsky, H. Ji, W. Yang, X. Yang, Interventional optical imaging-monitored synergistic effect of radio-frequency hyperthermia and oncolytic immunotherapy. *Front. Oncol.* **11**, 821838 (2022). doi:10.3389/fonc.2021.821838 Medline

32. M. K. Thomsen, R. Nandakumar, D. Stadler, A. Malo, R. M. Valls, F. Wang, L. S. Reinert, F. Dagnaes-Hansen, A. K. Hollensen, J. G. Mikkelsen, U. Protzer, S. R. Paludan, Lack of immunological DNA sensing in hepatocytes facilitates hepatitis B virus infection. *Hepatology* **64**, 746–759 (2016). doi:10.1002/hep.28685 Medline

33. M. Wehbe, L. Wang-Bishop, K. W. Becker, D. Shae, J. J. Baljon, X. He, P. Christov, K. L. Boyd, J. M. Balko, J. T. Wilson, Nanoparticle delivery improves the pharmacokinetic properties of cyclic dinucleotide STING agonists to open a therapeutic window for intravenous administration. *J. Control. Release* **330**, 1118–1129 (2021). doi:10.1016/j.jconrel.2020.11.017 Medline

34. B. Jneid, A. Bochnakian, C. Hoffmann, F. Delisle, E. Djacoto, P. Sirven, J. Denizeau, C. Sedlik, Y. Gerber-Ferder, F. Fiore, R. Akyol, C. Brousse, R. Kramer, I. Walters, S. Carlioz, H. Salmon, B. Malissen, M. Dalod, E. Piaggio, N. Manel, Selective STING stimulation in dendritic cells primes antitumor T cell responses. *Sci. Immunol.* **8**, eabn6612 (2023). doi:10.1126/sciimmunol.abn6612 Medline

35. J. Wang, S. Li, M. Wang, X. Wang, S. Chen, Z. Sun, X. Ren, G. Huang, B. D. Sumer, N. Yan, Y.-X. Fu, J. Gao, STING licensing of type I dendritic cells potentiates antitumor immunity. *Sci. Immunol.* **9**, eadj3945 (2024). doi:10.1126/sciimmunol.adj3945 Medline

36. Z. Zou, J. Shen, D. Xue, H. Li, L. Xu, W. Cao, W. Wang, Y.-X. Fu, H. Peng, Anti-PD-1 cis-delivery of low-affinity IL-12 activates intratumoral $CD8^{+}$T cells for systemic antitumor responses. *Nat. Commun.* **15**, 4701 (2024). doi:10.1038/s41467-024-49034-1 Medline

37. K. H. Dreaden, S.-P. Pearson, P. S. Gurel, R. G. Newman, Y. Zhao, C. Wang, J. F. Heiber, S. S. Donatelli, J. Chamoun, M. M. Whitmore, Interleukin-18 (IL-18) engineered for half-life extension and resistance to IL-18 binding protein (IL-18BP) to enhance anti-cancer therapeutic potential. *Cytokine* **193**, 156979 (2025). doi:10.1016/j.cyto.2025.156979 Medline

38. M. Akrami, R. Menzies, K. Chamoto, M. Miyajima, R. Suzuki, H. Sato, A. Nishii, M. Tomura, S. Fagarasan, T. Honjo, Circulation of gut-preactivated naïve $CD8^{+}$ T cells

enhances antitumor immunity in B cell-defective mice. *Proc. Natl. Acad. Sci. U.S.A.* **117**, 23674–23683 (2020). doi:10.1073/pnas.2010981117 Medline

39. C. Tsui, L. Kretschmer, S. Rapelius, S. S. Gabriel, D. Chisanga, K. Knöpper, D. T. Utzschneider, S. Nüssing, Y. Liao, T. Mason, S. V. Torres, S. A. Wilcox, K. Kanev, S. Jarosch, J. Leube, S. L. Nutt, D. Zehn, I. A. Parish, W. Kastenmüller, W. Shi, V. R. Buchholz, A. Kallies, MYB orchestrates T cell exhaustion and response to checkpoint inhibition. *Nature* **609**, 354–360 (2022). doi:10.1038/s41586-022-05105-1 Medline

40. K. A. Tran, E. Pernet, M. Sadeghi, J. Downey, J. Chronopoulos, E. Lapshina, O. Tsai, E. Kaufmann, J. Ding, M. Divangahi, BCG immunization induces CX3CR1^{hi} effector memory T cells to provide cross-protection via IFN-γ-mediated trained immunity. *Nat. Immunol.* **25**, 418–431 (2024). doi:10.1038/s41590-023-01739-z Medline

41. V. Verma, N. Jafarzadeh, S. Boi, S. Kundu, Z. Jiang, Y. Fan, J. Lopez, R. Nandre, P. Zeng, F. Alolaqi, S. Ahmad, P. Gaur, S. T. Barry, V. E. Valge-Archer, P. D. Smith, J. Banchereau, M. Mkrtichyan, B. Youngblood, P. C. Rodriguez, S. Gupta, S. N. Khleif, MEK inhibition reprograms CD8$^+$ T lymphocytes into memory stem cells with potent antitumor effects. *Nat. Immunol.* **22**, 53–66 (2021). doi:10.1038/s41590-020-00818-9 Medline

42. C. Sugimoto, A. Hasegawa, Y. Saito, Y. Fukuyo, K. B. Chiu, Y. Cai, M. W. Breed, K. Mori, C. J. Roy, A. A. Lackner, W.-K. Kim, E. S. Didier, M. J. Kuroda, Differentiation kinetics of blood monocytes and dendritic cells in macaques: Insights to understanding human myeloid cell development. *J. Immunol.* **195**, 1774–1781 (2015). doi:10.4049/jimmunol.1500522 Medline

43. S. F. Bakhoum, B. Ngo, A. M. Laughney, J.-A. Cavallo, C. J. Murphy, P. Ly, P. Shah, R. K. Sriram, T. B. K. Watkins, N. K. Taunk, M. Duran, C. Pauli, C. Shaw, K. Chadalavada, V. K. Rajasekhar, G. Genovese, S. Venkatesan, N. J. Birkbak, N. McGranahan, M. Lundquist, Q. LaPlant, J. H. Healey, O. Elemento, C. H. Chung, N. Y. Lee, M. Imielenski, G. Nanjangud, D. Pe'er, D. W. Cleveland, S. N. Powell, J. Lammerding, C. Swanton, L. C. Cantley, Chromosomal instability drives metastasis through a cytosolic DNA response. *Nature* **553**, 467–472 (2018). doi:10.1038/nature25432 Medline

44. A. Ablasser, S. Hur, Regulation of cGAS- and RLR-mediated immunity to nucleic acids. *Nat. Immunol.* **21**, 17–29 (2020). doi:10.1038/s41590-019-0556-1 Medline

45. A. A. Stegelmeier, M. Darzianiazizi, K. Hanada, S. Sharif, S. K. Wootton, B. W. Bridle, K. Karimi, Type I interferon-mediated regulation of antiviral capabilities of neutrophils. *Int. J. Mol. Sci.* **22**, 4726 (2021). doi:10.3390/ijms22094726 Medline

46. E. C. Morris, S. S. Neelapu, T. Giavridis, M. Sadelain, Cytokine release syndrome and associated neurotoxicity in cancer immunotherapy. *Nat. Rev. Immunol.* **22**, 85–96 (2022). doi:10.1038/s41577-021-00547-6 Medline

47. D. T. Teachey, S. F. Lacey, P. A. Shaw, J. J. Melenhorst, S. L. Maude, N. Frey, E. Pequignot, V. E. Gonzalez, F. Chen, J. Finklestein, D. M. Barrett, S. L. Weiss, J. C. Fitzgerald, R. A. Berg, R. Aplenc, C. Callahan, S. R. Rheingold, Z. Zheng, S. Rose-John, J. C. White, F. Nazimuddin, G. Wertheim, B. L. Levine, C. H. June, D. L. Porter, S. A. Grupp, Identification of predictive biomarkers for cytokine release syndrome after

chimeric antigen receptor T-cell therapy for acute lymphoblastic leukemia. *Cancer Discov.* **6**, 664–679 (2016). doi:10.1158/2159-8290.CD-16-0040 Medline

48. G. Yi, V. P. Brendel, C. Shu, P. Li, S. Palanathan, C. Cheng Kao, Single nucleotide polymorphisms of human STING can affect innate immune response to cyclic dinucleotides. *PLOS ONE* **8**, e77846 (2013). doi:10.1371/journal.pone.0077846 Medline

49. H. Konno, S. Yamauchi, A. Berglund, R. M. Putney, J. J. Mulé, G. N. Barber, Suppression of STING signaling through epigenetic silencing and missense mutation impedes DNA damage mediated cytokine production. *Oncogene* **37**, 2037–2051 (2018). doi:10.1038/s41388-017-0120-0 Medline

50. J. P. Leonard, M. L. Sherman, G. L. Fisher, L. J. Buchanan, G. Larsen, M. B. Atkins, J. A. Sosman, J. P. Dutcher, N. J. Vogelzang, J. L. Ryan, Effects of single-dose interleukin-12 exposure on interleukin-12-associated toxicity and interferon-γ production. *Blood* **90**, 2541–2548 (1997). Medline

51. X. Sun, X. Zhou, X. Shi, O. A. Abed, X. An, Y. L. Lei, J. J. Moon, Strategies for the development of metalloimmunotherapies. *Nat. Biomed. Eng.* **8**, 1073–1091 (2024). doi:10.1038/s41551-024-01221-7 Medline

52. S. J. Allison, J. Bryk, C. J. Clemett, R. A. Faulkner, M. Ginger, H. B. S. Griffiths, J. Harmer, P. Jane Owen-Lynch, E. Pinder, H. Wurdak, R. M. Phillips, C. R. Rice, Self-assembly of an anion receptor with metal-dependent kinase inhibition and potent in vitro anti-cancer properties. *Nat. Commun.* **12**, 3898 (2021). doi:10.1038/s41467-021-23983-3 Medline

53. R. Pothiraja, P. Rajakannu, P. Vishnoi, R. J. Butcher, R. Murugavel, Polymeric and cyclic manganese phosphates and phosphinates: Synthesis, spectral characterization and solid-state structures. *Inorg. Chim. Acta* **414**, 264–273 (2014). doi:10.1016/j.ica.2014.01.038

54. A. Masunov, T. Lazaridis, Potentials of mean force between ionizable amino acid side chains in water. *J. Am. Chem. Soc.* **125**, 1722–1730 (2003). doi:10.1021/ja025521w Medline

55. X. Zhou, X. Ling, X. Sun, Z. Wan, T. Dwyer, T. Moore, Q. Li, H. Dobson, Q. Wu, X. Kong, F. Xie, X. An, J. Gan, K. Wang, Y. S. Cho, W. Gong, K. Dong, J. Zhang, M. Takahashi, C. Xu, S. Kodamasimham, J. Xu, V. Yuzbasiyan-Gurkan, S. Chinn, A. Schwendeman, S. Glotzer, Y. Lei, J. Moon, Intermetallic Nanoassemblies Potentiate Systemic STING Activation [Dataset], Dryad (2025); https://doi.org/10.5061/dryad.qz612jmw8.

56. T. Dwyer, T. C. Moore, S. C. Glotzer, X. Zhou, Simulation dataset for: Intermetallic Nanoassemblies Potentiate Systemic STING Activation [Data set], University of Michigan–Deep Blue Data (2025); https://doi.org/10.7302/zzaj-ak19.

57. A. B. Nair, S. Jacob, A simple practice guide for dose conversion between animals and human. *J. Basic Clin. Pharm.* **7**, 27–31 (2016). doi:10.4103/0976-0105.177703 Medline
